# Impact of a positive end-expiratory pressure strategy on oxygenation, respiratory compliance, and hemodynamics during laparoscopic surgery in non-obese patients: a systematic review and meta-analysis of randomized controlled trials

**DOI:** 10.1186/s12871-023-02337-0

**Published:** 2023-11-11

**Authors:** Gulfairus A. Yessenbayeva, Yekaterina A. Yukhnevich, Zaukiya K. Khamitova, Sergey I. Kim, Murat B. Zhumabayev, Gulbanu S. Berdiyarova, Sanzhar B. Shalekenov, Irina Y. Mukatova, Andrey I. Yaroshetskiy

**Affiliations:** 1 National Research Oncology Center, Astana, Kazakhstan; 2 Karaganda Medical University, Karaganda, Kazakhstan; 3 Multidisciplinary hospitals named after Professor H.J.Makazhanov, Karaganda, Kazakhstan; 4 Astana Medical University, Astana, Kazakhstan; 5 Kazakh-Russian Medical University, Almaty, Kazakhstan; 6 Pulmonology Department, Sechenov First Moscow State Medical University (Sechenov University, 8/2, Trubetskaya str. 119991, Moscow, Russia

**Keywords:** Positive end-expiratory pressure, PEEP, Laparoscopic Surgery, Lung protective ventilation, Compliance, Oxygenation, Non-obese, Pneumoperitoneum, Meta-analysis

## Abstract

**Background:**

Higher positive end-expiratory pressure (PEEP) during laparoscopic surgery may increase oxygenation and respiratory compliance. This meta-analysis aimed to compare the impact of different intraoperative PEEP strategies on arterial oxygenation, compliance, and hemodynamics during laparoscopic surgery in non-obese patients.

**Methods:**

We searched RCTs in PubMed, Cochrane Library, Web of Science, and Google Scholar from January 2012 to April 2022 comparing the different intraoperative PEEP (Low PEEP (LPEEP): 0–4 mbar; Moderate PEEP (MPEEP): 5–8 mbar; high PEEP (HPEEP): >8 mbar; individualized PEEP - iPEEP) on arterial oxygenation, respiratory compliance (Cdyn), mean arterial pressure (MAP), and heart rate (HR). We calculated mean differences (MD) with 95% confidence intervals (CI), and predictive intervals (PI) using random-effects models. The Cochrane Bias Risk Assessment Tool was applied.

**Results:**

21 RCTs (n = 1554) met the inclusion criteria. HPEEP vs. LPEEP increased PaO_2_ (+ 29.38 [16.20; 42.56] mmHg, p < 0.0001) or PaO_2_/FiO_2_ (+ 36.7 [+ 2.23; +71.70] mmHg, p = 0.04). HPEEP vs. MPEEP increased PaO_2_ (+ 22.00 [+ 1.11; +42.88] mmHg, p = 0.04) or PaO_2_/FiO_2_ (+ 42.7 [+ 2.74; +82.67] mmHg, p = 0.04). iPEEP vs. MPEEP increased PaO_2_/FiO_2_ (+ 115.2 [+ 87.21; +143.20] mmHg, p < 0.001). MPEEP vs. LPEP, and HPEEP vs. MPEEP increased PaO_2_ or PaO_2_/FiO_2_ significantly with different heterogeneity. HPEEP vs. LPEEP increased Cdyn (+ 7.87 [+ 1.49; +14.25] ml/mbar, p = 0.02). MPEEP vs. LPEEP, and HPEEP vs. MPEEP did not impact Cdyn (p = 0.14 and 0.38, respectively). iPEEP vs. LPEEP decreased driving pressure (-4.13 [-2.63; -5.63] mbar, p < 0.001). No significant differences in MAP or HR were found between any subgroups.

**Conclusion:**

HPEEP and iPEEP during PNP in non-obese patients could promote oxygenation and increase Cdyn without clinically significant changes in MAP and HR. MPEEP could be insufficient to increase respiratory compliance and improve oxygenation. LPEEP may lead to decreased respiratory compliance and worsened oxygenation.

**Prospero registration:**

CRD42022362379; registered October 09, 2022.

**Supplementary Information:**

The online version contains supplementary material available at 10.1186/s12871-023-02337-0.

## Introduction

Pneumoperitoneum (PNP) and the position of the patient required for laparoscopic surgery lead to pathophysiological changes that complicate anesthesia [1]. PNP is characterized by an increased intra-abdominal pressure (IAP), the cranial displacement of the diaphragm that can lead to the formation of intraoperative atelectasis and decrease end-expiratory lung volume (EELV) [2, 3]. At the same time, PNP can reduce respiratory system compliance by 30–50% in healthy patients [4, 5]. During elective abdominal surgery under general anesthesia, atelectasis forms in almost 90% of patients [6] and can become a focus of postoperative pneumonia. And one of the methods to avoid the effects of PNP on lung tissue is to apply positive end-expiratory pressure (PEEP) [7]. PEEP is acknowledged as a component of lung protective ventilation (LPV) along with low tidal volume (TV) 6–8 ml/kg [8, 9]. On the other hand, excessive PEEP can lead to the overdistension of lung tissue and cause volutrauma [10] and hemodynamic instability. It is necessary to use sufficient PEEP to minimize atelectasis, improve respiratory biomechanics and maintain oxygenation.

A recent systematic review and meta-analysis of intensive care unit (ICU) patients without acute respiratory distress syndrome (ARDS) found no reduction in in-hospital mortality or reduced ventilation duration in patients with higher PEEP levels. However, hypoxemia and ARDS occurred less frequently with higher PEEP (assessed by arterial partial oxygen pressure (PaO_2_) or PaO_2_/FiO_2_ index) [11]. In a large observational study in non-obese general surgery patients, a PEEP of 5 cmH_2_O was identified as a protective factor associated with fewer postoperative pulmonary complications (PPL) [12]. In addition, zero PEEP was associated with worse outcomes, including increased hypoxemia, ventilator-associated pneumonia, and in-hospital mortality [13]. Just one systematic review and network meta-analysis suggested that individualized PEEP combined with a recruitment maneuver (RM) may be an optimal ventilation strategy combined with low tidal volumes in abdominal surgery, but it uses a mixed population of laparoscopic and open surgery patients [14]. A higher PEEP may be used in obese patients, as some studies indicate impaired respiratory biomechanics in this group of patients [15, 16]. Although low tidal volume has been recognized as a protective tool during surgery, RCTs comparing PEEP levels during laparoscopic surgery have been small and shown conflicting results on the effects of PEEP on oxygenation, respiratory mechanics, and hemodynamic stability [14, 18, 19, 21–38]. Not a single meta-analysis has examined the effect of PEEP on oxygenation, respiratory mechanics, or hemodynamics in laparoscopic surgery neither in obese nor non-obese patients. So, the optimal level of PEEP during laparoscopic surgery without lung injury is still not clear and debatable. We conducted a systematic review and meta-analysis to compare the impact of different intraoperative PEEP strategies on oxygenation, compliance, and hemodynamic parameters during laparoscopic surgery in non-obese patients.

## Methods

We performed a systematic review and meta-analysis following the Preferred Reporting Items for Systematic Review and Meta-analysis (PRISMA) [17]. The protocol for this meta-analysis was pre-registered to the International Prospective Registry of Systematic Reviews database (CRD42022362379; registered October 09, 2022).

### Search strategy

We searched RCTs in English, which studied the effect of different levels of PEEP on blood oxygenation, respiratory compliance, and hemodynamics in non-obese patients during laparoscopic surgery. The studies were found through electronic searches of the PubMed, Cochrane Library, Web of Science, and Google Scholar databases by two researchers who did not go through the details of the studies. We limited our review to studies published in the last ten years (January 2012 to April 2022) because intraoperative ventilation practices and intervention techniques have changed over the past decade. All articles found on this platforms have been analyzed for relevance by title and abstract. For potentially relevant articles, full-text articles were obtained for analysis. From these articles, as well as from related reviews and meta-analyses, all links and potentially relevant titles have been manually checked.

The following search terms or their combination were used during the search:

Keywords: (((((((((((((“Tidal Volume“[Mesh]) OR Tidal Volumes) OR Volume, Tidal) OR Volumes, Tidal))) OR ((((((((((((((((((“Positive-Pressure Respiration“[Mesh]) OR Positive-Pressure Respiration) OR Positive-Pressure Respirations) OR Respiration, Positive Pressure) OR Respirations, Positive-Pressure) OR Positive Pressure Ventilation) OR Positive-Pressure Ventilation) OR Positive-Pressure Ventilations) OR Ventilation, Positive Pressure) OR Ventilations, Positive-Pressure) OR Positive End-Expiratory Pressure) OR End-Expiratory Pressure, Positive) OR End-Expiratory Pressures, Positive) OR Positive End-Expiratory Pressure) OR Positive End-Expiratory Pressures) OR Pressure, Positive End-Expiratory) OR Pressures, Positive End-Expiratory))))) AND Randomized Controlled Trial[Publication Type]) NOT (((animals [Mesh] not (humans [Mesh] and animals [Mesh])))))))) AND laparoscopic.

Four researchers independently extracted data into the database developed for this dataset. Disagreements about data extraction were resolved through discussion.

### Selection criteria

We included studies with the following PICOS criteria:

1. Population: non-obese adult patients who underwent general anesthesia with mechanical ventilation with tidal volumes ≤ 10 ml/kg during laparoscopic surgery in the past ten years.
2. Intervention: PEEP level during mechanical ventilation.
3. Comparison: the lung ventilation strategies were divided by PEEP levels according to most common stratification in the included studies: (low PEEP (LPEEP): 0–4 mbar; moderate PEEP (MPEEP): 5–8 mbar; high PEEP (HPEEP): >8 mbar; individualized PEEP (iPEEP): PEEP set by special physiological technique - electrical impedance tomography or transpulmonary pressure).
4. Outcomes: Arterial partial pressure of oxygen (PaO_2_) or PaO_2_ to Inspiratory oxygen fraction ratio (PaO_2_/FiO_2_), dynamic respiratory compliance, mean arterial pressure, heart rate.
5. Study design: randomized controlled trial.

We eliminated studies that were not written in English, not in full-text format, and studies where mechanical ventilation with a laryngeal mask was used for general anesthesia.

### Data extraction

The main goal was to compare the effect of different PEEP strategies on oxygenation and respiratory compliance in non-obese adult patients who underwent general anesthesia with mechanical ventilation during laparoscopic surgery. The secondary objective was to compare the effect of different PEEP strategies on heart rate and mean arterial pressure in non-obese adult patients undergoing general anesthesia with mechanical ventilation during laparoscopic surgery.

The oxygenation was assessed by intraoperative measurement of the arterial partial pressure of oxygen (PaO_2_), or arterial oxygen partial pressure to fractional inspired oxygen (PaO_2_/FiO_2_) ratio) calculation. We evaluated respiratory compliance by dynamic compliance (Cdyn) or driving pressure (DP) measurements, taking into account that Cdyn represents not only the elastance of the respiratory system but also the airway resistance resulting from periodic recruitment/derecruitment of alveoli and small airways [18, 19]. The hemodynamics was assessed by noninvasive measurement of mean arterial pressure (MAP) and heart rate (HR).

If data were available only in graphical format, GetData Graph Digitizer 2.25 (http://getdata-graph-digitizer.com/) was used to quantify the data.

### Statistical analysis

Data analysis was performed using two software applications: Review Manager software (RevMan, version 5.4)” and Stata 17.0 (StataCorp, College Station, TX, USA). Pooled continuous outcomes were reported as the mean difference (MD) with 95% confidence intervals (CI), standardized mean difference (SMD). We used 95% predictive intervals (PI) for the description of the true effect value within studies. A random effect model was taken due to the expected between-study heterogeneity. Heterogeneity was assessed using observed weighted sum of squares (Chi^2^), variance of the true effect size (Tau^2^), and the ratio of excess dispersion to total dispersion (I^2^). P value < 0.10 for Chi^2^ considered that the true effect varies. In order to check for the existence of publication bias, a funnel plot graph was designed in this meta-analysis. Sensitivity analysis was performed by excluding one study at a time to analyze a possible chain of results. We used meta-regression for evaluation of the influence of tidal volume value and body position during surgery (Trendelenburg or reverse Trendelenburg) on study outcomes, calculated test for residual homogeneity (Qres), regression coefficient, R^2^ for the proportion of between studies variance explained by the covariates, and drew bubble plots.

### Quality assessment

The Cochrane Risk of Bias Assessment Tool (RoB 2.0) was used to assess the quality of the included studies in five domains: randomization process [D1], deviation from intended interventions [D2], missing outcome data [D3], outcome measurement [D4], and selection of reported results [D5] [20]. In addition, each domain was rated as high risk, low risk, or some concern using the Cochrane Criteria for assessing the risk of bias [20].

## Results

### Studies characteristics

**Fig. 1:**
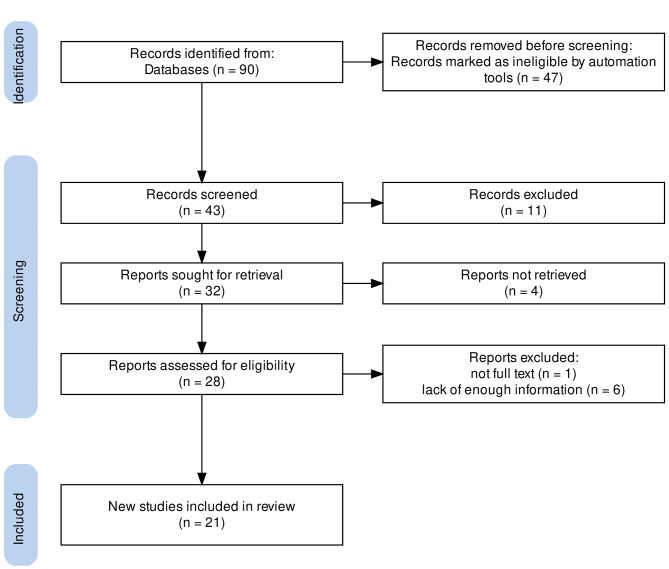
PRISMA flow chart of the included studies

A total of 90 unique studies were identified, of which 21 RCTs published between 2012 and 2022 met the inclusion criteria and were included in the study (Fig. 1). These RCTs included 1554 non-obese patients undergoing laparoscopic surgery at baseline and receiving mechanical ventilation in volume-controlled mode with different levels of PEEP. These studies aimed to evaluate the effect of different PEEP strategies on oxygenation (PaO_2_ or PaO_2_/FiO_2_), Cdyn, MAP and HR during PNP in non-obese subjects undergoing laparoscopic surgery. It should be noted that in the LPEEP groups, almost all studies used PEEP = 0 (zero end-expiratory pressure - ZEEP), with the exception of the study by Chun EH et al. [38], which used a PEEP of 4 mbar in the LPEEP group.

15 RCTs (n = 784) measured PaO_2_, 12 RCTs (n = 735) evaluated PaO_2_/FiO_2_, 12 RCTs (n = 935) measured Cdyn, 17 RCTs (n = 974) MAP, and 16 RCTs (n = 862) HR. The types of procedures included laparoscopic cholecystectomy, laparoscopic colon cancer resection, laparoscopic radical gastrectomy, laparoscopic gynaecologic surgery, robot-assisted laparoscopic radical prostatectomy, and robot-assisted gynaecologic surgery. Detailed baseline characteristics of the included studies are presented in Table 1 [14, 18, 19, 21–38].

In 17 RCTs (n = 607) non-obese subjects undergoing laparoscopic surgery during PNP received low PEEP, in 19 RCTs (n = 667) moderate PEEP, in 9 RCTs (n = 202) high PEEP, and 6 RCTs (n = 118) individualized PEEP (iPEEP solved by a titration strategy).

**Table 1:** Study characteristics

Study	Study design	Surgery	Position	N total	TV (ml/kg)	PEEP (cmH2O)	Mean age (years)	BMI (kg/m2)	n	ASA	Strategy classification	HD complications	RM
Kwak HJ et al. (2012)	Single centre	Laparoscopic cholecystectomy	RT	60	8	0	41.8 ± 9.3	< 30 (24,1)	30	I	LPEEP	NB	
					8	10	43.3 ± 12.0	< 30 (24,3)	30		HPEEP		
Russo A et al. (2013)	single centre	pelvic laparoscopic surgery	T	60	8	0	32.2 ± 5.2	22.6 ± 1.2	20	I	LPEEP	NB	
					8	5	34.0 ± 4.8	22.1 ± 1.5	20		MPEEP		
					8	10	33.4 ± 5.3	22.3 ± 1.7	20		HPEEP		
Baki ED et al. (2014)	Single centre	Laparoscopic surgery	RT, T	60	10	0	50.2 ± 13.9	< 30	30	I–II	LPEEP	NB	
					6	5	54.5 ± 15.4	< 30	30		MPEEP		
Ela Y et al. (2014)	Single centre	Laparoscopic urologic procedures	T	81	10	0	61.5 ± 12.5	25.6 ± 2.5	38	I–III	LPEEP	NB	
					6	5	59.2 ± 11.3	25.7 ± 3.2	43		MPEEP		
Kundra P et al. (2014)	Single centre	Laparoscopic cholecystectomy	RT	75	10	0	45.1 ± 11	25.0	25	I–II	LPEEP	NB	
					10	5	40.4 ± 10.8	23.6	25		MPEEP		
					10	10	45.4 ± 10.0	23.7	25		HPEEP		
Karabayirli S et al. (2016)	Single centre	Laparoscopic cholecystectomy	RT	40	8	0	42.0 ± 13.8	27.3 ± 4.6	20	I–II	LPEEP	NB	
					8	10	42.6 ± 12.0	26.8 ± 5.1	20		HPEEP		
He X et al. (2016)	Single centre	Laparoscopic surgery (rectectomy or colectomy)	T	42	6	PEEP titration	54.5 ± 7.3	24.0 ± 3.4	23	I–II	iPEEP	2 patients underwent hypotension (MAP < 50 mm Hg for more than 3 min requiring vasoactive drugs)	sustained inflation of the lungs for 40 s to a peak inspiratory pressure (PIP) of 35 cm H2O,16 was done every 45 min.
					6	PEEP EIT	56.7 ± 5.6	22.8 ± 3.0	19		iPEEP		
Sen O et al. (2017)	Single centre	Laparoscopic cholecystectomy	RT	43	8	5	49.0 ± 13.5	< 30	20	I–II	MPEEP	NB	
					8	10	43.5 ± 12.6	< 30	23		HPEEP		
Chin JH et al. (2017)	single centre	robot-assisted laparoscopic radical prostatectomy	T	38	8	0	65.1 ± 6.9	25.7 ± 3.1	19		LPEEP	NB	
					8	8	63.0 ± 6.8	25.5 ± 3.5	19		MPEEP		
Wang Y et al. (2019)	Single centre	Laparoscopic radical rectectomy or colectomy	T	54	6–8	0	55.0 ± 10.0	24.3 ± 3.0	15	I–II	LPEEP	three patients in PEEP 12 were excluded because of the hypotension (mean arterial pressure < 65 mmHg) that could not be corrected by vasoactive agents (e.g., ephedrine, phenylephrine, perdipine).	
					6–8	4	56.0 ± 12.0	22.8 ± 2.0	15		MPEEP		
					6–8	8	52.0 ± 13.0	23.8 ± 2.1	14		MPEEP		
					6–8	12	53.0 ± 9.0	24.0 ± 2.8	10		HPEEP		
Liu J et al. (2019)	Single centre	Laparoscopic radical gastrectomy	RT	115	10	0	66.1 ± 9.1	23.2 ± 2.9	57	I–III	LPEEP	NB	using the sustained airway pressure obtained by the CPAP method and applying 30 cm H2O PEEP for 30 s followed by a decremental PEEP titration procedure directed by static pulmonary compliance (Cstat)
					7	6	63.2 ± 8.3	22.4 ± 2.1	58		MPEEP		
Chun EH et al. (2019)	Single centre	Elective robotic gynaecological surgery	T	40	6	4	41.0 ± 9.9	22.4 ± 3.6	20	I–II	LPEEP	NB	
					6	8	37.4 ± 7.8	23.4 ± 3.0	20		MPEEP		
You AH et al. (2019)	Single centre	Robot-assisted laparoscopic radical prostatectomy	T	50	8	0	65.3 (7.5)	< 30	25		LPEEP	NB	
					8	5	65.7 (7.4)	< 30	25		MPEEP		
Atashkhoei S et al. (2020)	single centre	gynecologic laparoscopy	T	60	10	0	30.0 ± 7.4	< 30	20	I	LPEEP	After PNP was dysrhythmia in nine patients (15%), mostly in the ZEEP and PEEP5 groups. P = 0.004)	
					10	5	28.3 ± 6.5	< 30	20		MPEEP		
					10	10	32.4 ± 7.4	< 30	20		HPEEP		
Shono A et al. (2020)	Single centre	Robot-assisted laparoscopic prostatectomy	T	48	6–8	5	66.0 ± 7.0	23.0 ± 2.0	25	I–II	MPEEP	NB	The peak inspiratory pressure gradient (above PEEP) was set at 20 cm H2O, and PEEP was progressively increased every three breaths from 5 to 20 in steps of 5 cm H2O to obtain a stepwise increase of peak inspiratory to 30, 35, and 40 cm H2O. The final recruiting pressure of 40 cm H2O was applied for six breaths. The recruitment was completed within 90 s
					6–8	15	67.0 ± 5.0	24.0 ± 2.0	23		HPEEP		
Piriyapatsom A et al. (2020)	Single centre	Laparoscopic Gynaecological surgery	T	44	8	5	41.3 ± 5.7	23.9 ± 4.9	22	I–II	MPEEP	Two patients in the iPEEP and one in the MPEEP developed hypotension after 5 min PNP	
					8	PEEP Pes	41.0 ± 9.3	22.7 ± 2.6	22		iPEEP		
Cammarota G et al. (2020)	Single centre	Elective pelvic robotic surgery	T	28	6–8	5	62.5 ± 9.5	25.4 ± 2.6	14	I–II	MPEEP	NB	
					6–8	PEEP Pes	62.8 ± 11.4	24.1 ± 4.1	14		iPEEP		
Girrbach F et al. (2020)	Single centre	Robot-assisted laparoscopic radical prostatectomy	T	40	8	5	64.2 (50–76)	25.6 (2.5)	20	I–III	MPEEP	Blood pressure complications iPEEP 8 (40%) and MPEEP 5 (25%) p = 0.50. Norepinephrine infusion 18(90%) and 15 (75%) p = 0.41 respectively.	
					8	PEEP EIT	62.6 (49–76)	25.3 (2.3)	20		iPEEP		
Li H et al. (2021)	Single centre	Laparoscopic colorectal cancer resection	T	260	6–8	0	70.8 ± 5.8	22.3 ± 2.8	130	II–III	LPEEP	More patients in the OLS group than the non-OLS group developed potentially harmful hypotension [OLS vs. non-OLS, 21 (15%) vs. 6 (4.3%); P¼0.004] and needed vasopressors [35 (25%) vs. 12 (8.6%); P < 0.001] intra-operatively.	A stepwise increment of tidal volume was used for each LRM
					6–8	6–8	69.7 ± 5.8	23.0 ± 2.7	130		MPEEP		
Nguyen TK et al. (2021)	Single centre	Laparoscopic surgery (gastrectomy, colectomy, Miles’ operation, LAR surgery, others)	RT, T	62	10	0	55.0 ± 12.0	21.0 ± 3.0	31	I–III	LPEEP	NB	RM alveoli were recruited applying a stepwise increase in PEEP (from 4 to 10 cmH2O for 3 breaths, 10 to 15 cmH2O for 3 breaths, and 15 to 20 cmH2O for 10 breaths) with maximum PIP (Peak Inspiratory Pressure) of 50 cmH2O
					7	10	59.0 ± 9.0	21.0 ± 2.0	31		HPEEP		
Huang D et al. (2021)	Single centre	Robot-assisted laparoscopic radical cystectomy	T	254	6	0	66 (59–73)	24.0 (3.2)	127	I–III	LPEEP	NB	
					9	7	64 (59–72)	23.6 (3.0)	127		MPEEP		

**Abbreviations**: ASA: American Society of Anaesthesiologists physical status; BMI: body mass index; EIT: electrical impedance tomography; iPEEP: individualized positive end-expiratory pressure group; LPEEP: low positive end-expiratory pressure group; MPEEP: moderate positive end-expiratory pressure group; HPEEP: high positive end-expiratory pressure group; PEEP: positive end-expiratory pressure; Pes: esophageal pressure; TV: tidal volume; T: Trendelenburg; RT: reverse Trendelenburg;

### Evidence quality and the risk of bias

**Fig. 2:**
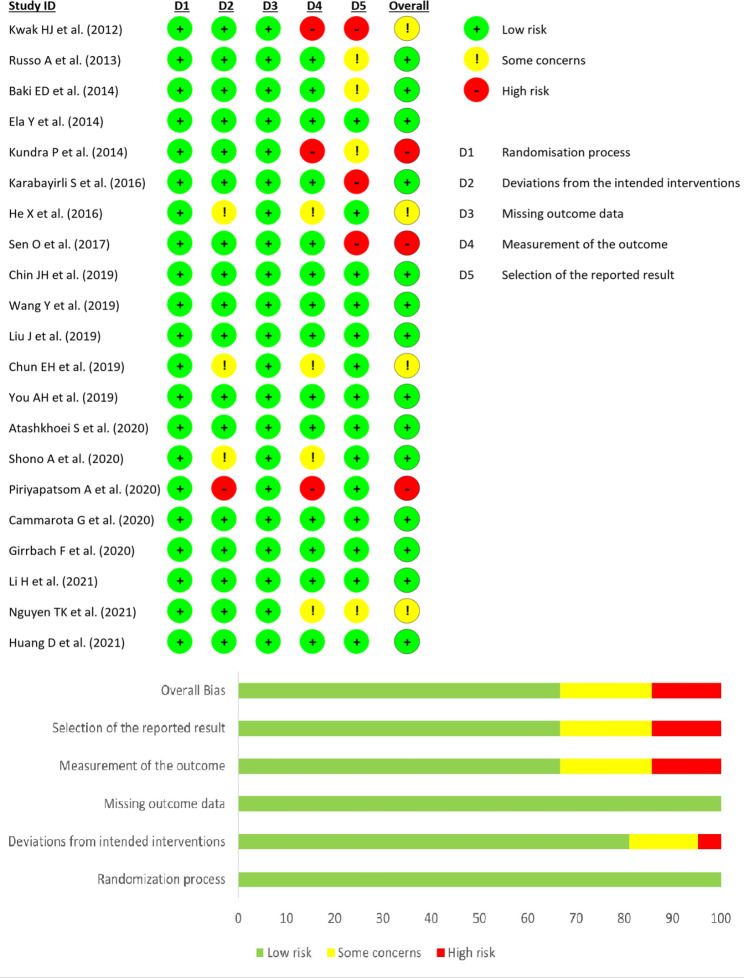
Cochrane risk of bias assessment tool

All the studies presented low risk in terms of random sequence generation (Fig. 2). Due to the completeness of the outcome data, the risks of attrition bias were likewise evaluated as low. Three trials did not provide information on deviations from intended interventions [27, 32, 38], and one trial had a high risk of deviation bias [33]. Four studies did not report the measurement of the outcome [27, 32, 36, 38] and three studies showed a high risk of measurement of the outcome bias [21, 25, 33]. Three studies showed an increased risk of “selection of the reported result” bias [21, 26, 28] and four trials had some concerns [22, 23, 25, 36]. For details see Supplement 1&2.

### Oxygenation

In included studies, the authors used different methods to evaluate the PEEP on oxygenation: some studies compared the effect of the PEEP strategy on PaO_2_, while others - on PaO_2_/FiO_2_.

The vast majority of studies used PaO_2_ as a method for oxygenation evaluation: 8 RCTs (n = 419) compared LPEEP and MPEEP, 4 RCTs (n = 192) - LPEEP and HPEEP, and 4 RCTs (n = 173) - MPEEP and HPEEP. Three of those 8 RCTs simultaneously compared LPEEP, MPEEP, and HPEEP. All data regarding PaO_2_ are described in the Supplement 1 since PaO_2_ is not a reliable measure of oxygenation without information on FiO_2_ (Fig. S1 - Fig. S7). In general, all comparisons (HPEEP vs. LPEEP, HPEEP vs. MPEEP, and MPEEP vs. LPEEP) showed an increase in PaO_2_ with different heterogeneity.

**Fig. 3:**
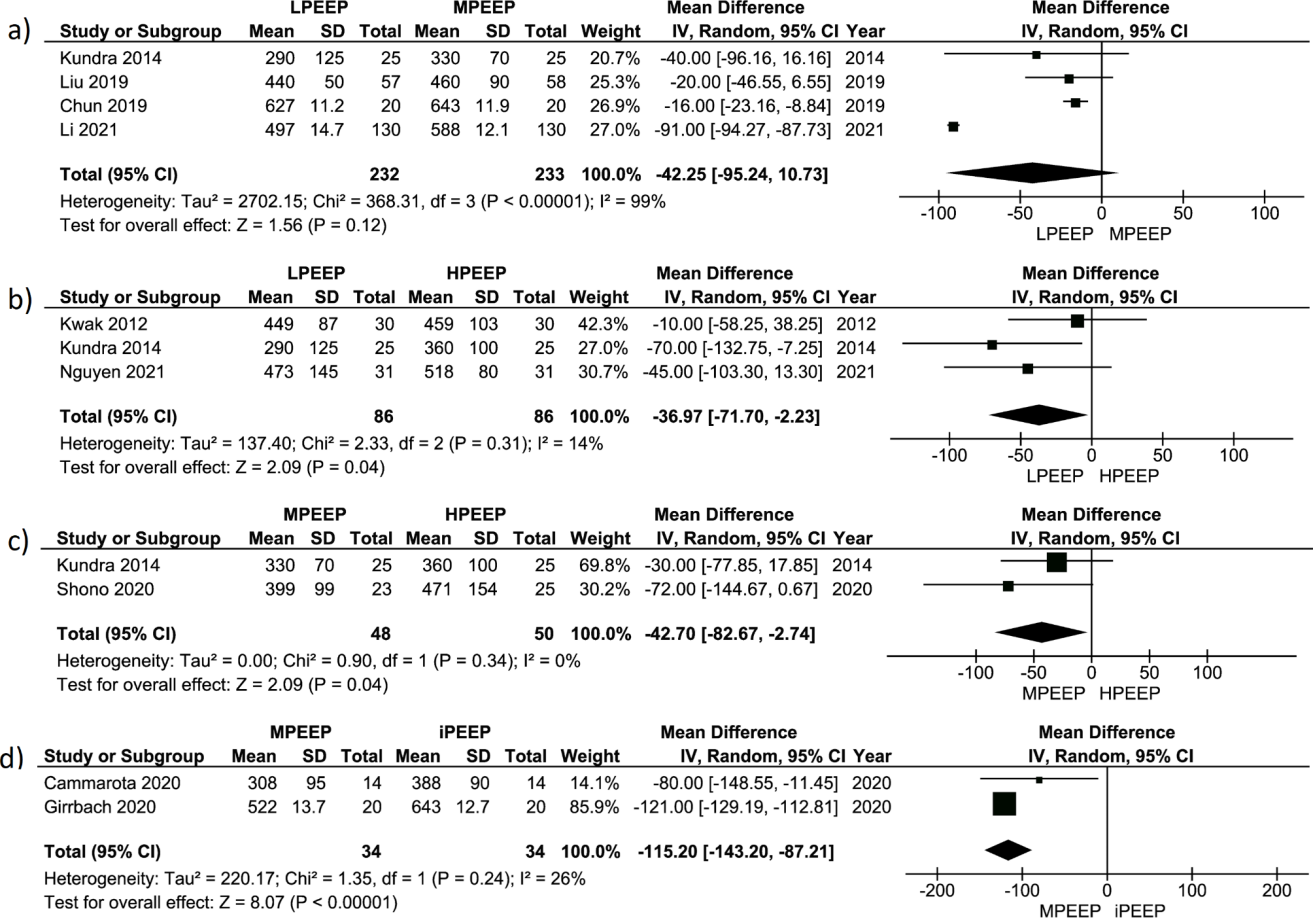
Forest plot for PaO_2_/FiO_2_ comparing different PEEP strategy groups: **(a)** LPEEP vs. MPEEP; **(b)** LPEEP vs. HPEEP; **(c)** MPEEP vs. HPEEP; **(d)** MPEEP vs. iPEEP. Data are presented as mean differences and 95% confidence intervals. The vertical line represents no effect with the value of 0. The diamond represents the pooled mean effect estimate with 95% CI. It provides an overall measure of the difference in PaO_2_/FiO_2_ values between different PEEP strategy groups. Abbreviations: CI: confidence interval; SD: standard deviation; I^2^: the ratio of excess dispersion to total dispersion; Тau^2^: the variance of the true effect sizes; Chi^2^: observed weighted sum of squares; df: degrees of freedom; PaO_2_/FiO_2_: arterial oxygen partial pressure to fractional inspired oxygen ratio; LPEEP: low positive end-expiratory pressure group; MPEEP: moderate positive end-expiratory pressure group; HPEEP: high positive end-expiratory pressure group; iPEEP: individualized positive end-expiratory pressure group

Meta-analysis of 4 studies comparing the influence of LPEEP vs. MPEEP (n = 465) on PaO_2_/FiO_2_ did not show a significant increase in PaO_2_/FiO_2_ (+ 42.25 (-10.73; 95.24) mmHg, p < 0.12). Also, the meta-analysis found high variability in true effect between studies (Chi^2^ 368.31, p < 0.001). The distribution of true effect size was wide (T^2^ = 2702.15), and I^2^ 99%, which can correspond to a high real proportion of true effect variation (Fig. 3a). Estimation of the prediction interval of true effect also showed a very wide distribution crossing zero line (Fig. S8). But we found high risk of publication bias (Fig. S9). A different picture was seen in the meta-analysis of 3 studies comparing influence of LPEEP (all included studies used ZEEP) vs. HPEEP (all included studies used 10 mbar) (n = 172) on PaO_2_/FiO_2_. It revealed a substantial increase in PaO_2_/FiO_2_ in HPEEP (PEEP 10 mbar) group (+ 36.7 (+ 2.23; +71.70) mmHg, p = 0.04) but did not find significant variability in true effect between studies (Chi^2^ 2.33, p = 0.31). I^2^ was only 14%, corresponding to low real proportion of true effect variation, but the high proportion of sampling error (Fig. 3b). Estimation of the predictive interval of true effect also showed very wide distribution crossing zero line (Fig. S10). The risk of publication bias was low (Fig. S11). The meta-analysis of only 2 studies comparing the influence of MPEEP vs. HPEEP (n = 98) on PaO_2_/FiO_2_ revealed a significant increase in PaO_2_/FiO_2_ in HPEEP group (+ 42.7 (+ 2.74; +82.67) mmHg, p = 0.04). We did not find variations in true effect between studies (Chi^2^ 2.33, p = 0.31). Due to Chi^2^ being less than df, the variation of true effect size (T^2^) is zero. Almost all dispersion of PaO_2_ between MEEP and HPEEP can be attributed to sampling error (I^2^ = 0%) (Fig. 3c). The risk of publication bias was low also (Fig. S12). Two studies compared MPEEP (all studies used 5 mbar) with individual PEEP (iPEEP) (n = 68) in a robotic pelvic surgery: one study used an esophageal pressure-guided setting of PEEP [34], while another was an electrical impedance-guided PEEP. The meta-analysis of these studies showed a significant increase in PaO_2_/FiO_2_ in iPEEP group (+ 115.2 (+ 87.21; +143.20) mmHg, p < 0.001). We did not find variations in true effect between studies (Chi^2^ 1.35, p = 0.24), but high variation of true effect size (T^2^ = 220.17). Almost all dispersion of PaO_2_ between MEEP and iPEEP can be attributed to sampling error (I^2^ = 26%) (Fig. 3d). The risk of publication bias was high (Fig. S13).

### Dynamic respiratory compliance and driving pressure

Most of the included studies (N = 10) measured dynamic compliance (Cdyn) for respiratory compliance evaluation. Six RCTs (n = 746) compared LPEEP and MPEEP, four RCTs (n = 189) - LPEEP and HPEEP, and two RCTs (n = 64) - MPEEP and HPEEP. One of those RCTs simultaneously compared LPEEP, MPEEP, and HPEEP.

**Fig. 4:**
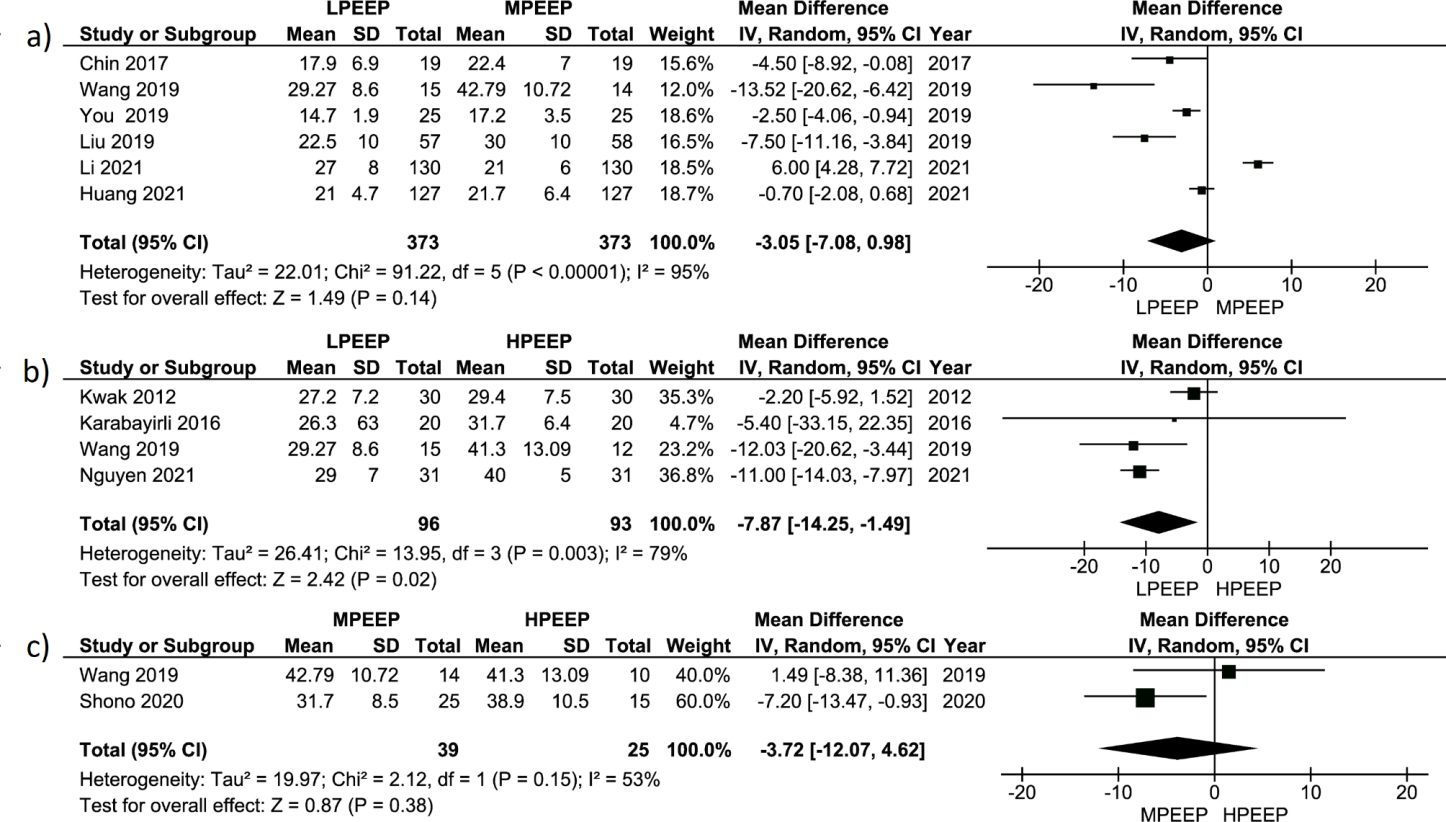
Forest plot for Cdyn comparing different PEEP strategy groups: **(a)** LPEEP vs. MPEEP; **(b)** LPEEP vs. HPEEP; **(c)** MPEEP vs. HPEEP. Data are presented as mean differences and 95% confidence intervals. The vertical line represents no effect with the value of 0. The diamond represents the pooled mean effect estimate with 95% CI. It provides an overall measure of the difference in Cdyn values between different PEEP strategy groups. Abbreviations: CI: confidence interval; SD: standard deviation; I^2^: the ratio of excess dispersion to total dispersion; Тau^2^: the variance of the true effect sizes; Chi^2^: observed weighted sum of squares; df: degrees of freedom; Cdyn: dynamic compliance; LPEEP: low positive end-expiratory pressure group; MPEEP – moderate positive end-expiratory pressure group; HPEEP: high positive end-expiratory pressure group

Meta-analysis of 6 studies comparing the influence of LPEEP (all included studies used ZEEP) vs. MPEEP (n = 746) on Cdyn did not show a significant increase in Cdyn in MPEEP group (+ 3.05 (-3.05; +7.08) ml/mbar, p = 0.14). Also, meta-analysis found high variability in true effect between studies (Chi^2^ 91.22, p < 0.001). The distribution of true effect size was wide (T^2^ = 22.01), and I^2^ 95%, which can correspond to a high real proportion of true effect variation (Fig. 4a). Estimating the prediction interval of true effect also showed broad distribution crossing zero line (Fig. S14). We found a high risk of publication bias (Fig. S15).

On the opposite, the meta-analysis of 4 studies comparing the influence of LPEEP vs. HPEEP (n = 189) on Cdyn revealed a significant increase in Cdyn in HPEEP group (+ 7.87 (+ 1.49; +14.25) ml/mbar, p = 0.02) with considerable variability in true effect between studies (T^2^ = 26.41, Chi^2^ 13.95, p = 0.003). I^2^ was 79%, corresponding to a high real proportion of true effect variation (Fig. 4b). Estimation of the prediction interval of true effect also showed extensive distribution crossing zero line (Fig. S16). The risk of publication bias was high (Fig. S17). The meta-analysis of 2 studies comparing the influence of MPEEP vs. HPEEP (n = 64) on Cdyn did not find a significant increase in Cdyn in HPEEP (PEEP 10 mbar) group (+ 3.72 (-4.52; +12.07) ml/mbar, p = 0.38). We did not find significant variability in true effect between studies (T^2^ = 19.97; Chi^2^ 2.12, p = 0.15) may be due to low power. I^2^ was 53%, corresponding to a high proportion of true effect variation, and sampling error (Fig. 4c). The risk of publication bias was low (Fig. S18).

**Fig. 5:**
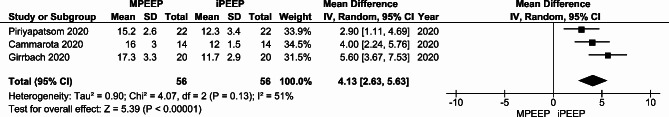
Forest plot for DP (as a surrogate of Cdyn) comparing MPEEP vs. iPEEP groups. Data are presented as mean differences and 95% confidence intervals. The vertical line represents no effect with the value of 0. The diamond represents the pooled mean effect estimate with 95% CI. It provides an overall measure of the difference in DP values between different PEEP strategy groups. Abbreviations: CI: confidence interval; SD: standard deviation; I^2^: the ratio of excess dispersion to total dispersion; Тau^2^: the variance of the true effect sizes; Chi^2^: observed weighted sum of squares; df: degrees of freedom; DP: driving pressure; Cdyn: dynamic compliance; MPEEP: moderate positive end-expiratory pressure; iPEEP: individualized positive end-expiratory pressure group

Three studies used driving pressure (DP) as a surrogate of respiratory compliance. These studies compared PEEP 5 mbar with individual PEEP (iPEEP) (n = 112): one of them in gynecology with esophageal-pressure guided PEEP; two of them in pelvic robotic surgery (one study used an esophageal pressure-guided setting of PEEP [34], while another - electrical impedance-guided PEEP [27, 35]. The meta-analysis of these studies showed a significant decrease in DP in iPEEP group (-4.13 (-2.63; -5.63) mbar, p < 0.001). We did not find significant variability in true effect between studies (T^2^ = 0.90; Chi^2^ 4.07, p = 0.13) may be due to low power. I^2^ was 51%, corresponding to a high proportion of true effect variation, and sampling error (Fig. 5). Estimation of the prediction interval of true effect also showed very wide distribution crossing zero line (Fig. S19). The risk of publication bias was low (Fig. S20).

### Mean arterial pressure and heart rate

13 studies (n = 974) measured MAP and HR. Meta-analysis of those studies revealed no significant differences in MAP (Fig. 6) or HR (Fig. 7) between any subgroup analysis. Also, these meta-analyses did not find significant variability in true effect in all subgroups, and a high proportion of the variation of the effects was possibly due to sampling error. Estimating the prediction interval of true effect also showed extensive distribution crossing zero line (Figs. S21-S28). The risk of publication bias in these data was low (Figs. S29-S36).

**Fig. 6:**
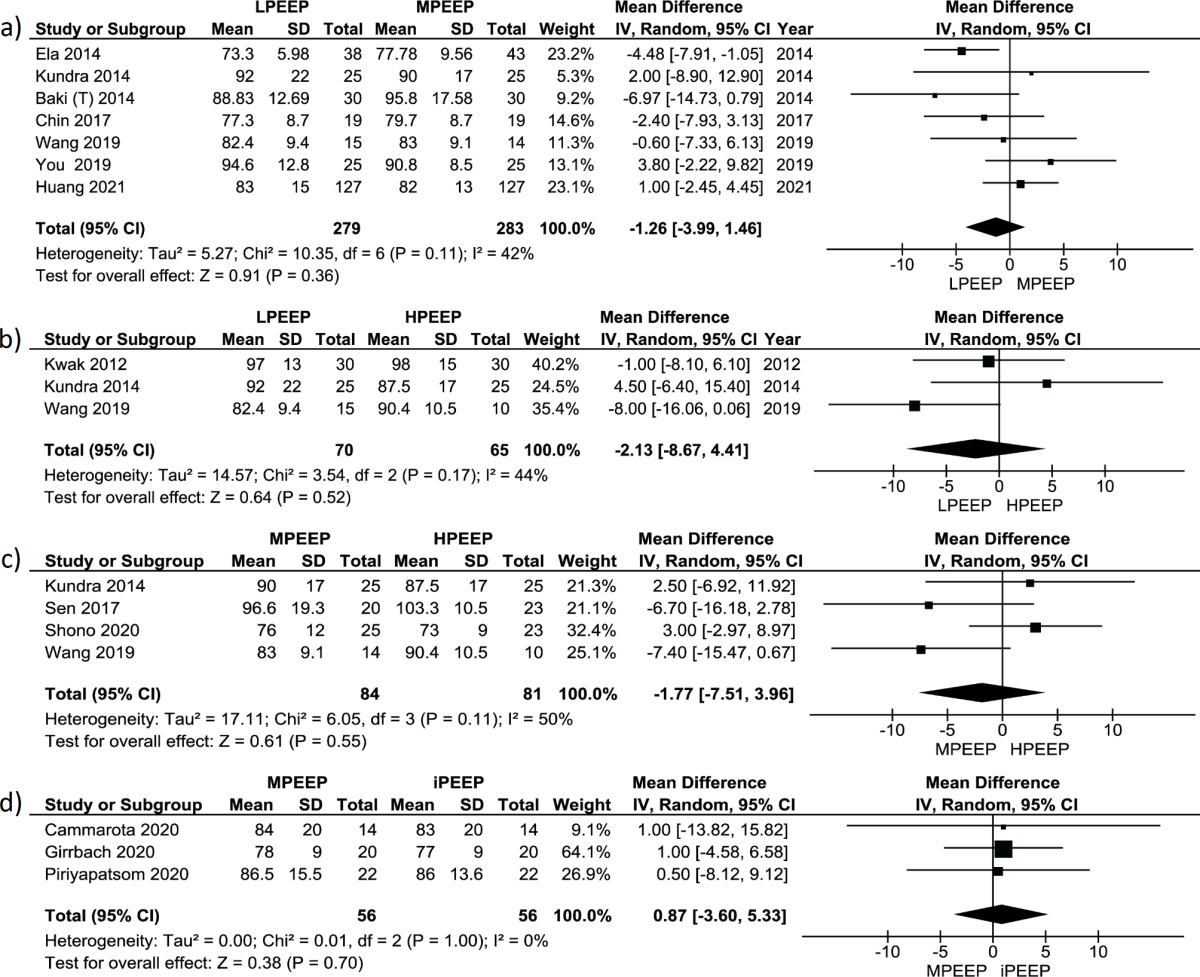
Forest plot for MAP comparing different PEEP strategy groups: (a) LPEEP vs. MPEEP; (b) LPEEP vs. HPEEP; (c) MPEEP vs. HPEEP; (d) MPEEP vs. iPEEP. Data are presented as mean differences and 95% confidence intervals. The vertical line represents no effect with the value of 0. The diamond represents the pooled mean effect estimate with 95% CI. It provides an overall measure of the difference in MAP values between different PEEP strategy groups. Abbreviations: CI: confidence interval; SD: standard deviation; I^2^: the ratio of excess dispersion to total dispersion; Тau^2^: the variance of the true effect sizes; Chi^2^: observed weighted sum of squares; df: degrees of freedom; MAP: mean arterial pressure; LPEEP: low positive end-expiratory pressure; MPEEP: moderate positive end-expiratory pressure; HPEEP: high positive end-expiratory pressure group; iPEEP: individualized positive end-expiratory pressure group

**Fig. 7:**
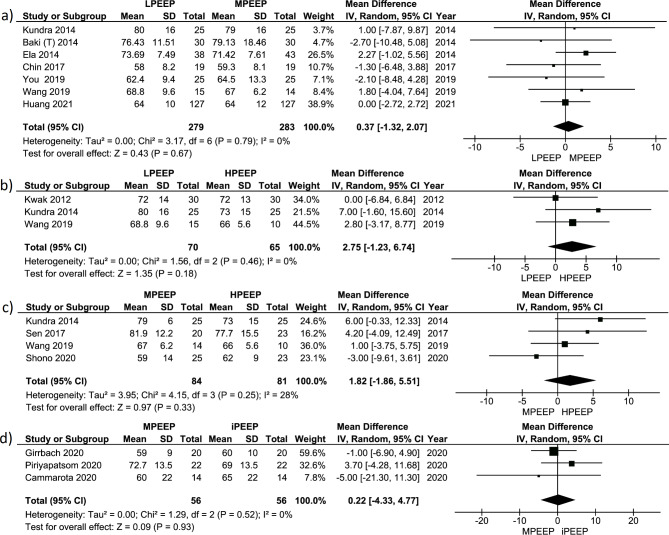
Forest plot for HR comparing different PEEP strategy groups: (a) LPEEP vs. MPEEP; (b) LPEEP vs. HPEEP; (c) MPEEP vs. HPEEP; (d) MPEEP vs. iPEEP. Data are presented as mean differences and 95% confidence intervals. The vertical line represents no effect with the value of 0. The diamond represents the pooled mean effect estimate with 95% CI. It provides an overall measure of the difference in HR values between different PEEP strategy groups. Abbreviations: CI: confidence interval; SD: standard deviation; I^2^: the ratio of excess dispersion to total dispersion; Тau^2^: the variance of the true effect sizes; Chi^2^: observed weighted sum of squares; df: degreeы of freedom; HR: heart rate; LPEEP: low positive end-expiratory pressure group; MPEEP: moderate positive end-expiratory pressure group; HPEEP: high positive end-expiratory pressure group; iPEEP: individualized positive end-expiratory pressure group

### Influence of the tidal volume and body position during surgery on study outcomes (For details see Supplement 1)

Meta-regression for the influence of tidal volume value did not find significant influence of tidal volume value on PaO_2_ or PaO_2_/FiO_2_ in all comparisons. For details see Supplement 1(Figures S37-S41). We found that when comparing LPEEP vs. HPEEP, patients in studies using tidal volume > 8 ml/kg may have lower dynamic compliance, but not in LPEEP vs. MPEEP comparison (Figures S42-S43). Also, in only one applicable comparison (LPEEP vs. MPEEP) higher tidal volume was associated with lower mean arterial pressure, but it did not affect the heart rate (Figures S44-S45).

Meta-regression for the influence of body position during surgery (Trendelenburg or Reverse Trendelenburg) did not find significant influence of body position on PaO_2_ or PaO_2_/FiO_2_ in all comparisons (see Supplement 1, **Figures S46-S50**). We found that when comparing LPEEP vs. HPEEP, patients in studies using Trendelenburg position may had lower dynamic compliance, but not in LPEEP vs. MPEEP comparison (Figures S51-S52). Influence of the body position on MAP and HR was not significant (Figures S53-S55).

## Discussion

Mechanical ventilation with muscle paralysis during anesthesia causes atelectasis, airway closure, and hypoxemia as a result [39]. These complications may lead to a higher risk of PPC [40]. The observational physiological study in anaesthetized and paralyzed patients without lung disease showed an increase in pleural pressure and a decrease in lung compliance in obese patients as compared to non-obese [41]. Laparoscopic surgery results in elevated intraabdominal pressure, which can lead to elevation of pleural pressure around 50% of intraabdominal pressure [42]. The use of higher PEEP during anesthesia with recruitment maneuvers could prevent development of atelectasis [43], improve respiratory compliance [44], and prevent atelectotrauma [45].

Patients who undergo laparoscopic surgery are at high risk of postoperative pulmonary complications, including atelectasis, pneumonia, and hypoxemia. Thus, a higher PEEP strategy in these patients can probably decrease the frequency or degree of these complications. This is the first systematic review and meta-analysis conducting association between different PEEP levels, oxygenation and respiratory mechanics during PNP in non-obese laparoscopic surgery patients without lung diseases.

Our meta-analysis included 21 RCTs with 1554 non-obese patients comparing different PEEP strategies in laparoscopic surgery patients. We divided PEEP groups as low (ZEEP), moderate (PEEP = 5 mbar), high PEEP (PEEP = 10 mbar), and individual PEEP (around 10–12 mbar after an increase in abdominal pressure).

First, we focused our attention on arterial oxygenation as a marker of alveolar collapse. We found heterogeneity in the estimation of the arterial oxygenation - smaller proportion of studies used PaO_2_/FiO_2_, but vast majority of them used PaO_2_ instead, that made the interpretation less relevant. In most comparisons higher PEEP significantly increased PaO_2_ or PaO_2_/FiO_2_, and we did not find variation in true effect (due to low heterogeneity or low power). Although the mean effect on PaO_2_/FiO_2_ and its confidence interval was statistically significant after the comparison of low vs. high PEEP, or moderate vs. high PEEP, the predictive interval for true effects for low vs. high PEEP had wide range crossing reference line. We observed the same picture in studies comparing only PaO_2_. High PEEP level group showed significantly higher PaO_2_ as compared to low PEEP group using mean effect and it’s CI, but the wide variance in the predictive interval, crossing the reference line. Of note, these studies showed low Chi^2^, I^2^ and T^2^ that means high variation of sampling error but not true effects. So, low level of PEEP (ZEEP) as compared to moderate, or high PEEP during PNP was associated with worse oxygenation. Comparison of moderate vs. high PEEP, and moderate vs. individualized PEEP (in robotic surgery) showed better oxygenation in HPEEP and iPEEP groups with insignificant variation of true effects between groups (maybe due to low power) but wide range of dispersion of true effect size.

Second. Comparison of LPEEP vs. MPEEP reveals high variability of the true effect and predictive interval of Cdyn, in the opposite, HPEEP vs. LPEEP showed a significant increase in Cdyn with high variation of true effect size. We can speculate that PEEP = 5 mbar could be insufficient to increase lung compliance as compared to ZEEP, but HPEEP could improve lung aeration. We think that meta-analysis of MPEEP vs. HPEEP was underpowered to detect any changes in Cdyn. But similar meta-analysis compared MPEEP vs. iPEEP in pelvic surgery, that is close to HPEEP value, revealed a significant decrease in DP, corresponding to the increase of the respiratory compliance, but also high true effect variation.

A decrease in respiratory compliance, as well as an increase in its inverse value (known as driving pressure), may reflect overdistension (strain) of the lungs during mechanical ventilation [46]. Lung overdistension due to high PEEP or high tidal volume may damage lung tissue and impair pulmonary microcirculation [47]. In a secondary analysis of RCTs elevated driving pressure was found as a predictor of mortality in ARDS patients [48]. In a large retrospective study higher mechanical power due to higher DP led to a greater risk of postoperative respiratory failure requiring intubation in elective surgical patients under general anesthesia [49]. Our meta-analysis and meta-regression for the influence of the tidal volume on study outcomes have found important results that not HPEEP per se, but combination of HPEEP with tidal volume above 8 mL/kg may decrease compliance and MAP. Moreover, the meta-analysis found that iPEEP (generally higher than HPEEP) as compared to LPEEP during PNP reduced DP and did not affect MAP in all studies without significant variability of true effect, i.e. did not lead to overdistension despite of high values of PEEP.

Third. There were no significant differences in hemodynamic parameters between groups in meta-analyses. However, the probability of sampling error in hemodynamic parameters was high, and predictive intervals were wide, so the results should be interpreted with caution. Though we did not find the influence of PEEP strategy on hemodynamics, meta-regression analysis revealed that tidal volume > 8 mL/kg may affect MAP.

Forth. Our study revealed heterogeneity between studies concerning tidal volume and body position. Clinical implications of meta-regression can be summarized as follows: combination of MPEEP or HPEEP with tidal volumes more than 8 mL/kg may decrease respiratory compliance and decrease MAP; in the opposite, low tidal volume strategy in these PEEP strategies may be safe in relation to respiratory mechanics and hemodynamics; also, combination of Trendelenburg position with HPEEP may decrease respiratory compliance.

Most of the recent meta-analyses concerning PEEP levels in abdominal surgery focused on PPCs, hypoxemia, and hypotension. A recent meta-analysis including 63 trials in non-cardiac surgery found that lung-protective ventilation (low tidal volume with PEEP) results in a decrease in pulmonary complications, but failed to find a beneficial effect of higher PEEP as compared to lower PEEP (around 5 mbar) on PPC [50]. Meta-analysis of three large multicenter RCTs (PROVHILO, iPROVE and PROBESE) compared low versus high PEEP in non-cardiothoracic and non-neurological surgery showed fewer episodes of desaturation but more frequent intraoperative hypotension, and no effect in PPCs in the higher PEEP group [51]. Of note, included trials had several limitations. For example, PROVHILO trial excluded patient with obesity and laparoscopic surgery [52], PROBESE trial compared fixed level of PEEP 12 mbar (versus PEEP 4 mbar in control group) in patients with BMI > 40 kg/m^2^ who underwent predominantly laparoscopic abdominal surgery [53]. In spite of abovementioned limitations, in this meta-analysis in subgroup of laparoscopic surgery PPCs were significantly lower. Recent meta-analyses comparing individualised PEEP with other strategies in abdominal surgery showed better oxygenation, higher respiratory compliance, and less PPCs in the individualized PEEP groups [14, 54]. Individualized PEEP could have impact on PPCs in one-lung ventilation. These results correspond to our meta-analysis that failed to show superiority of HPEEP over MPEEP. Recent meta-analysis of 8 trials in thoracic surgery patients showed that individual PEEP during one-lung ventilation was associated with fewer postoperative pulmonary complications and better perioperative oxygenation [55].

Our study includes important limitations. First, it concerns heterogeneity of surgical site and duration of the operation, so some clinically important subgroups remained small. Second, included studies used heterogeneous measures of oxygenation, and respiratory compliance as well as different PEEP levels, body positions and tidal volumes. Third, some meta-analyses could be underpowered and had high risk of publication bias. Forth, we did not focus on PPCs. Lastly, according to heterogeneity, a network meta-analysis design would have been more appropriate.

We can mention some study strengths. We selected only non-obese patients with PNP, eliminating the effect of obesity on oxygenation and respiratory compliance. We used predictive intervals to show a real variation of the true effects. Also, we performed meta-regression to separate the influence of tidal volume and body position from PEEP strategies.

## Conclusions

HPEEP and iPEEP during PNP in non-obese patients could promote oxygenation and increase Cdyn without clinically significant changes in MAP and HR. MPEEP could be insufficient to increase respiratory compliance and improve oxygenation. LPEEP may lead to decreased respiratory compliance and worsened oxygenation.

### Electronic supplementary material

Below is the link to the electronic supplementary material.

Supplementary Material 1

Supplementary Material 2

## Data Availability

The datasets used and/or analyzed during the current study are available from the corresponding author upon reasonable request.

## Abbreviations

ARDS: Acute respiratory distress syndrome
BMI: Body mass index
Cdyn: Dynamic compliance
CI: Confidence intervals
DP: Driving pressure
EELV: End–expiratory volume lung volume
FiO_2_: Fraction of inspired oxygen
IAP: Intra–abdominal pressure
ICU: Intensive care unit
LPV: Lung protective ventilation
MAP: Mean arterial pressure
SMD: Standardized mean difference
PaO_2_: Arterial partial pressure of oxygen
PEEP: Positive end–expiratory pressure
Pes: Esophageal pressure
PNP: Pneumoperitoneum
PPC: Postoperative pulmonary complications
RCT: Randomized control study
RM: Recruitment maneuver
RR: Risk ratio
TV: Tidal volume
ZEEP: Zero end–expiratory pressure
