# Supplementary material for: Impact of a positive end-expiratory pressure strategy on oxygenation, respiratory compliance, and hemodynamics during laparoscopic surgery in non-obese patients: a systematic review and meta-analysis of randomized controlled trials
Source: BMC Anesthesiol. 2023 Nov 11;23:371. doi: 10.1186/s12871-023-02337-0 (PMC10638810; doi:10.1186/s12871-023-02337-0)
Supplement: Supplementary file 1 — Supplementary Material 1 [file 12871_2023_2337_MOESM1_ESM.docx]

**Online Data Supplement 1**

**Oxygenation (PaO_2_)**

Meta-analysis of 8 studies showed that MPEEP strategy led to a significant increase in PaO_2_ as compared to LPEEP strategy (all studies used Zero End-Expiratory Pressure - ZEEP) (+19.64 (11.05; 28.22) mmHg, p<0.001). We did not find variations in true effect between studies (Chi^2^ 6.04, p= 0.53). Due to Chi^2^ being less than df, the variation of true effect size (T^2^) is zero. Almost all dispersion of PaO_2_ between LEEP and MEEP can be attributed to sampling error (I^2^=0%) (**Fig. S****1a**). We did not find evidence that the true effect of MPEEP vs LPEEP on PaO_2_ varies, maybe due to real consistency of the true effect between studies, or due to low power. This data (I^2^=0% and T^2^ = 0.00) can mask the presence of real heterogeneity. We suppose that these data showed that almost all dispersion could be attributed to random error. Estimation of the PI of true effect showed wide distribution crossing zero line (**Fig. S1**). We found that predictive interval PI on PaO_2_ depending on LEEP or MPEEP crossed zero line that would be the consequence of different types of surgery or body mass indices as a risk factor for intraoperative atelectasis. The funnel plot for these studies showed a low risk of publication bias (**Fig. S2**).

Meta-analysis of LPEEP (all studies used ZEEP) vs HPEEP (all studies used 10 mbar) studies showed similar results with a significant increase in PaO_2_ as compared to LPEEP strategy (+29.38 (16.20; 42.56) mmHg, p<0.0001)(**Fig. 3b**). Data on heterogeneity analysis revealed similar results as in LPEEP vs MPEEP meta-analysis. However, the predictive interval for true effect is more expansive than in meta-analysis of LPEEP vs MPEEP studies, possibly, due to the small sample size (**Fig. S3**). Also, the funnel plot showed a low risk of publication bias (**Fig. S4**).

Meta-analysis of MPEEP (all studies used 5 mbar) vs HPEEP (all studies used 10 mbar) studies showed a significant increase in PaO_2_ in HPEEP group as compared to MPEEP strategy (+22.00 (1.11; 42.88) mmHg, p=0.04) (**Fig. 3c**). But data showed significant heterogeneity (Chi^2^ 6.42, p=0.09) of the true effect. Moreover, the distribution of true effect size was wide (T^2^ = 221.01), and I^2^ 53%, which can correspond to a high real proportion of true effect variation, not only by sampling error. The predictive interval for true effect was wider than in meta-analysis of LPEEP vs HPEEP studies (**Fig. S5**). The risk of publication bias was low (**Fig. S6**).
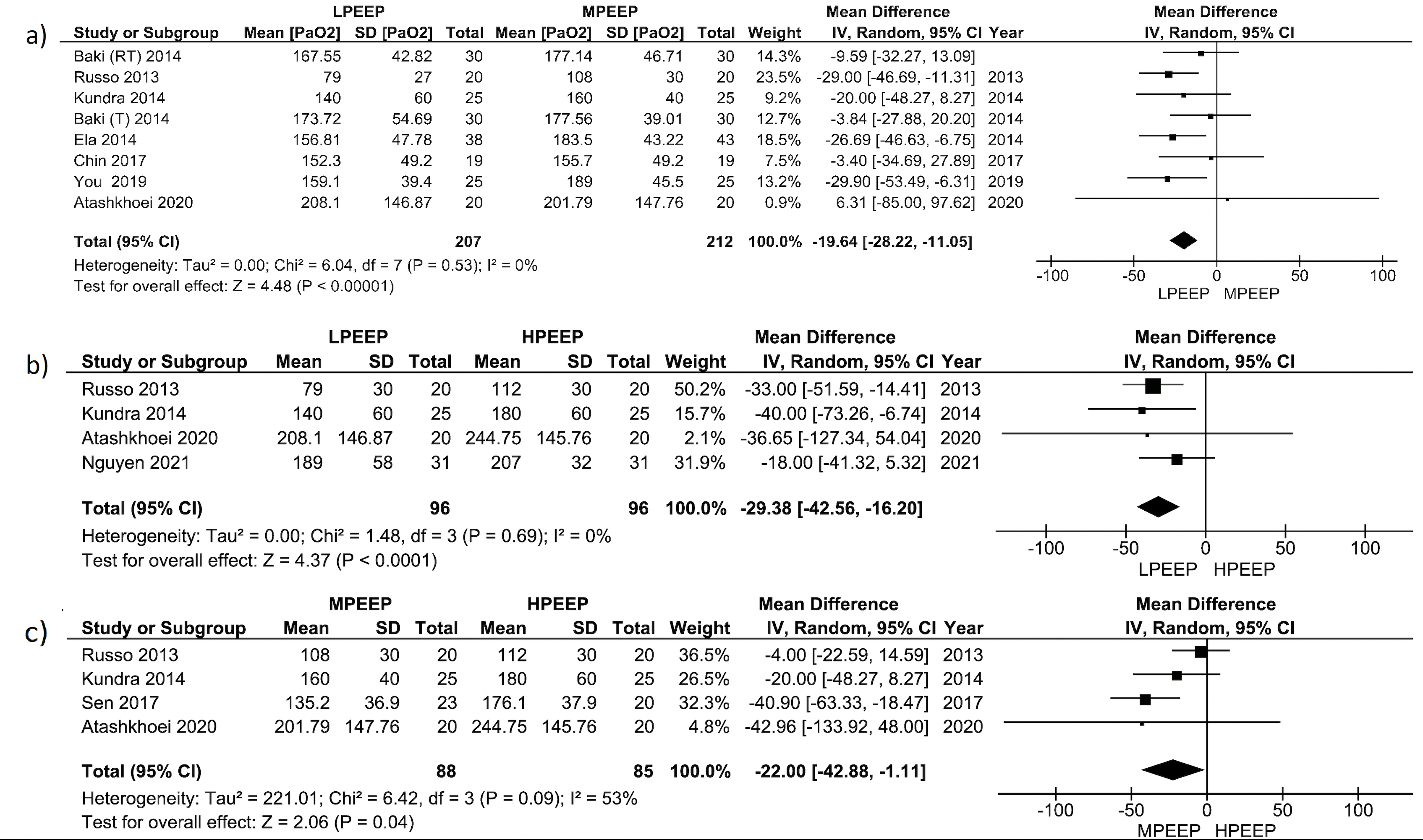


**Figure S1.** **Forest plot for PaO_2_ comparing patients in different PEEP strategy groups: a) LPEEP vs MPEEP; b) LPEEP vs HPEEP; c) MPEEP vs HPEEP.**

Data are presented as mean differences and 95% confidence intervals. The vertical line represents no effect with the value of 0. The diamond represents the pooled mean effect estimate with 95% CI. It provides an overall measure of the difference in PaO_2_ values between different PEEP strategy groups.

**Abbreviations**: CI: confidence interval; SD: standard deviation; I^2^: the ratio of excess dispersion to total dispersion; Тau^2^: the variance of the true effect sizes; Chi^2^: observed weighted sum of squares; df: degrees of freedom; PaO_2_: arterial partial pressure of oxygen; LPEEP: low positive end-expiratory pressure group; MPEEP: moderate positive end-expiratory pressure group; HPEEP: high positive end-expiratory pressure group.


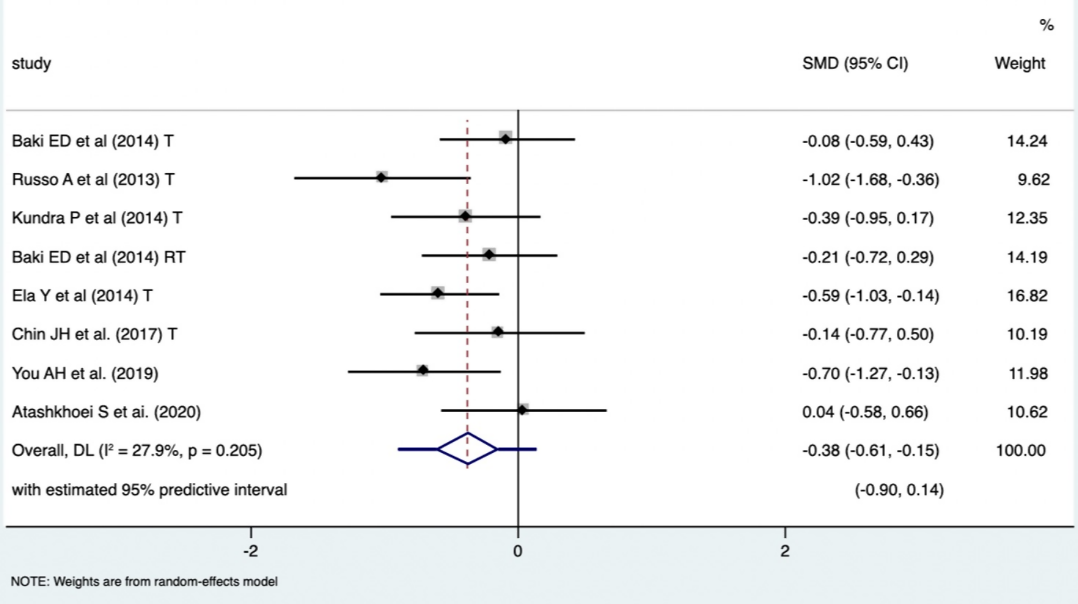


**Figure S2. Forest plot for estimation of the predictive interval of true effect on PaO_2_ for LPEEP vs MPEEP.**

Data are presented as standardized mean difference and 95% confidence intervals and 95% predictive intervals. The diamond symbolizes the summary estimate, which represents the combined effect size of all included studies. It provides an overall measure of the difference in PaO2 outcomes between low PEEP and moderate PEEP.

**Abbreviations**: I^2^: the ratio of excess dispersion to total dispersion; SMD: standardized mean difference; CI: confidence interval; PaO2: arterial partial pressure of oxygen; LPEEP – low positive end-expiratory pressure; MPEEP – moderate positive end-expiratory pressure.


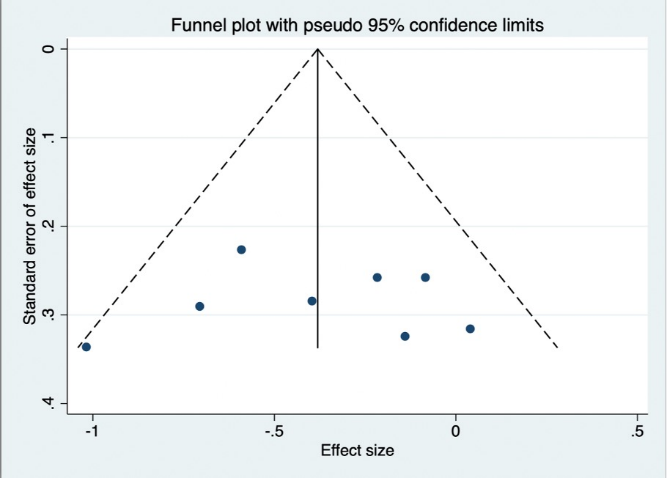


**Figure S3. Funnel plot of publication bias in** **PaO_2_ for LPEEP vs MPEEP**

А funnel plot of effect size and standard error of effect size in PaO2 for LPEEP vs MPEEP. The triangular lines on both sides of the summary estimate represent the precision measures, such as pseudo 95 % confidence intervals. The middle solid line indicates the overall effect of the meta-analysis. Each dot represents the individual study.

**Abbreviations**: PaO2: arterial partial pressure of oxygen; LPEEP – low positive end-expiratory pressure; MPEEP – moderate positive end-expiratory pressure.


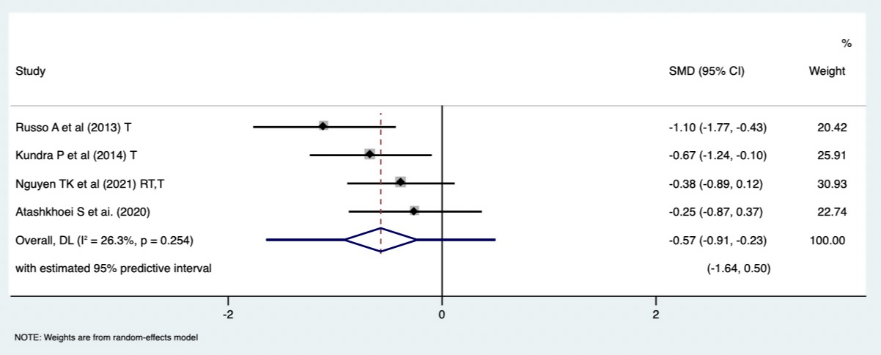


**Figure S4. Forest plot for estimation of the predictive interval of true effect on PaO_2_ for LPEEP vs HPEEP.**

Data are presented as standardized mean difference and 95% confidence intervals and 95% predictive intervals. The diamond symbolizes the summary estimate, which represents the combined effect size of all included studies. It provides an overall measure of the difference in PaO2 outcomes between low PEEP and high PEEP.

**Abbreviations**: I^2^: the ratio of excess dispersion to total dispersion; SMD: standardized mean difference; CI: confidence interval; PaO2: arterial partial pressure of oxygen; LPEEP – low positive end-expiratory pressure; HPEEP – high positive end-expiratory pressure.


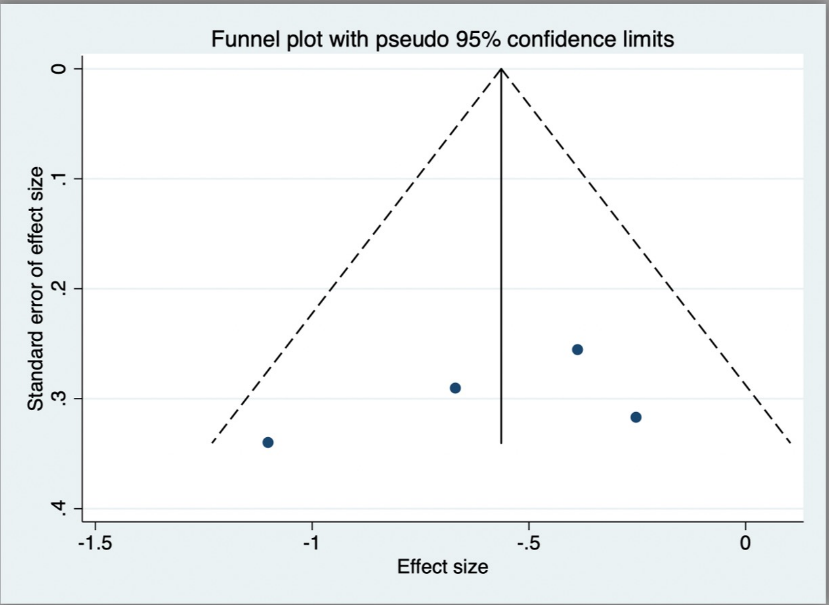


**Figure S5. Funnel plot of publication bias in PaO_2_ for LPEEP vs HPEEP**

А funnel plot of effect size and standard error of effect size in PaO2 for LPEEP vs HPEEP. The triangular lines on both sides of the summary estimate represent the precision measures, such as pseudo 95 % confidence intervals. The middle solid line indicates the overall effect of the meta-analysis. Each dot represents the individual study.

**Abbreviations**: PaO2: arterial partial pressure of oxygen; LPEEP – low positive end-expiratory pressure; HPEEP – high positive end-expiratory pressure.


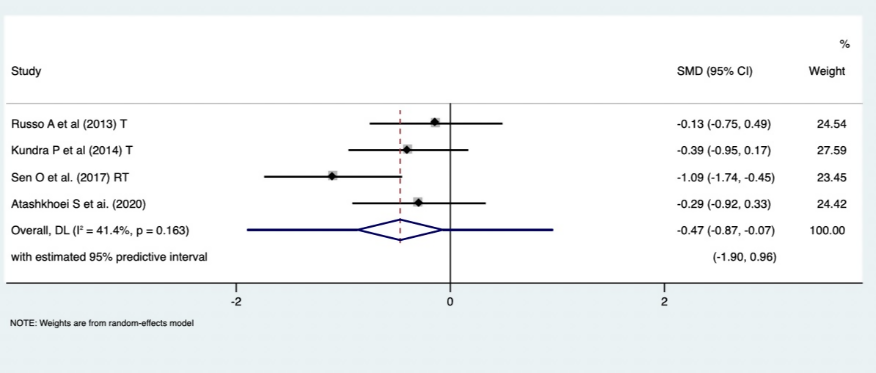


Figure 5 supplementary

**Figure S6. Forest plot for estimation of the predictive interval of true effect on PaO_2_ for MPEEP vs HPEEP.**

Data are presented as standardized mean difference and 95% confidence intervals and 95% predictive intervals. The diamond symbolises the summary estimate, which represents the combined effect size of all included studies. It provides an overall measure of the difference in PaO2 outcomes between moderate PEEP and high PEEP.

**Abbreviations**: I^2^: the ratio of excess dispersion to total dispersion; SMD: standardized mean difference; CI: confidence interval; PaO2: arterial partial pressure of oxygen; MPEEP – moderate positive end-expiratory pressure; HPEEP – high positive end-expiratory pressure.


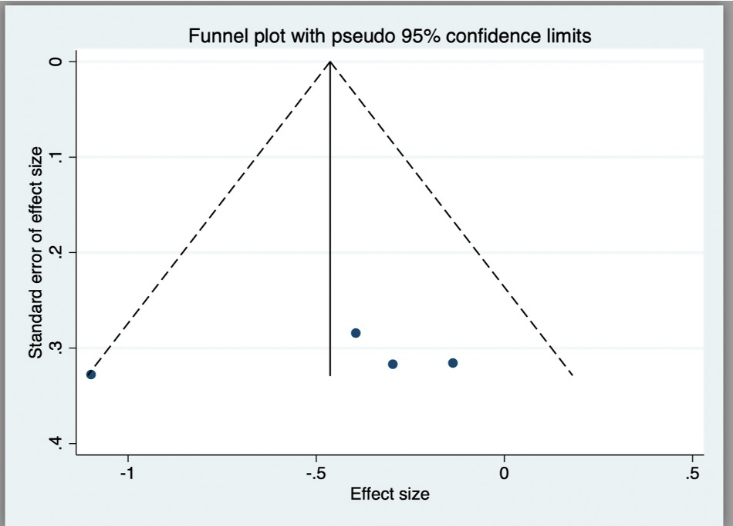


**Figure S7. Funnel plot of publication bias in PaO_2_ for MPEEP vs HPEEP**

А funnel plot of effect size and standard error of effect size in PaO2 for MPEEP vs HPEEP. The triangular lines on both sides of the summary estimate represent the precision measures, such as pseudo 95 % confidence intervals. The middle solid line indicates the overall effect of the meta-analysis. Each dot represents the individual study.

**Abbreviations**: PaO2: arterial partial pressure of oxygen; MPEEP – moderate positive end-expiratory pressure; HPEEP – high positive end-expiratory pressure.


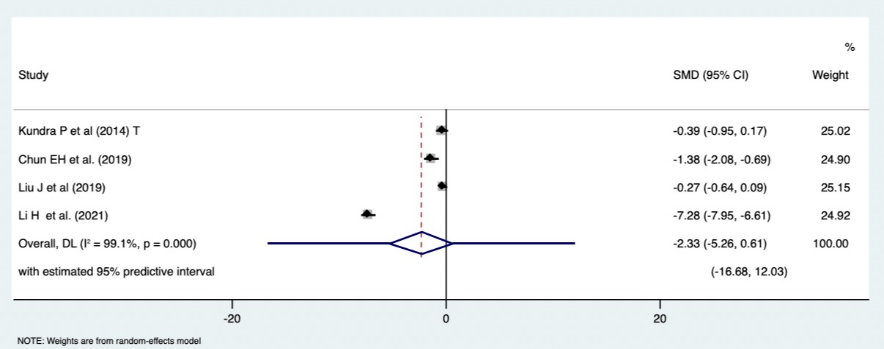


**Figure S8. Forest plot for estimation of the predictive interval of true effect on PaO_2_/FiO_2_ for LPEEP vs MPEEP.**

Data are presented as standardized mean difference and 95% confidence intervals and 95% predictive intervals. The diamond symbolizes the summary estimate, which represents the combined effect size of all included studies. It provides an overall measure of the difference in PaO2/FiO2 outcomes between low PEEP and moderate PEEP.

**Abbreviations**: I^2^: the ratio of excess dispersion to total dispersion; SMD: standardized mean difference; CI: confidence interval; PaO2/FiO2: arterial oxygen partial pressure to fractional inspired oxygen ratio; LPEEP – low positive end-expiratory pressure; MPEEP – moderate positive end-expiratory pressure.


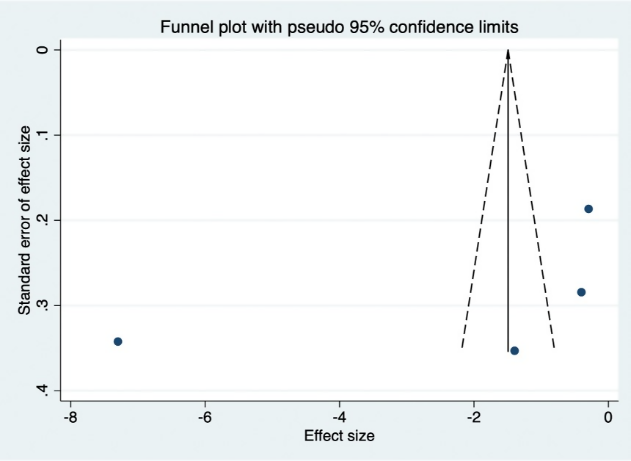


**Figure S9. Funnel plot of publication bias in PaO2/FiO2 for LPEEP vs MPEEP**

А funnel plot of effect size and standard error of effect size in PaO2/FiO2 for LPEEP vs MPEEP. The triangular lines on both sides of the summary estimate represent the precision measures, such as pseudo 95 % confidence intervals. The middle solid line indicates the overall effect of the meta-analysis. Each dot represents the individual study.

**Abbreviations**: PaO2/FiO2: arterial oxygen partial pressure to fractional inspired oxygen ratio; LPEEP – low positive end-expiratory pressure; MPEEP – moderate positive end-expiratory pressure.


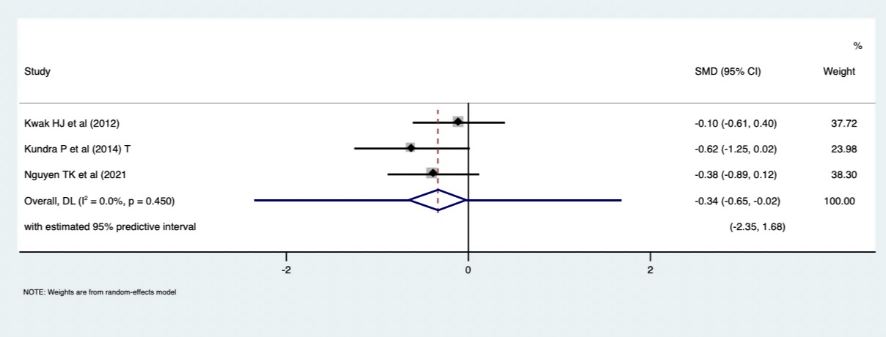


**Figure S10. Forest plot for estimation of the predictive interval of true effect on PaO2/FiO2 for LPEEP vs HPEEP.**

Data are presented as standardized mean difference and 95% confidence intervals and 95% predictive intervals. The diamond symbolizes the summary estimate, which represents the combined effect size of all included studies. It provides an overall measure of the difference in PaO2/FiO2 outcomes between low PEEP and high PEEP.

**Abbreviations**: I^2^: the ratio of excess dispersion to total dispersion; SMD: standardized mean difference; CI: confidence interval; PaO2/FiO2: arterial oxygen partial pressure to fractional inspired oxygen ratio; LPEEP – low positive end-expiratory pressure; HPEEP – high positive end-expiratory pressure.


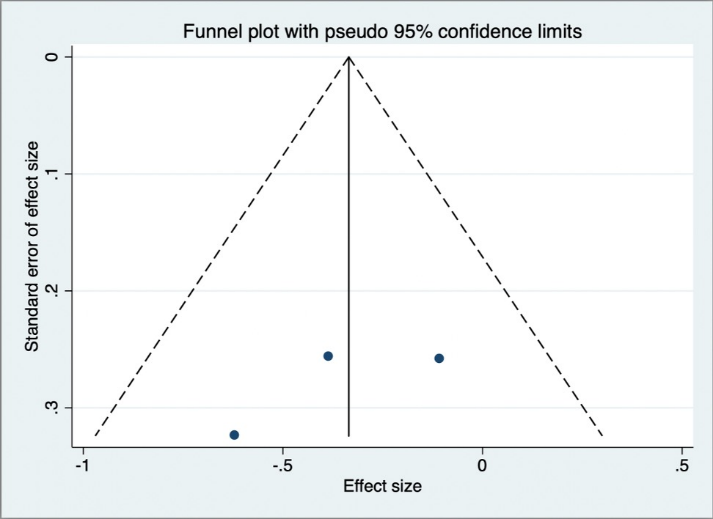


**Figure S11. Funnel plot of publication bias in PaO2/FiO2 for LPEEP vs HPEEP**

А funnel plot of effect size and standard error of effect size in PaO2/FiO2 for LPEEP vs HPEEP. The triangular lines on both sides of the summary estimate represent the precision measures, such as pseudo 95 % confidence intervals. The middle solid line indicates the overall effect of the meta-analysis. Each dot represents the individual study.

**Abbreviations**: PaO2/FiO2: arterial oxygen partial pressure to fractional inspired oxygen ratio; LPEEP – low positive end-expiratory pressure; HPEEP – high positive end-expiratory pressure.


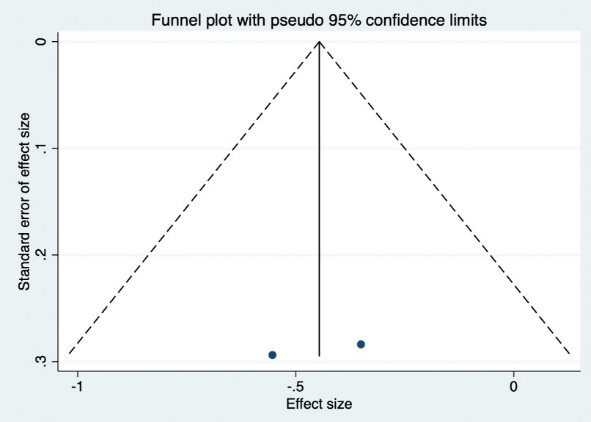


**Figure S12. Funnel plot of publication bias in PaO2/FiO2 for MPEEP vs HPEEP**

А funnel plot of effect size and standard error of effect size in PaO2/FiO2 for MPEEP vs HPEEP. The triangular lines on both sides of the summary estimate represent the precision measures, such as pseudo 95 % confidence intervals. The middle solid line indicates the overall effect of the meta-analysis. Each dot represents the individual study.

**Abbreviations**: PaO2/FiO2: arterial oxygen partial pressure to fractional inspired oxygen ratio; MPEEP – moderate positive end-expiratory pressure; HPEEP – high positive end-expiratory pressure.


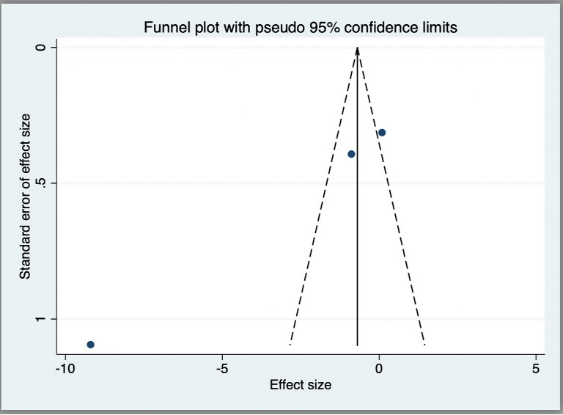


**Figure S13. Funnel plot of publication bias in PaO2/FiO2 for MPEEP vs iPEEP**

А funnel plot of effect size and standard error of effect size in PaO2/FiO2 for MPEEP vs iPEEP. The triangular lines on both sides of the summary estimate represent the precision measures, such as pseudo 95 % confidence intervals. The middle solid line indicates the overall effect of the meta-analysis. Each dot represents the individual study.

**Abbreviations**: PaO2/FiO2: arterial oxygen partial pressure to fractional inspired oxygen ratio; MPEEP – moderate positive end-expiratory pressure; iPEEP – individualised positive end-expiratory pressure.


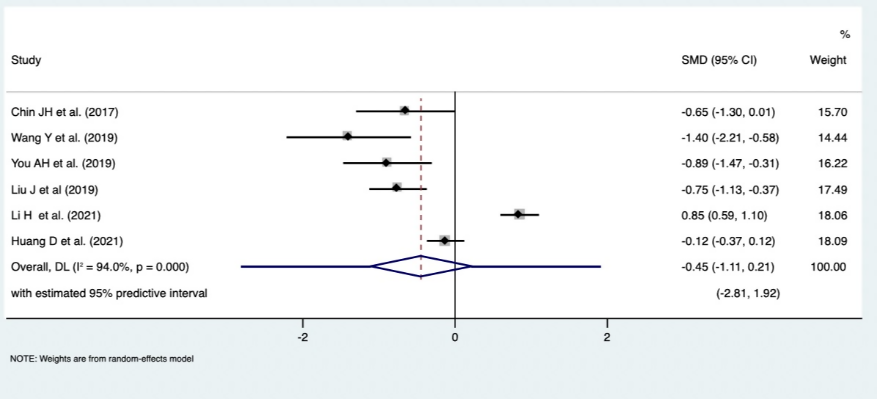


**Figure S14. Forest plot for estimation of the predictive interval of true effect on Cdyn for LPEEP vs MPEEP.**

Data are presented as standardized mean difference and 95% confidence intervals and 95% predictive intervals. The diamond symbolizes the summary estimate, which represents the combined effect size of all included studies. It provides an overall measure of the difference in Cdyn outcomes between low PEEP and moderate PEEP.

**Abbreviations**: I^2^: the ratio of excess dispersion to total dispersion; SMD: standardized mean difference; CI: confidence interval; Cdyn: dynamic compliance; LPEEP – low positive end-expiratory pressure; MPEEP – moderate positive end-expiratory pressure.


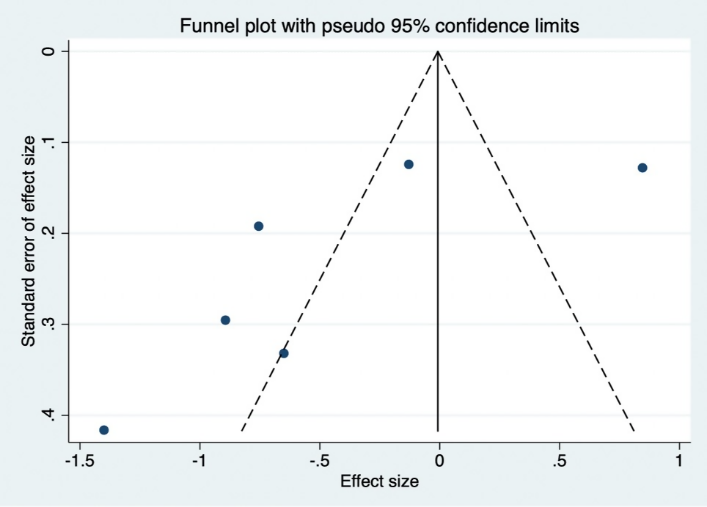


**Figure S15. Funnel plot of publication bias in Cdyn for LPEEP vs MPEEP**

А funnel plot of effect size and standard error of effect size in Cdyn for LPEEP vs MPEEP. The triangular lines on both sides of the summary estimate represent the precision measures, such as pseudo 95 % confidence intervals. The middle solid line indicates the overall effect of the meta-analysis. Each dot represents the individual study.

**Abbreviations**: Cdyn: dynamic compliance; LPEEP – low positive end-expiratory pressure; MPEEP – moderate positive end-expiratory pressure.


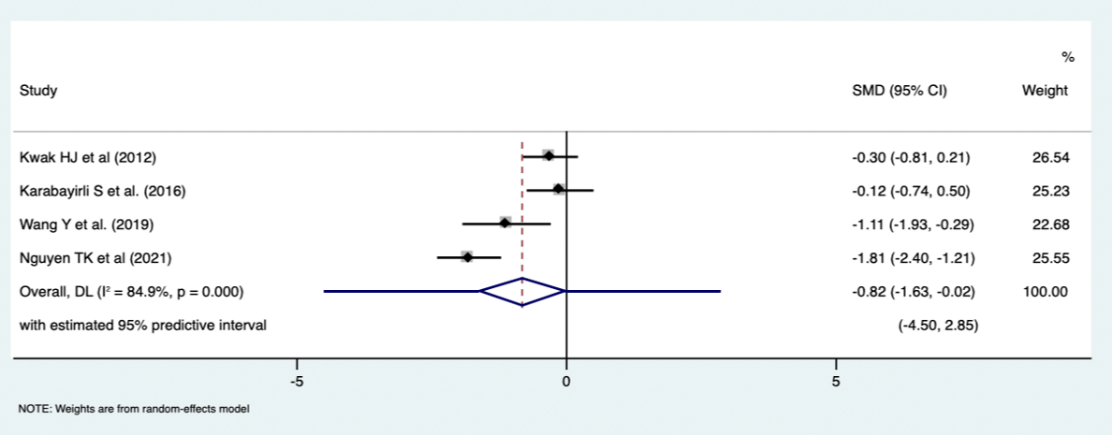


**Figure S16. Forest plot for estimation of the predictive interval of true effect on Cdyn for LPEEP vs HPEEP.**

Data are presented as standardized mean difference and 95% confidence intervals and 95% predictive intervals. The diamond symbolizes the summary estimate, which represents the combined effect size of all included studies. It provides an overall measure of the difference in Cdyn outcomes between low PEEP and high PEEP.

**Abbreviations**: I^2^: the ratio of excess dispersion to total dispersion; SMD: standardized mean difference; CI: confidence interval; Cdyn: dynamic compliance; LPEEP – low positive end-expiratory pressure; HPEEP – high positive end-expiratory pressure.


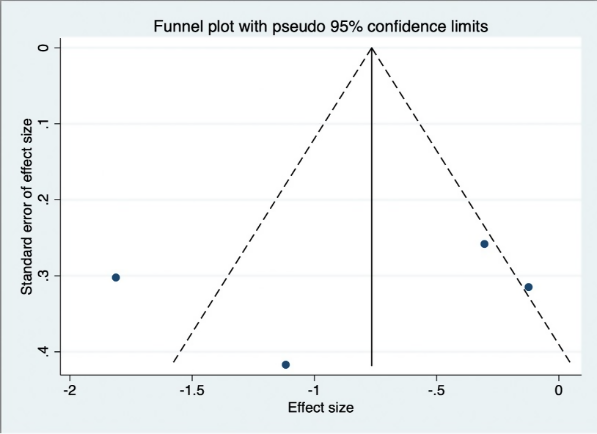


**Figure S17. Funnel plot of publication bias in Cdyn for LPEEP vs HPEEP**

А funnel plot of effect size and standard error of effect size in Cdyn for LPEEP vs HPEEP. The triangular lines on both sides of the summary estimate represent the precision measures, such as pseudo 95 % confidence intervals. The middle solid line indicates the overall effect of the meta-analysis. Each dot represents the individual study.

**Abbreviations**: Cdyn: dynamic compliance; LPEEP – low positive end-expiratory pressure; HPEEP – high positive end-expiratory pressure.


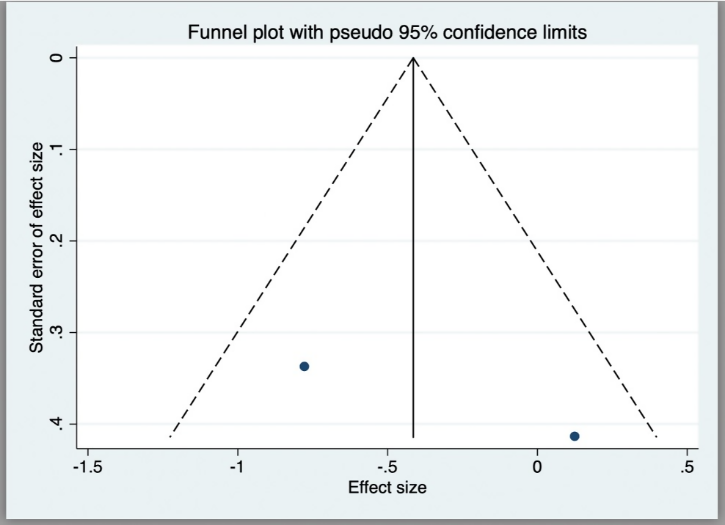


**Figure S18. Funnel plot of publication bias in Cdyn for MPEEP vs HPEEP**

А funnel plot of effect size and standard error of effect size in Cdyn for MPEEP vs HPEEP. The triangular lines on both sides of the summary estimate represent the precision measures, such as pseudo 95 % confidence intervals. The middle solid line indicates the overall effect of the meta-analysis. Each dot represents the individual study.

**Abbreviations**: Cdyn: dynamic compliance; MPEEP – moderate positive end-expiratory pressure; HPEEP – high positive end-expiratory pressure.


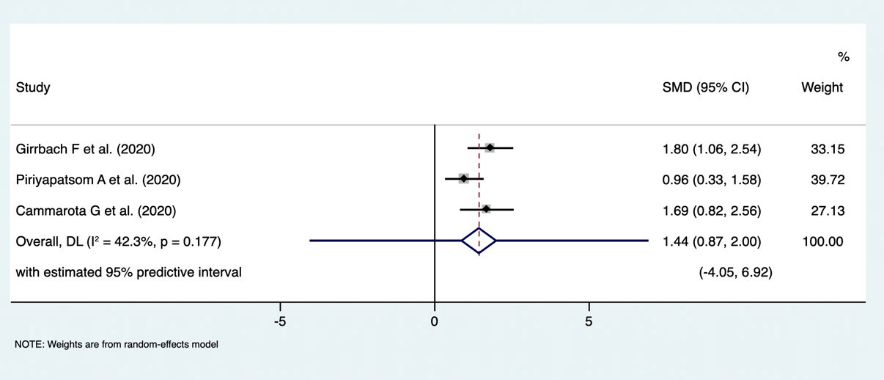


**Figure S19. Forest plot for estimation of the predictive interval of true effect on DP (as a surrogate of Cdyn) for MPEEP vs iPEEP.**

Data are presented as standardized mean difference and 95% confidence intervals and 95% predictive intervals. The diamond symbolizes the summary estimate, which represents the combined effect size of all included studies. It provides an overall measure of the difference in DP outcomes between moderate PEEP and individualized PEEP.

**Abbreviations**: I^2^: the ratio of excess dispersion to total dispersion; SMD: standardized mean difference; CI: confidence interval; Cdyn: dynamic compliance; DP: driving pressure; MPEEP – moderate positive end-expiratory pressure; iPEEP – individualised positive end-expiratory pressure.


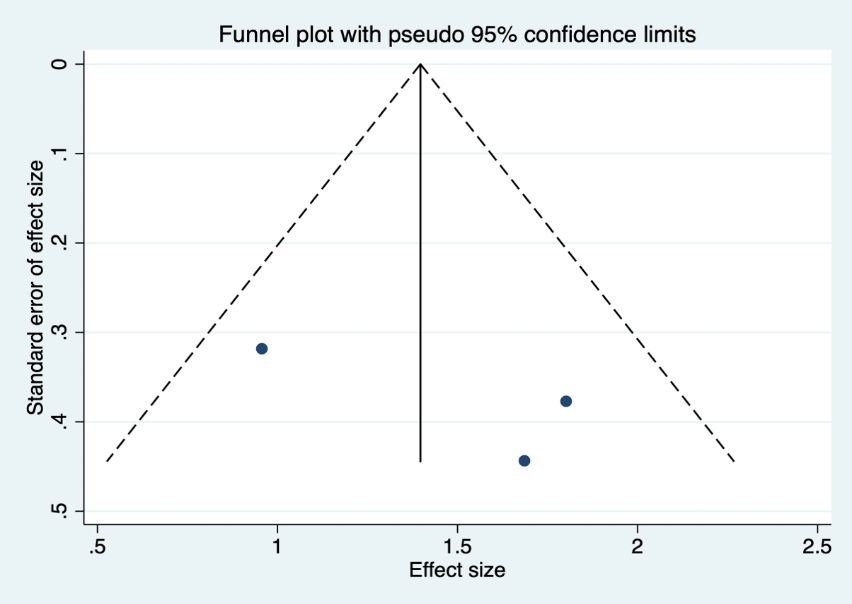


**Figure S20. Funnel plot of publication bias in DP (as a surrogate of Cdyn) for MPEEP vs iPEEP**

А funnel plot of effect size and standard error of effect size in DP for MPEEP vs iPEEP. The triangular lines on both sides of the summary estimate represent the precision measures, such as pseudo 95 % confidence intervals. The middle solid line indicates the overall effect of the meta-analysis. Each dot represents the individual study.

**Abbreviations**: Cdyn: dynamic compliance; DP: driving pressure; MPEEP – moderate positive end-expiratory pressure; iPEEP – individualised positive end-expiratory pressure.


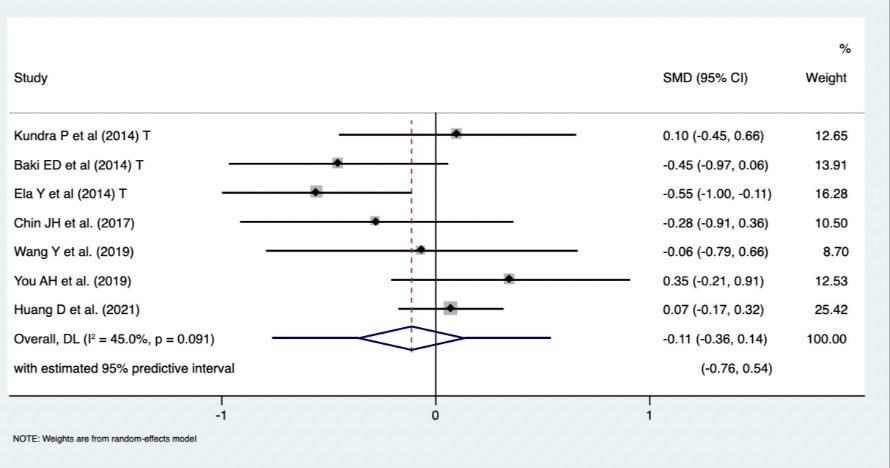


**Figure S21. Forest plot for estimation of the predictive interval of true effect on MAP for LPEEP vs MPEEP.**

Data are presented as standardized mean difference and 95% confidence intervals and 95% predictive intervals. The diamond symbolizes the summary estimate, which represents the combined effect size of all included studies. It provides an overall measure of the difference in MAP outcomes between low PEEP and moderate PEEP.

**Abbreviations**: I^2^: the ratio of excess dispersion to total dispersion; SMD: standardized mean difference; CI: confidence interval; MAP: mean arterial pressure; LPEEP – low positive end-expiratory pressure; MPEEP – moderate positive end-expiratory pressure.


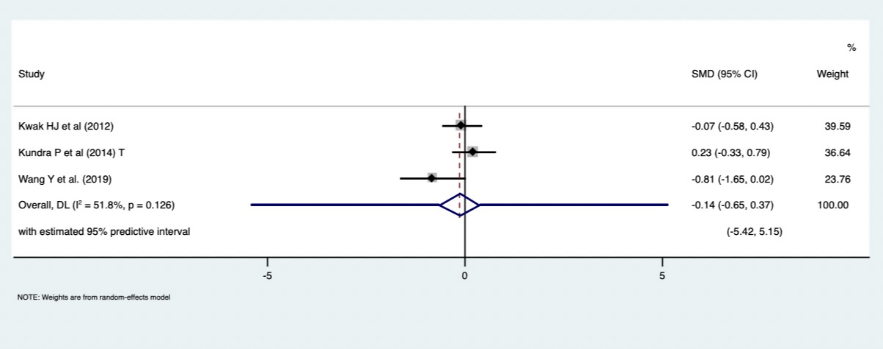


**Figure S22. Forest plot for estimation of the predictive interval of true effect on MAP for LPEEP vs HPEEP.**

Data are presented as standardized mean difference and 95% confidence intervals and 95% predictive intervals. The diamond symbolizes the summary estimate, which represents the combined effect size of all included studies. It provides an overall measure of the difference in MAP outcomes between low PEEP and high PEEP.

**Abbreviations**: I^2^: the ratio of excess dispersion to total dispersion; SMD: standardized mean difference; CI: confidence interval; MAP: mean arterial pressure; LPEEP – low positive end-expiratory pressure; HPEEP – high positive end-expiratory pressure.


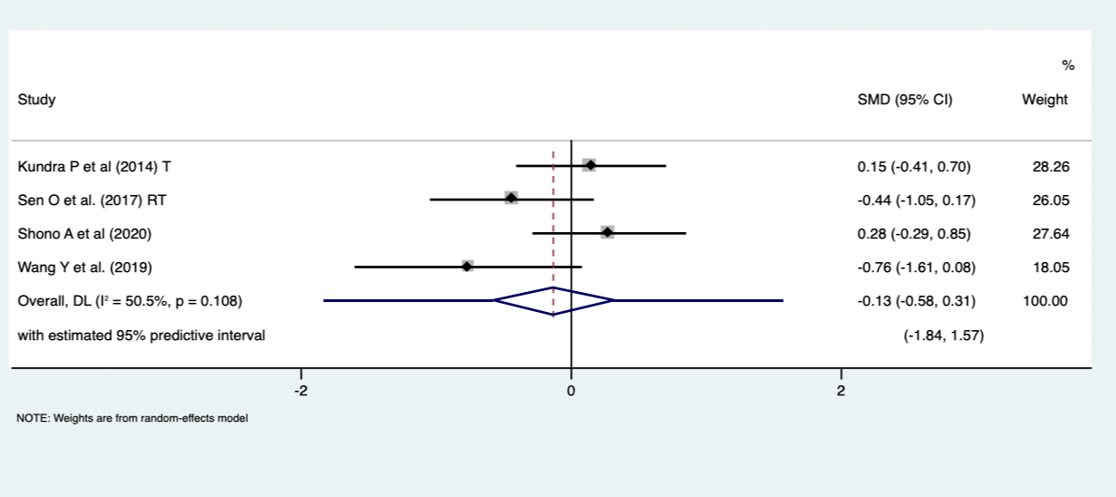


**Figure S23. Forest plot for estimation of the predictive interval of true effect on MAP for MPEEP vs HPEEP.**

Data are presented as standardized mean difference and 95% confidence intervals and 95% predictive intervals. The diamond symbolizes the summary estimate, which represents the combined effect size of all included studies. It provides an overall measure of the difference in MAP outcomes between moderate PEEP and high PEEP.

**Abbreviations**: I^2^: Heterogeneity; SMD: standardized mean difference; CI: confidence interval; MAP: mean arterial pressure; MPEEP – moderate positive end-expiratory pressure; HPEEP – high positive end-expiratory pressure.


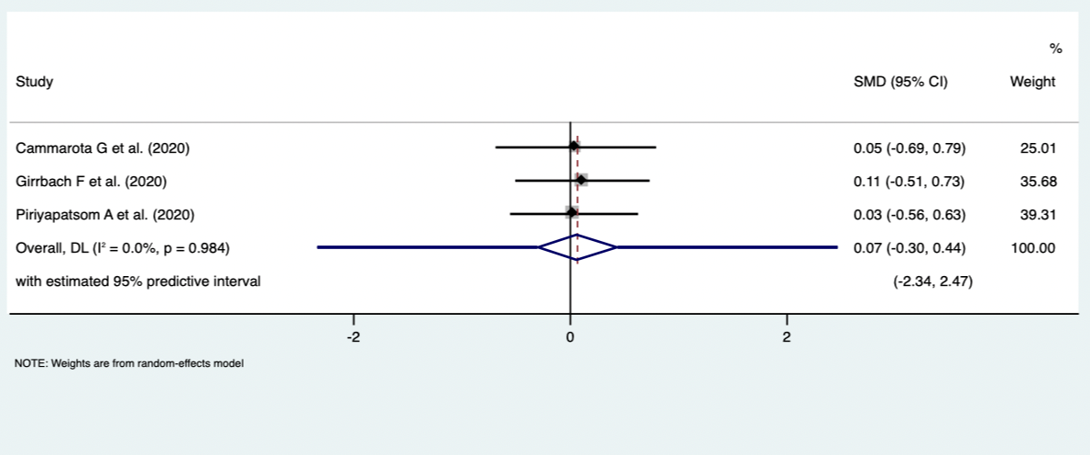


**Figure S24. Forest plot for estimation of the predictive interval of true effect on** **MAP for MPEEP vs iPEEP.**

Data are presented as standardized mean difference and 95% confidence intervals and 95% predictive intervals. The diamond symbolizes the summary estimate, which represents the combined effect size of all included studies. It provides an overall measure of the difference in MAP outcomes between moderate PEEP and individualized PEEP.

**Abbreviations**: I^2^: the ratio of excess dispersion to total dispersion; SMD: standardized mean difference; CI: confidence interval; MAP: mean arterial pressure; MPEEP – moderate positive end-expiratory pressure; iPEEP – individualized positive end-expiratory pressure.


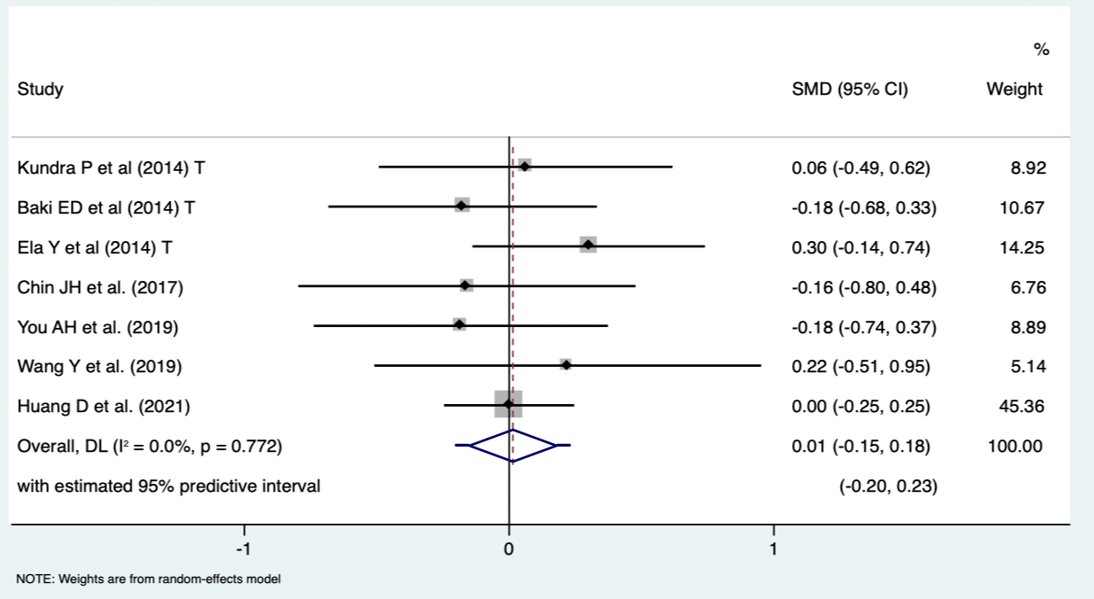


**Figure S25. Forest plot for estimation of the predictive interval of true effect on HR for LPEEP vs MPEEP.**

Data are presented as standardized mean difference and 95% confidence intervals and 95% predictive intervals. The diamond symbolizes the summary estimate, which represents the combined effect size of all included studies. It provides an overall measure of the difference in HR outcomes between low PEEP and moderate PEEP.

**Abbreviations**: I^2^: the ratio of excess dispersion to total dispersion; SMD: standardized mean difference; CI: confidence interval; HR: heart rate; LPEEP – low positive end-expiratory pressure; MPEEP – moderate positive end-expiratory pressure.


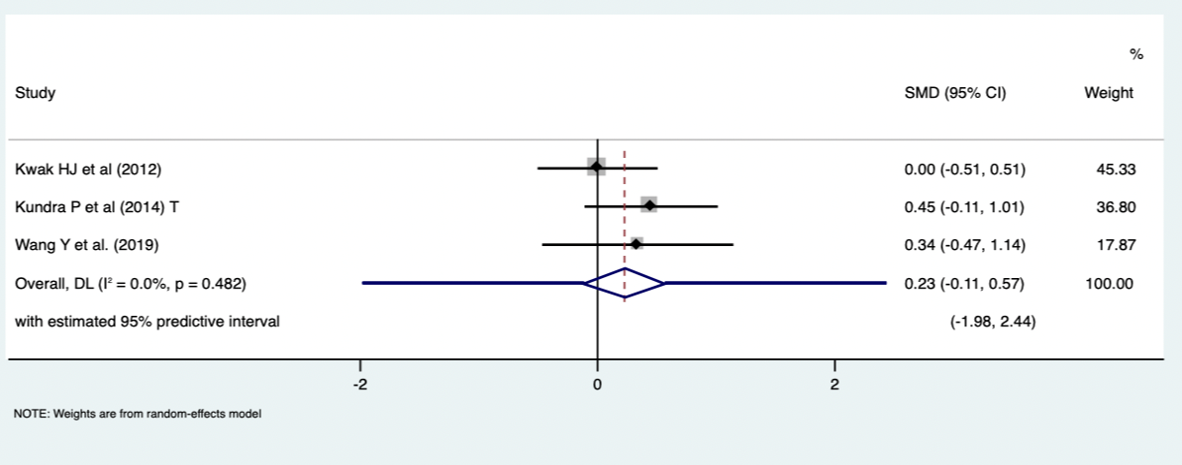


**Figure S26. Forest plot for estimation of the predictive interval of true effect on HR for LPEEP vs HPEEP.**

Data are presented as standardized mean difference and 95% confidence intervals and 95% predictive intervals. The diamond symbolizes the summary estimate, which represents the combined effect size of all included studies. It provides an overall measure of the difference in HR outcomes between low PEEP and high PEEP.

**Abbreviations**: I^2^: the ratio of excess dispersion to total dispersion; SMD: standardized mean difference; CI: confidence interval; HR: heart rate; LPEEP – low positive end-expiratory pressure; HPEEP – high positive end-expiratory pressure.


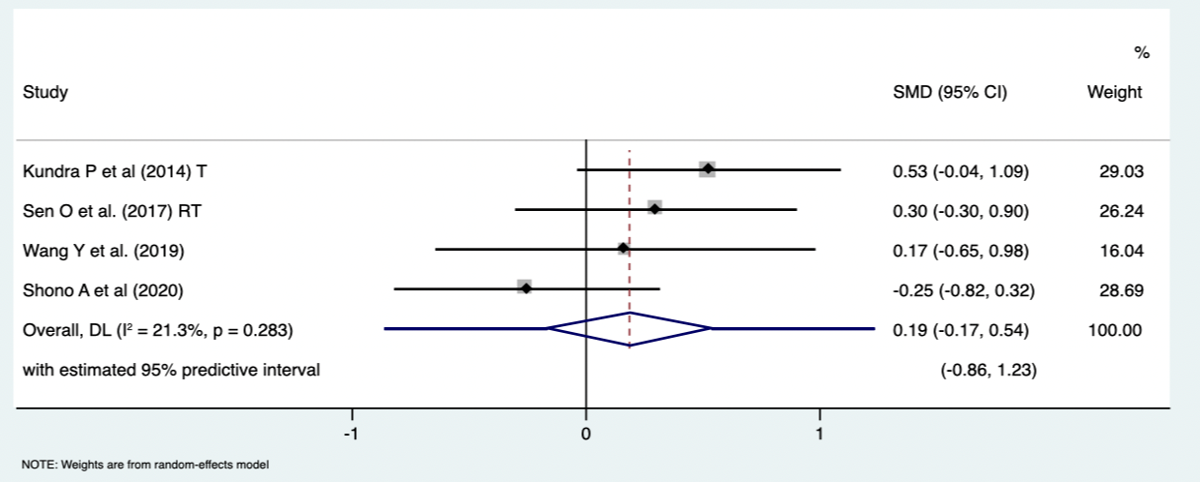


**Figure S27. Forest plot for estimation of the predictive interval of true effect on HR for MPEEP vs HPEEP.**

Data are presented as standardized mean difference and 95% confidence intervals and 95% predictive intervals. The diamond symbolizes the summary estimate, which represents the combined effect size of all included studies. It provides an overall measure of the difference in HR outcomes between moderate PEEP and high PEEP.

**Abbreviations**: I^2^: the ratio of excess dispersion to total dispersion; SMD: standardized mean difference; CI: confidence interval; HR: heart rate; MPEEP – moderate positive end-expiratory pressure; HPEEP – high positive end-expiratory pressure.


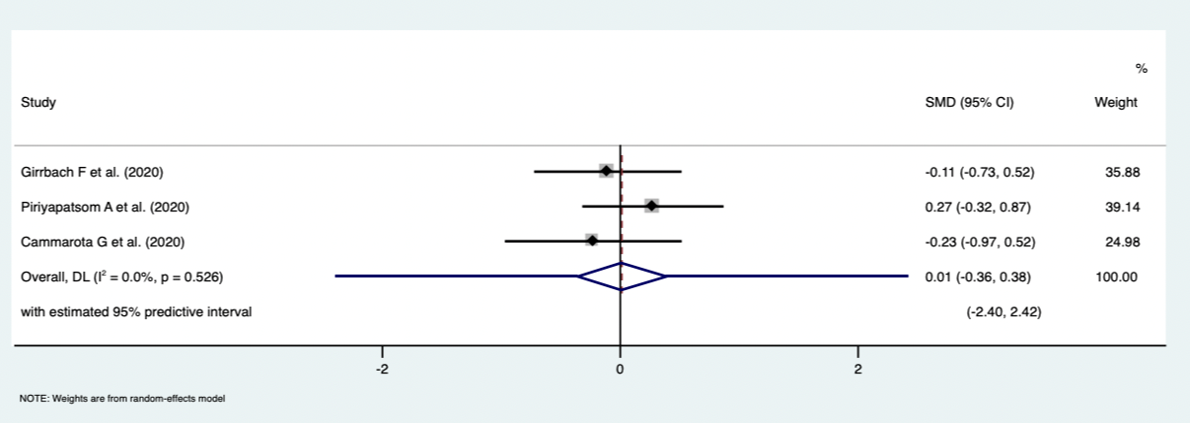


**Figure S28. Forest plot for estimation of the predictive interval of true effect on HR for MPEEP vs iPEEP.**

Data are presented as standardized mean difference and 95% confidence intervals and 95% predictive intervals. The diamond symbolizes the summary estimate, which represents the combined effect size of all included studies. It provides an overall measure of the difference in MAP outcomes between moderate PEEP and individualized PEEP.

**Abbreviations**: I^2^: the ratio of excess dispersion to total dispersion; SMD: standardized mean difference; CI: confidence interval; HR: heart rate; MPEEP – moderate positive end-expiratory pressure; iPEEP – individualised positive end-expiratory pressure.


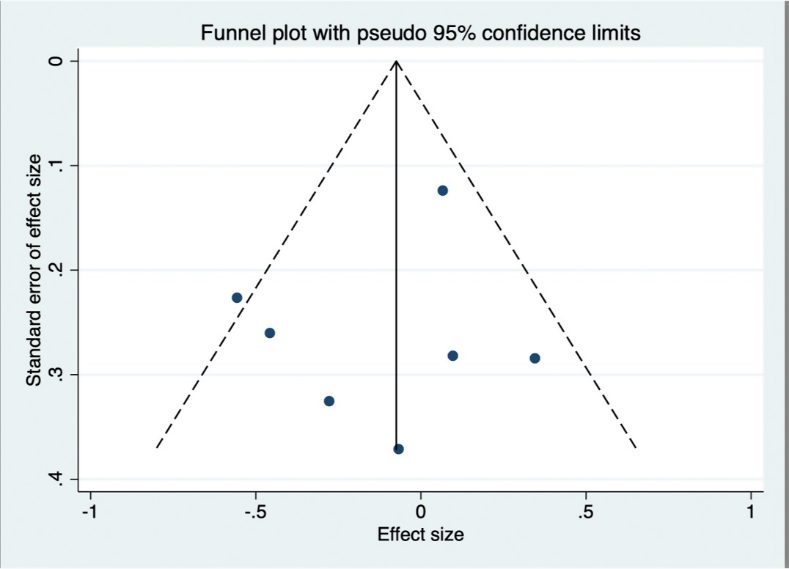


**Figure S29. Funnel plot of publication bias in MAP for LPEEP vs MPEEP**

А funnel plot of effect size and standard error of effect size in MAP for LPEEP vs MPEEP. The triangular lines on both sides of the summary estimate represent the precision measures, such as pseudo 95 % confidence intervals. The middle solid line indicates the overall effect of the meta-analysis. Each dot represents the individual study.

**Abbreviations**: MAP: mean arterial pressure; LPEEP – low positive end-expiratory pressure MPEEP – moderate positive end-expiratory pressure.


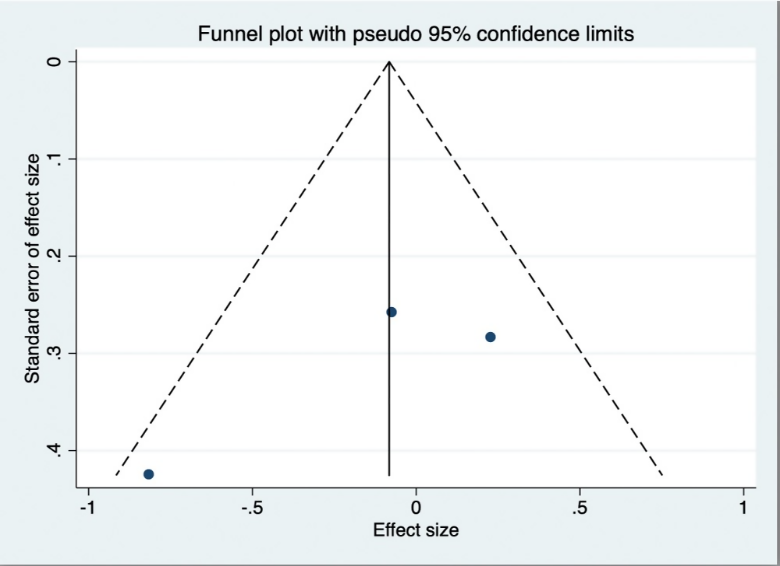


**Figure S30. Funnel plot of publication bias in MAP for LPEEP vs HPEEP**

А funnel plot of effect size and standard error of effect size in MAP for LPEEP vs HPEEP. The triangular lines on both sides of the summary estimate represent the precision measures, such as pseudo 95 % confidence intervals. The middle solid line indicates the overall effect of the meta-analysis. Each dot represents the individual study.

**Abbreviations**: MAP: mean arterial pressure; LPEEP – low positive end-expiratory pressure HPEEP – high positive end-expiratory pressure.


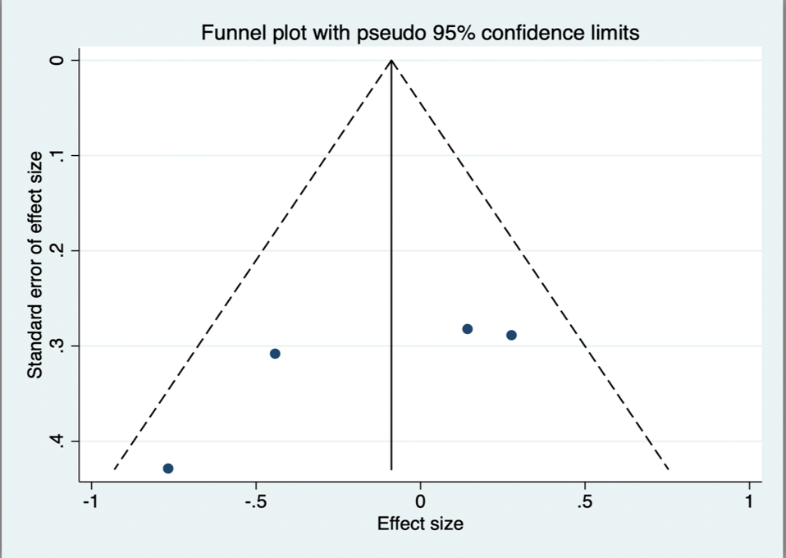


**Figure S31. Funnel plot of publication bias in MAP for MPEEP vs HPEEP**

А funnel plot of effect size and standard error of effect size in MAP for MPEEP vs HPEEP. The triangular lines on both sides of the summary estimate represent the precision measures, such as pseudo 95 % confidence intervals. The middle solid line indicates the overall effect of the meta-analysis. Each dot represents the individual study.

**Abbreviations**: MAP: mean arterial pressure; MPEEP – moderate positive end-expiratory pressure HPEEP – high positive end-expiratory pressure.


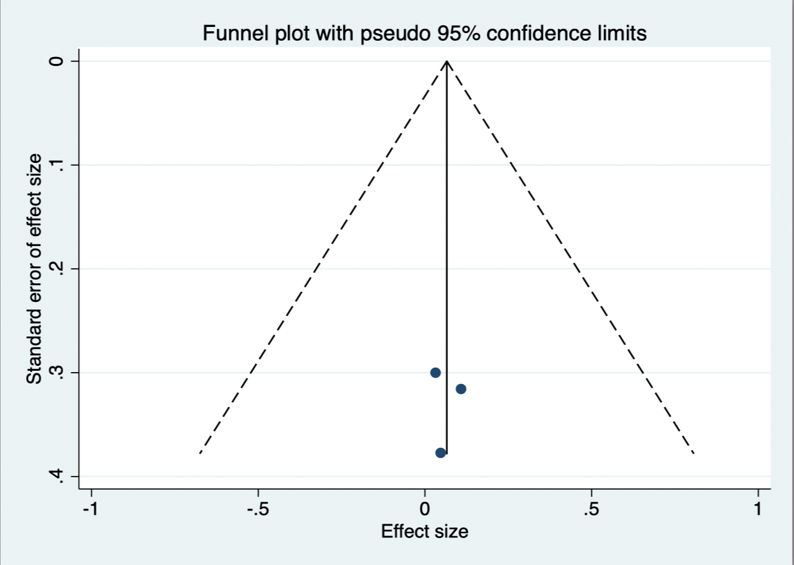


**Figure S32. Funnel plot of publication bias in MAP for MPEEP vs iPEEP**

А funnel plot of effect size and standard error of effect size in MAP for MPEEP vs iPEEP. The triangular lines on both sides of the summary estimate represent the precision measures, such as pseudo 95 % confidence intervals. The middle solid line indicates the overall effect of the meta-analysis. Each dot represents the individual study.

**Abbreviations**: MAP: mean arterial pressure; MPEEP – moderate positive end-expiratory pressure; iPEEP – individualised positive end-expiratory pressure.


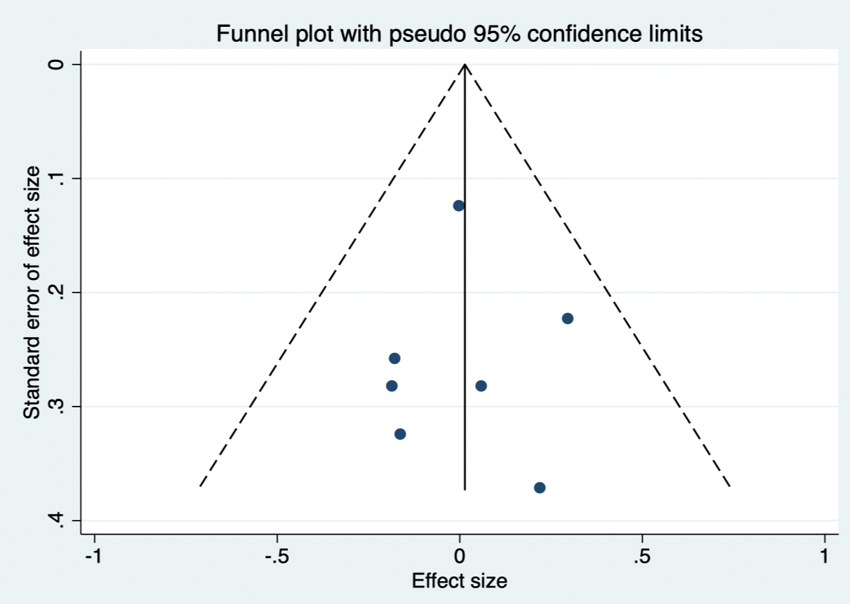


**Figure S33. Funnel plot of publication bias in HR for LPEEP vs MPEEP**

А funnel plot of effect size and standard error of effect size in HR for LPEEP vs MPEEP. The triangular lines on both sides of the summary estimate represent the precision measures, such as pseudo 95 % confidence intervals. The middle solid line indicates the overall effect of the meta-analysis. Each dot represents the individual study.

**Abbreviations**: HR: heart rate; LPEEP – low positive end-expiratory pressure MPEEP – moderate positive end-expiratory pressure.


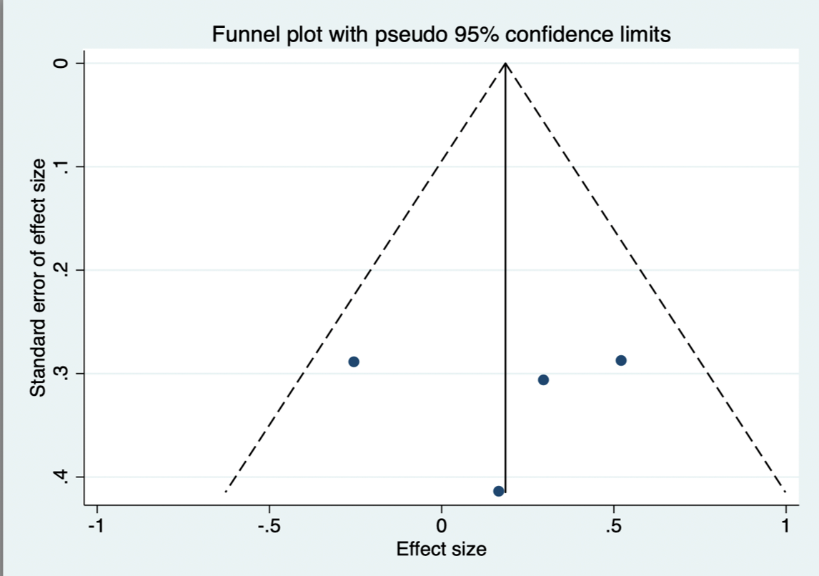


**Figure S34. Funnel plot of publication bias in HR for MPEEP vs HPEEP**

А funnel plot of effect size and standard error of effect size in HR for MPEEP vs HPEEP. The triangular lines on both sides of the summary estimate represent the precision measures, such as pseudo 95 % confidence intervals. The middle solid line indicates the overall effect of the meta-analysis. Each dot represents the individual study.

**Abbreviations**: HR: heart rate; MPEEP – moderate positive end-expiratory pressure HPEEP – high positive end-expiratory pressure.


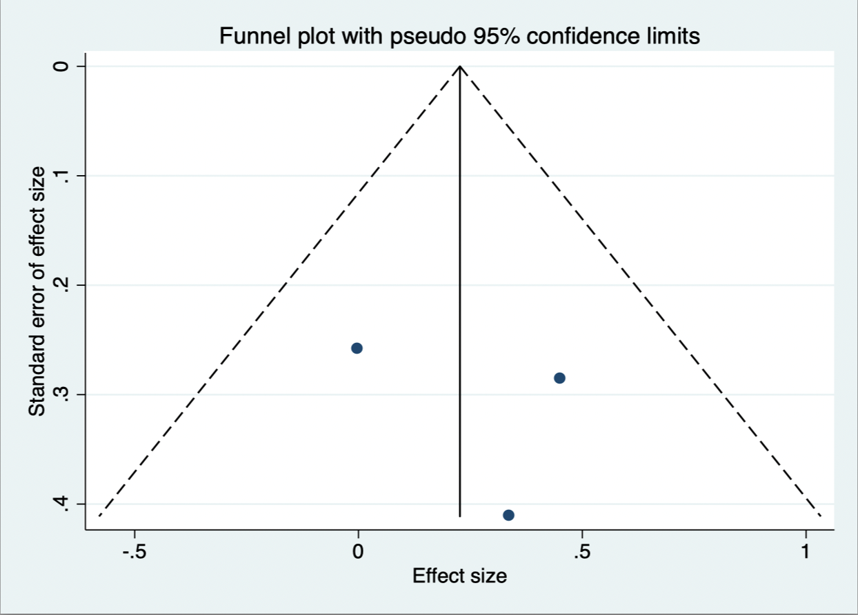


Figure 34 supplementary

**Figure S35. Funnel plot of publication bias in HR for LPEEP vs HPEEP**

А funnel plot of effect size and standard error of effect size in HR for LPEEP vs HPEEP. The triangular lines on both sides of the summary estimate represent the precision measures, such as pseudo 95 % confidence intervals. The middle solid line indicates the overall effect of the meta-analysis. Each dot represents the individual study.

**Abbreviations**: HR: heart rate; LPEEP – low positive end-expiratory pressure HPEEP – high positive end-expiratory pressure.


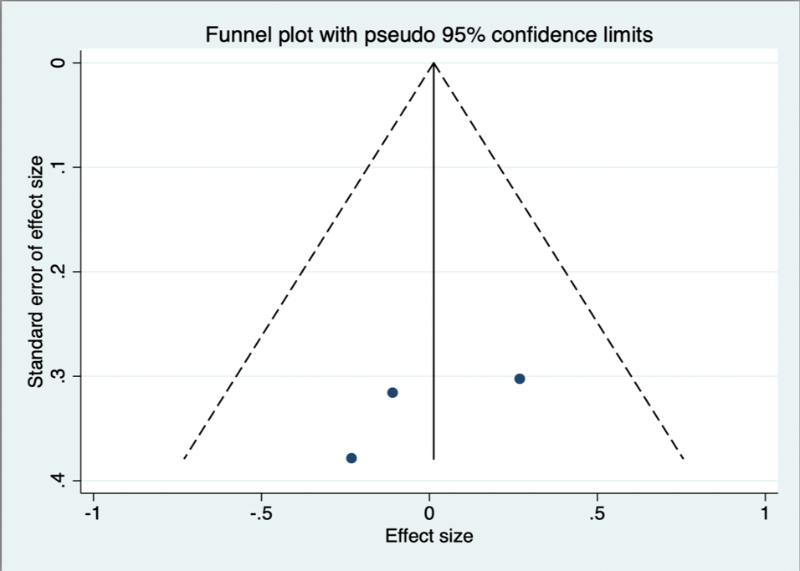


**Figure S36. Funnel plot of publication bias in HR for MPEEP vs iPEEP**

А funnel plot of effect size and standard error of effect size in HR for MPEEP vs iPEEP. The triangular lines on both sides of the summary estimate represent the precision measures, such as pseudo 95 % confidence intervals. The middle solid line indicates the overall effect of the meta-analysis. Each dot represents the individual study.

**Abbreviations**: HR: heart rate; MPEEP – moderate positive end-expiratory pressure; iPEEP – individualised positive end-expiratory pressure.

**Influence of the tidal volume on study outcomes (meta-regression)**

**Arterial partial pressure of oxygen (PaO_2_)**

Meta-regression for the influence of tidal volume value on PaO_2_ did not find significant relationship between tidal volume and PaO_2_ in LPEEP vs MPEEP RCTs (the regression coefficient -0.59, R^2^ 3.44%, p=0.239) but found significant variation of the true effect (Qres = 30.97, p<0.0001)(**Fig. S37**).


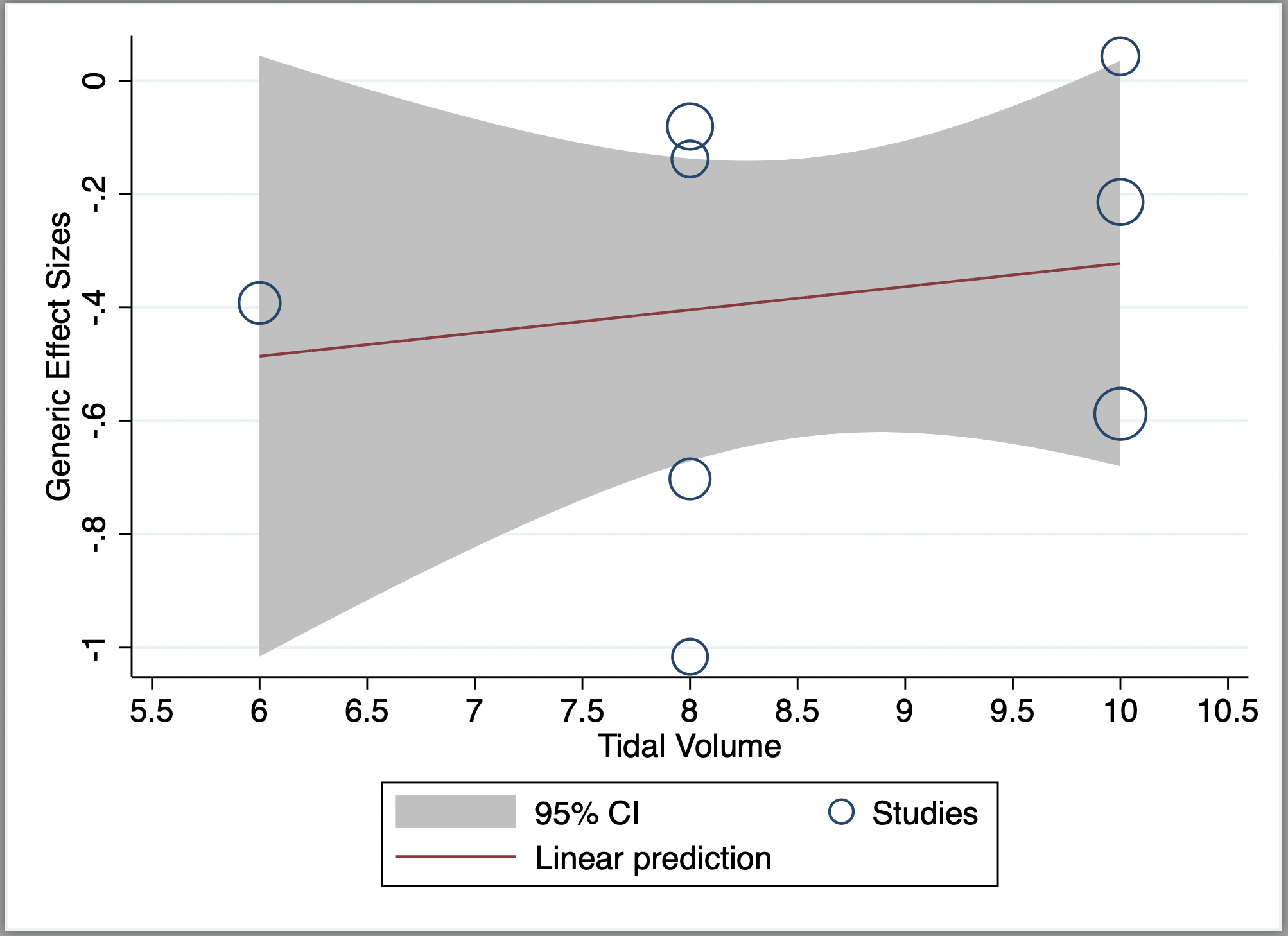


**Figure S37. Relationship between PaO_2_ and tidal volume in RCTs comparing LPEEP vs MPEEP.**

Data are presented as effect sizes (Cohen’s d) and 95% confidence intervals. Each bubble represents one randomized controlled trial. The circle size represents the sample size of the trial.

**Abbreviations**: CI: confidence interval; PaO2: arterial partial pressure of oxygen; LPEEP – low positive end-expiratory pressure; MPEEP – moderate positive end-expiratory pressure.

Meta-regression for the influence of tidal volume value on PaO_2_ did not find significant relationship between tidal volume and PaO_2_ in LPEEP vs HPEEP RCTs (the regression coefficient -1.13, R^2^ 40.00%, p=0.102) but found significant variation of the true effect (Qres = 10.30, p=0.006)(**Fig. S38**).


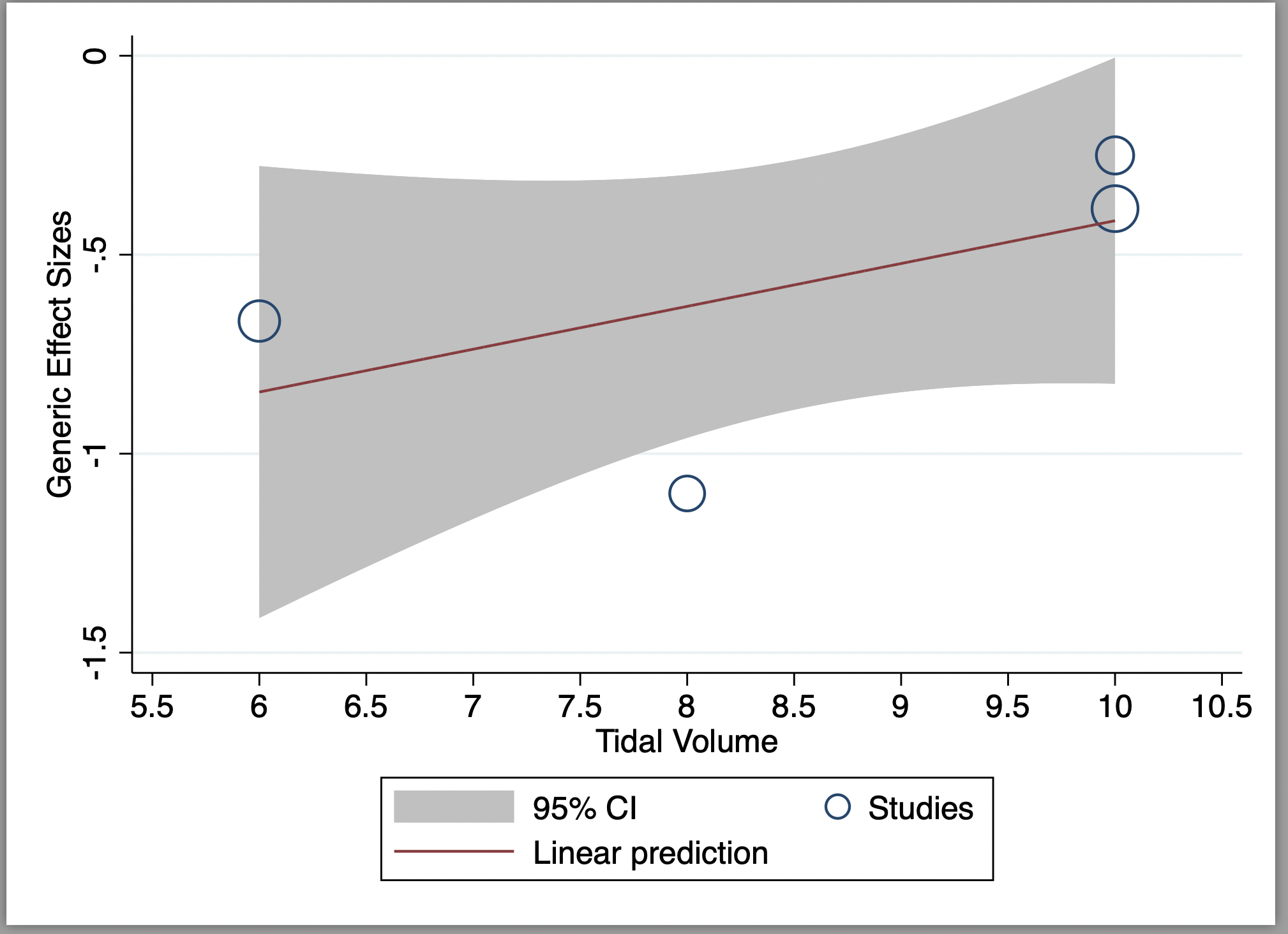


**Figure S38. Relationship between PaO_2_ and tidal volume in RCTs comparing LPEEP vs HPEEP.**

Data are presented as effect sizes (Cohen’s d) and 95% confidence intervals. Each bubble represents one randomized controlled trial. The circle size represents the sample size of the trial.

**Abbreviations**: CI: confidence interval; PaO2: arterial partial pressure of oxygen; LPEEP – low positive end-expiratory pressure; HPEEP – high positive end-expiratory pressure

Meta-regression for the influence of tidal volume value on PaO_2_ did not find significant relationship between tidal volume and PaO_2_ in MPEEP vs HPEEP RCTs (the regression coefficient 0.24, R^2^ 0.00%, p=0.672) but found significant variation of the true effect (Qres = 4.75, p=0.093)(**Fig. S39**).

**
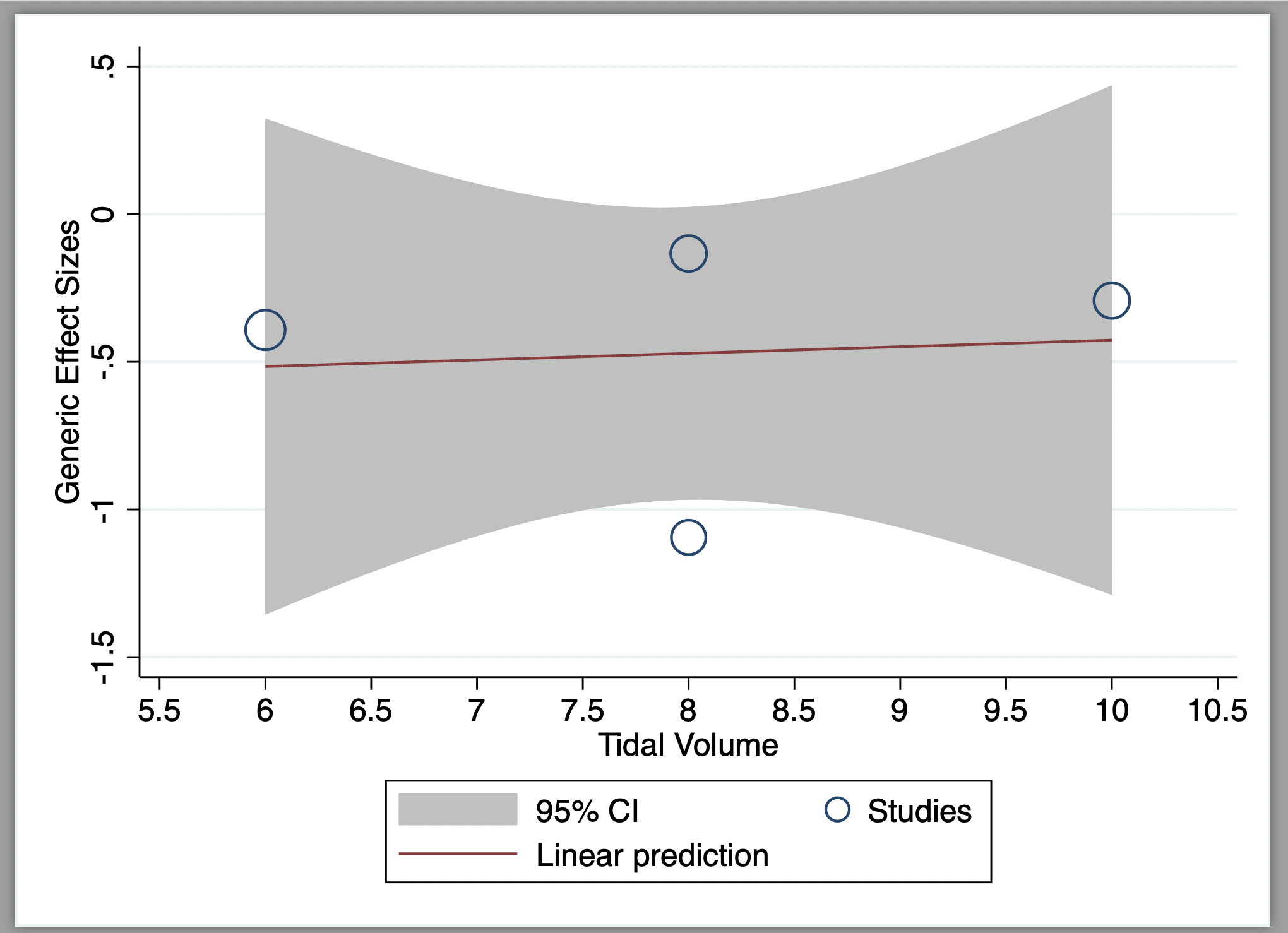
Figure S39. Relationship between PaO_2_ and tidal volume in RCTs comparing MPEEP vs HPEEP.**

Data are presented as effect sizes (Cohen’s d) and 95% confidence intervals. Each bubble represents one randomized controlled trial. The circle size represents the sample size of the trial.

**Abbreviations**: CI: confidence interval; PaO2: arterial partial pressure of oxygen; MPEEP – moderate positive end-expiratory pressure; HPEEP – high positive end-expiratory pressure

**Arterial partial pressure of oxygen to inspiratory oxygen fraction (PaO_2_/FiO_2_)**

Meta-regression for the influence of tidal volume value on PaO_2_/FiO_2_ did not find significant relationship between tidal volume and PaO_2_/FiO_2_ in LPEEP vs MPEEP RCTs (the regression coefficient 2.74, R^2^ 0.00%, p=0.522) but found significant variation of the true effect (Qres = 256.51, p<0.0001)(**Fig. S40**).

**
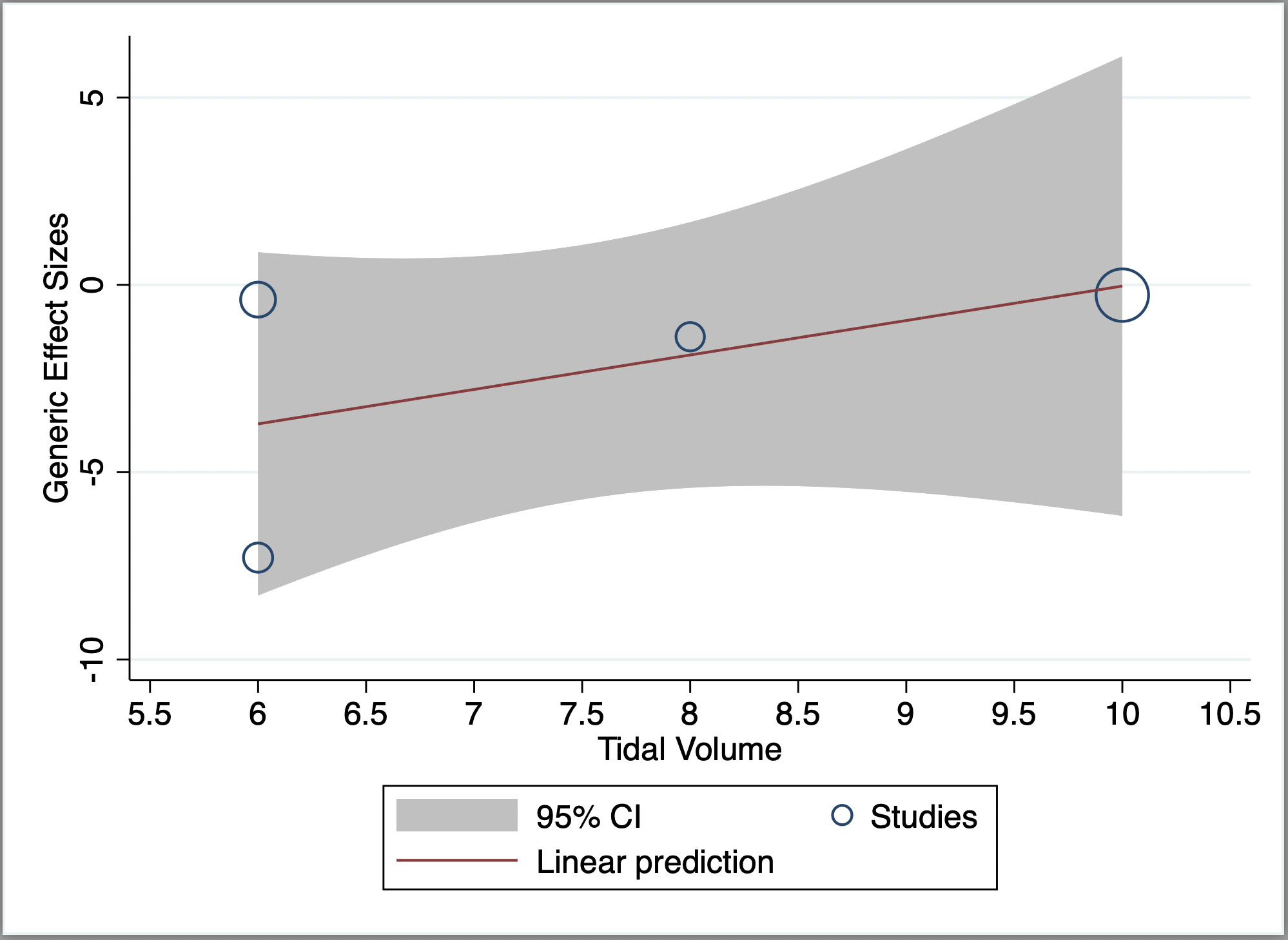
**

**Figure S40. Relationship between PaO_2_/FiO_2_ and tidal volume in RCTs comparing LPEEP vs MPEEP.**

Data are presented as effect sizes (Cohen’s d) and 95% confidence intervals. Each bubble represents one randomized controlled trial. The circle size represents the sample size of the trial.

**Abbreviations**: CI: confidence interval; PaO_2_: arterial partial pressure of oxygen; FiO_2_: inspiratory fraction of oxygen; LPEEP – low positive end-expiratory pressure; MPEEP – moderate positive end-expiratory pressure.

Meta-regression for the influence of tidal volume value on PaO_2_/FiO_2_ did not find significant relationship between tidal volume and PaO_2_/FiO_2_ in LPEEP vs HPEEP RCTs (the regression coefficient -0.06, R^2^ 0.00%, p=0.887), null hypothesis of no residual heterogeneity was not rejected (Qres = 1.54, p=0.215)(**Fig. S41**).

**
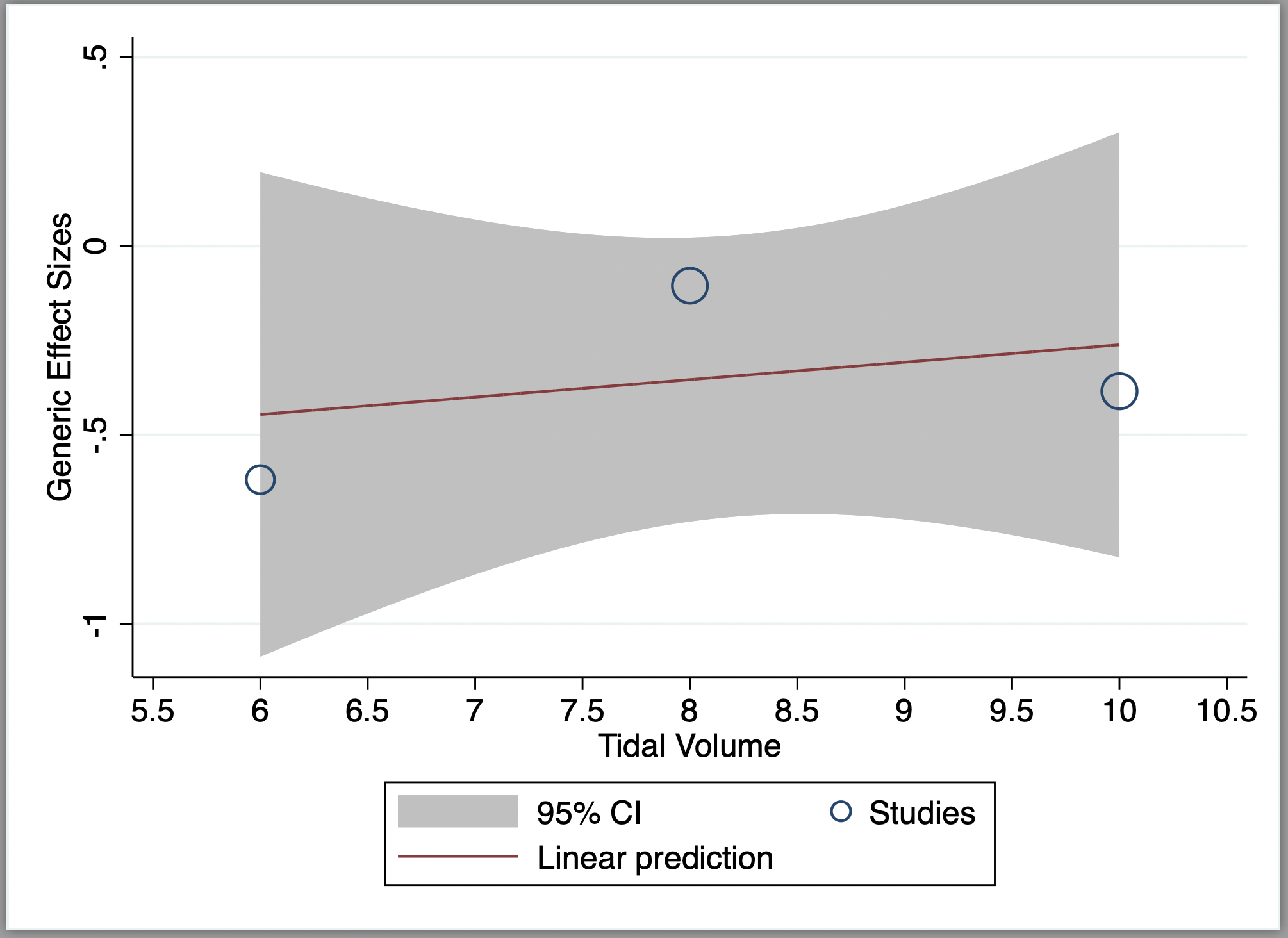
Figure S41. Relationship between PaO_2_/FiO_2_ and tidal volume in RCTs comparing LPEEP vs HPEEP.**

Data are presented as effect sizes (Cohen’s d) and 95% confidence intervals. Each bubble represents one randomized controlled trial. The circle size represents the sample size of the trial.

**Abbreviations**: CI: confidence interval; PaO_2_: arterial partial pressure of oxygen; FiO_2_: inspiratory fraction of oxygen; LPEEP – low positive end-expiratory pressure; HPEEP – high positive end-expiratory pressure.

**Dynamic compliance (Cdyn)**

Meta-regression for the influence of tidal volume value on Cdyn did not find significant relationship between tidal volume and Cdyn in LPEEP vs MPEEP RCTs (the regression coefficient -0.36, R^2^ 0.00%, p=0.696) but found significant variation of the true effect (Qres = 65.44, p<0.0001)(**Figure S42**).


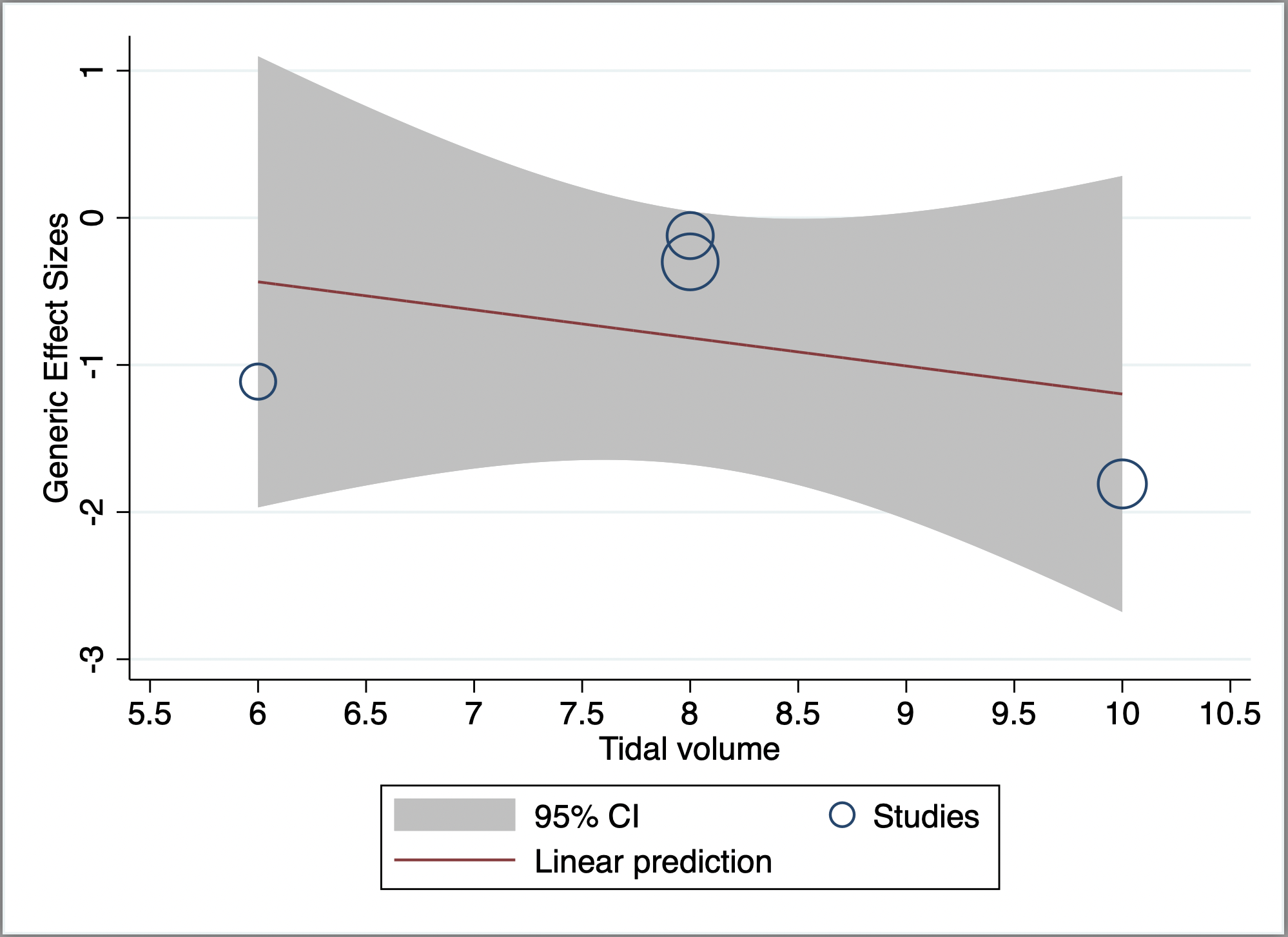


**Figure S42. Relationship between Cdyn and tidal volume in RCTs comparing LPEEP vs MPEEP.**

Data are presented as effect sizes (Cohen’s d) and 95% confidence intervals. Each bubble represents one randomized controlled trial. The circle size represents the sample size of the trial.

**Abbreviations**: CI: confidence interval; Cdyn: dynamic compliance of respiratory system; LPEEP – low positive end-expiratory pressure; MPEEP – moderate positive end-expiratory pressure.

Meta-regression for the influence of tidal volume value on Cdyn found significant decrease in Cdyn in patients with tidal volume more than 8 ml/kg as compared to less than 8 ml/kg in LPEEP vs HPEEP RCTs (the regression coefficient -1.37, R^2^ 82.82%, p=0.006), null hypothesis of no residual heterogeneity was not rejected (Qres = 3.84, p=0.147 (**Fig. S43**).


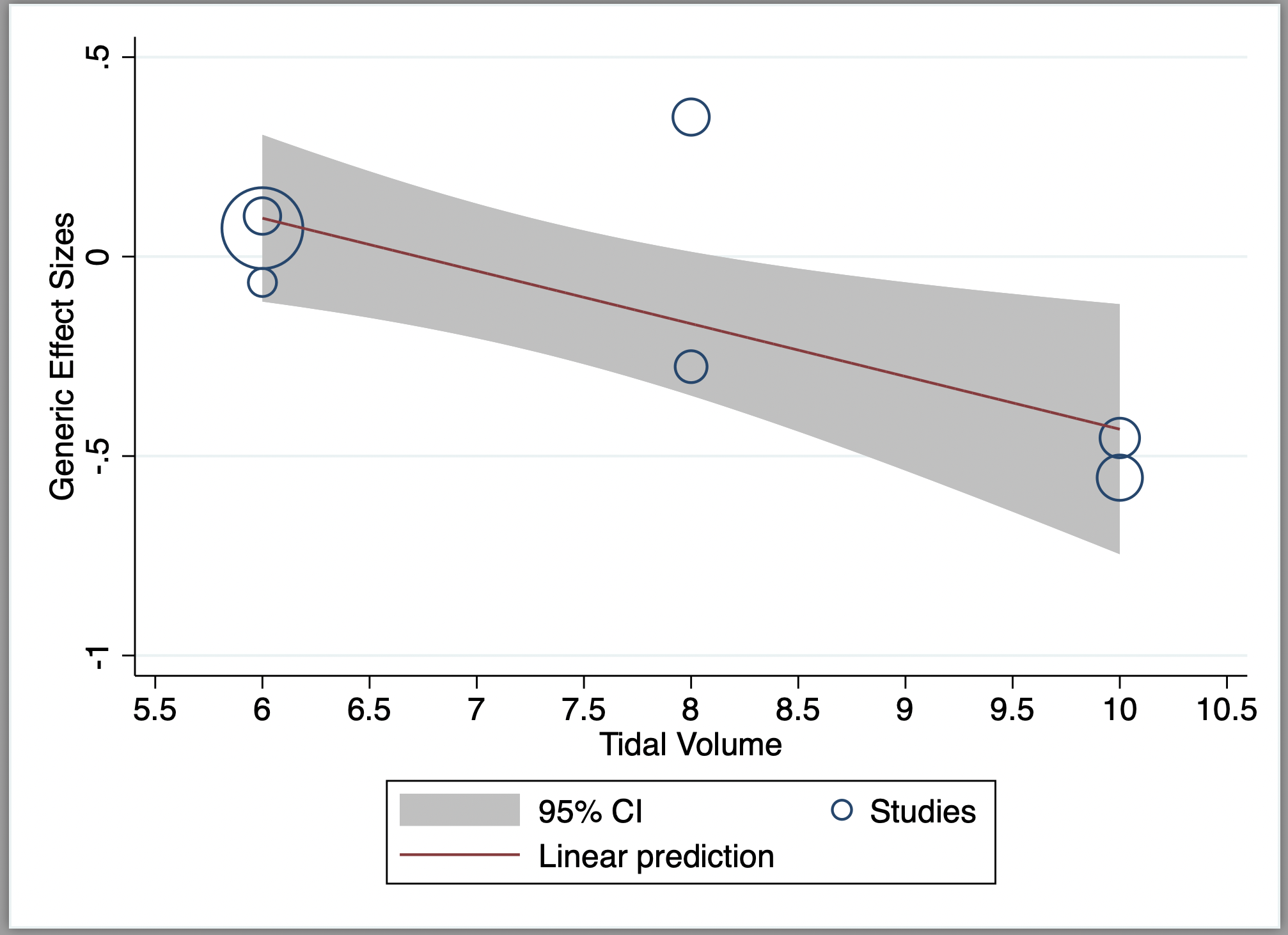


**Figure S43. Relationship between Cdyn and tidal volume in RCTs comparing LPEEP vs HPEEP.**

Data are presented as effect sizes (Cohen’s d) and 95% confidence intervals. Each bubble represents one randomized controlled trial. The circle size represents the sample size of the trial.

**Abbreviations**: CI: confidence interval; Cdyn: dynamic compliance of respiratory system; LPEEP – low positive end-expiratory pressure; HPEEP – high positive end-expiratory pressure.

**Mean arterial pressure (MAP) and the heart rate (HR)**

Meta-regression for the influence of tidal volume value on MAP found significant decrease in MAP in patients with tidal volume more than 8 ml/kg as compared to less than 8 ml/kg in LPEEP vs MPEEP RCTs (the regression coefficient -0.58, R^2^ 100.00%, p=0.003), null hypothesis of no residual heterogeneity was not rejected (Qres = 2.31, p=0.804 (**Figure S44**).


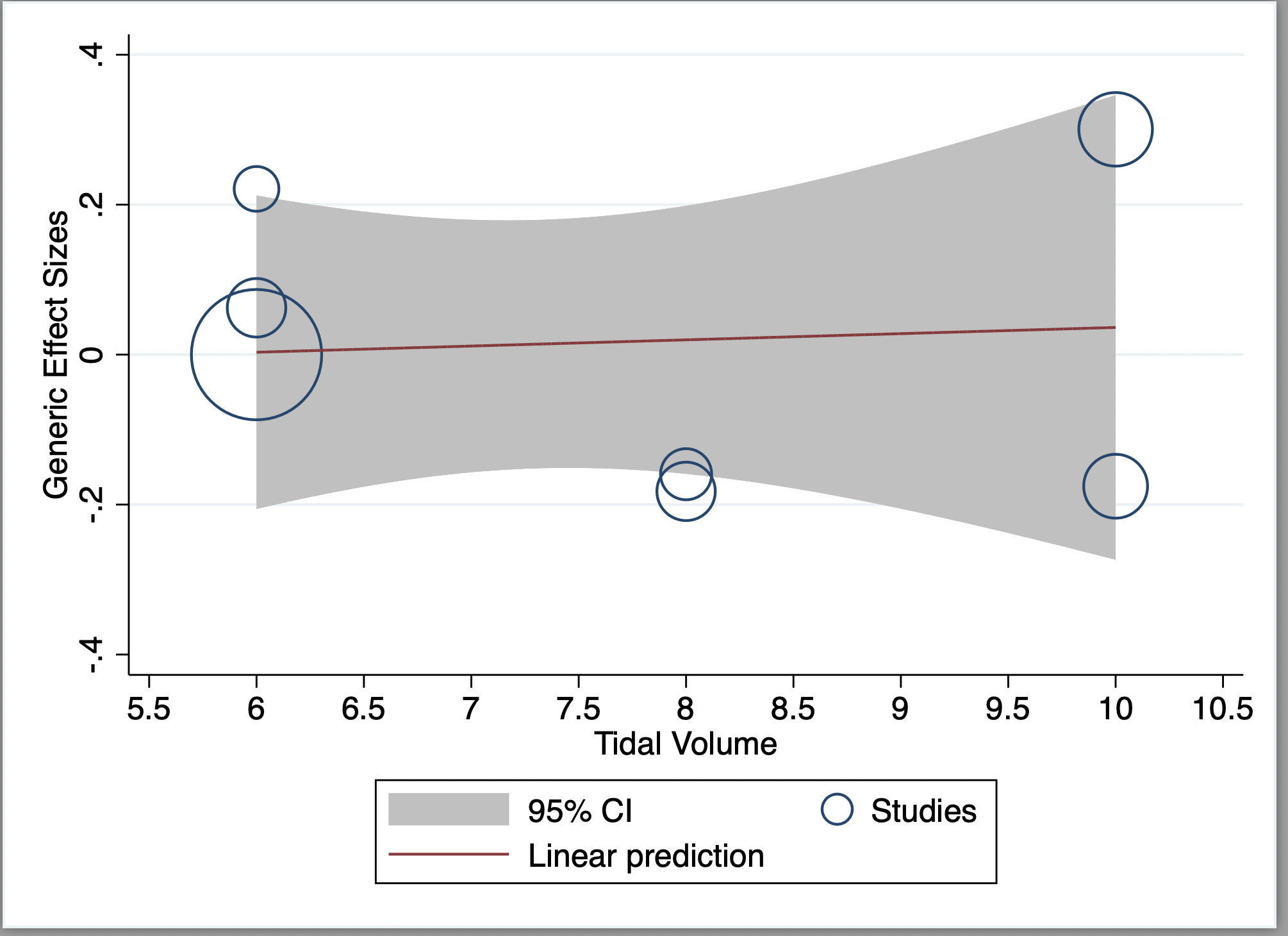


**Figure S44. Relationship between MAP and tidal volume in RCTs comparing LPEEP vs MPEEP.**

Data are presented as effect sizes (Cohen’s d) and 95% confidence intervals. Each bubble represents one randomized controlled trial. The circle size represents the sample size of the trial.

**Abbreviations**: CI: confidence interval; MAP: mean arterial pressure; LPEEP – low positive end-expiratory pressure; MPEEP – moderate positive end-expiratory pressure.

Meta-regression for the influence of tidal volume value on heart rate did not find significant relationship between tidal volume and HR in LPEEP vs MPEEP RCTs (the regression coefficient 0.11, R^2^ 64.70%, p=0.56), null hypothesis of no residual heterogeneity was not rejected (Qres = 2.97, p=0.704 (**Figure S45**).


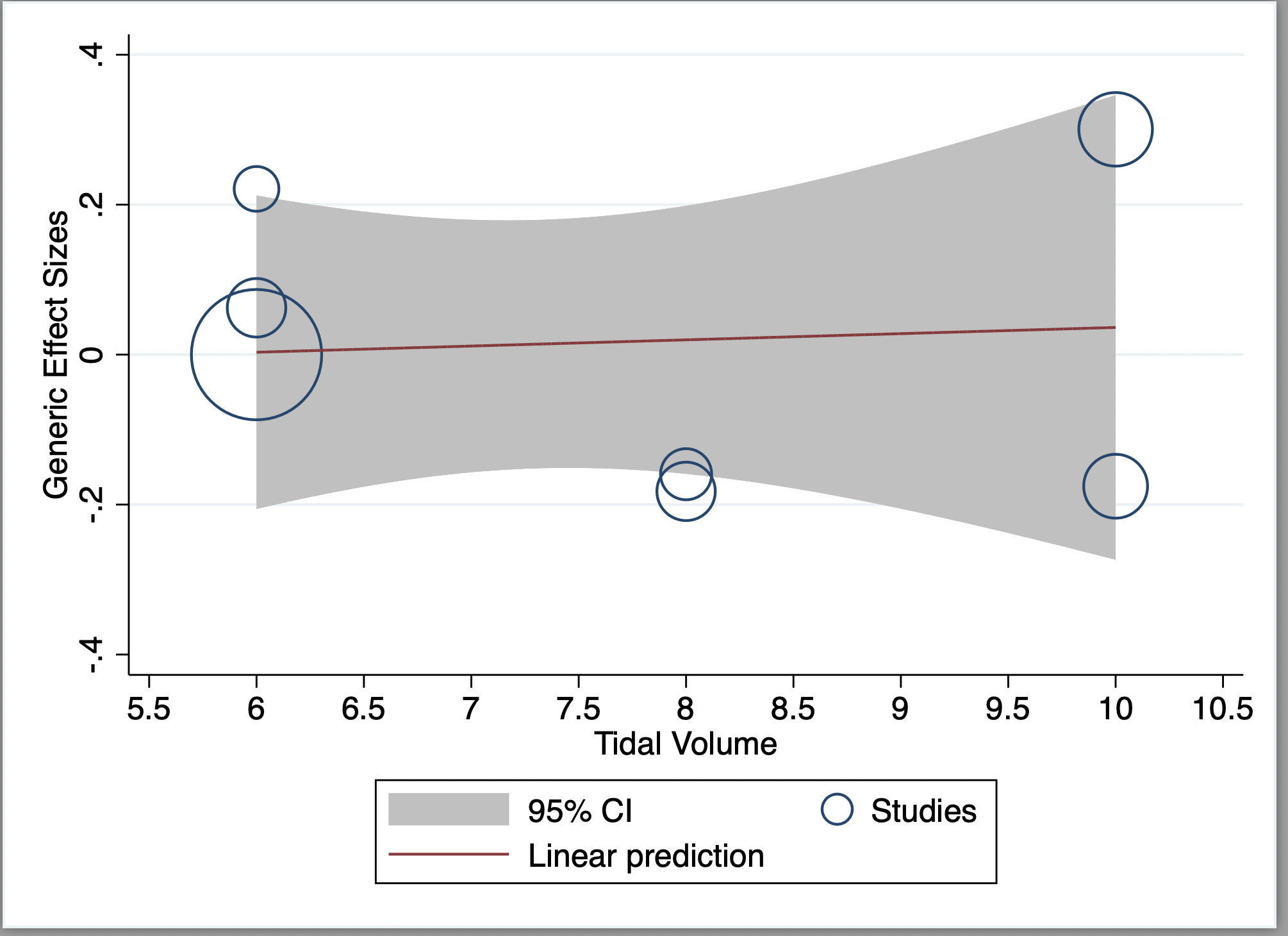


**Figure S45. Relationship between heart rate and tidal volume in RCTs comparing LPEEP vs MPEEP.**

Data are presented as effect sizes (Cohen’s d) and 95% confidence intervals. Each bubble represents one randomized controlled trial. The circle size represents the sample size of the trial.

**Abbreviations**: CI: confidence interval; LPEEP – low positive end-expiratory pressure; MPEEP – moderate positive end-expiratory pressure.

**Influence of patient position during surgery on study outcomes (meta-regression)**

**Arterial partial pressure of oxygen (PaO_2_)**

Meta-regression for the influence of patient position during surgery on PaO_2_ did not find significant relationship between patient position and PaO_2_ in LPEEP vs MPEEP RCTs (the regression coefficient 0.19, R^2^ 0.00%, p=0.581) null hypothesis of no residual heterogeneity was not rejected (Qres = 9.23, p=0.161(**Figure S46**).


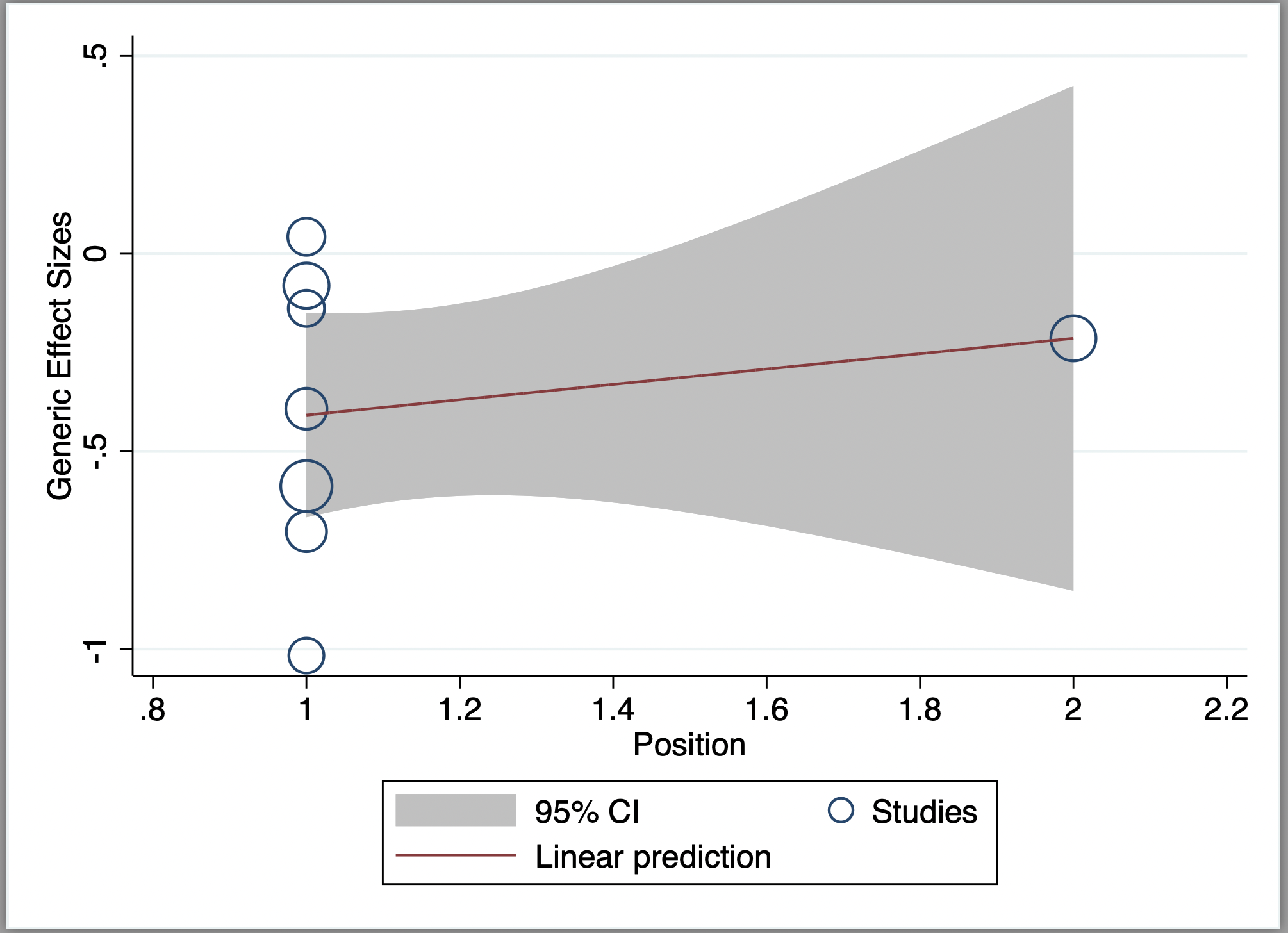


**Figure S46. Relationship between PaO_2_ and body position in RCTs comparing LPEEP vs MPEEP.**

Data are presented as effect sizes (Cohen’s d) and 95% confidence intervals. Each bubble represents one randomized controlled trial. The circle size represents the sample size of the trial. 1- Trendelenburg, 2-Reverse Trendelenburg

**Abbreviations**: CI: confidence interval; PaO2: arterial partial pressure of oxygen; LPEEP – low positive end-expiratory pressure; MPEEP – moderate positive end-expiratory pressure.

Meta-regression for the influence of patient position during surgery on PaO_2_ did not find significant relationship between patient position and PaO_2_ in LPEEP vs HPEEP RCTs (the regression coefficient -0.11, R^2^ 0.00%, p=0.811) null hypothesis of no residual heterogeneity was not rejected (Qres = 3.90, p=0.142(**Figure S47**).

**
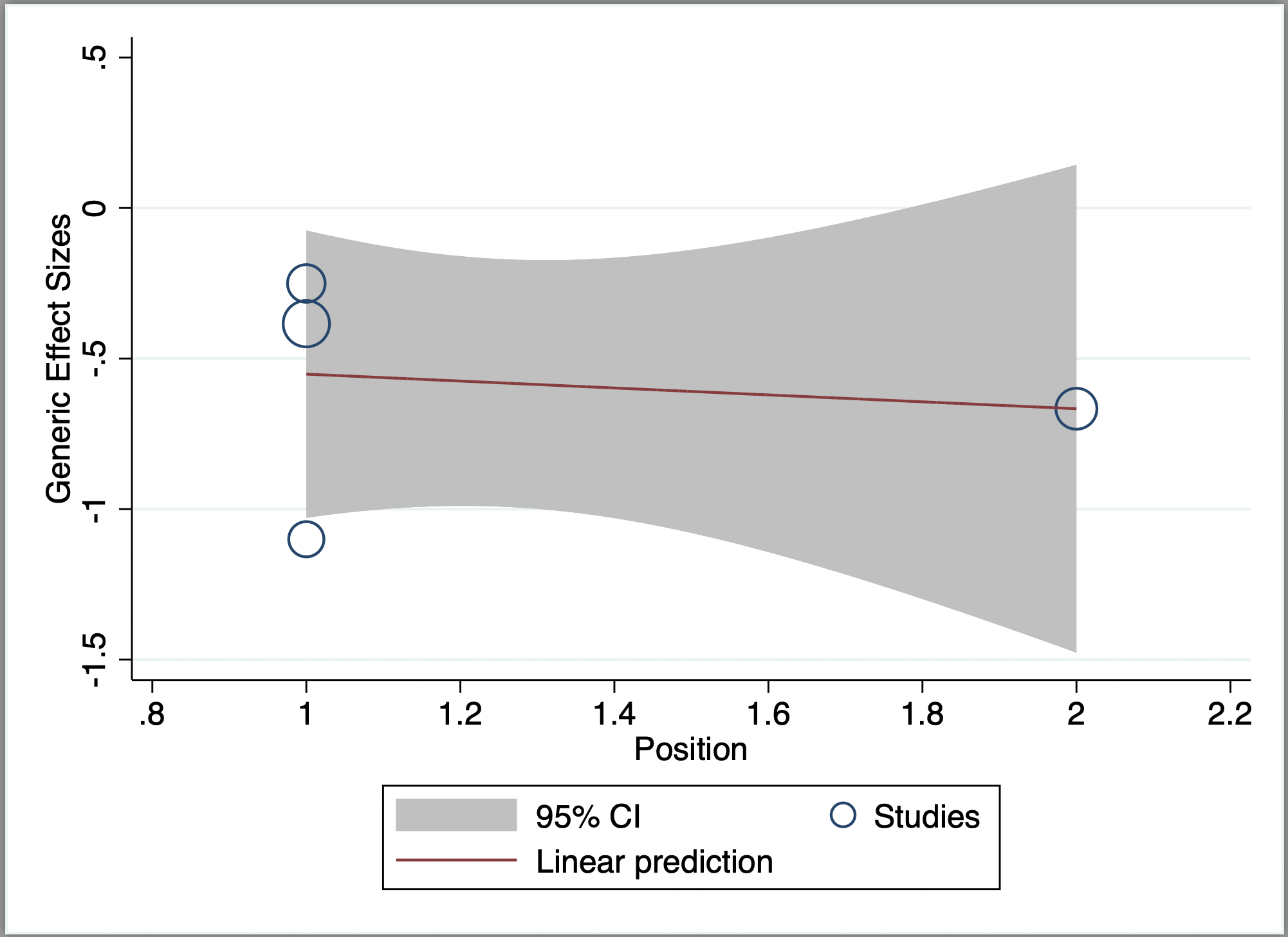
**

**Figure S47. Relationship between PaO_2_ and body position in RCTs comparing LPEEP vs HPEEP.**

Data are presented as effect sizes (Cohen’s d) and 95% confidence intervals. Each bubble represents one randomized controlled trial. The circle size represents the sample size of the trial. 1- Trendelenburg, 2-Reverse Trendelenburg

**Abbreviations**: CI: confidence interval; PaO2: arterial partial pressure of oxygen; LPEEP – low positive end-expiratory pressure; HPEEP – high positive end-expiratory pressure.

Meta-regression for the influence of patient position during surgery on PaO_2_ did not find significant relationship between patient position and PaO_2_ in MPEEP vs HPEEP RCTs (the regression coefficient -0.50, R^2^ 43.87%, p=0.177), null hypothesis of no residual heterogeneity was not rejected (Qres = 2.73, p=0.256(**Figure S48**).

**
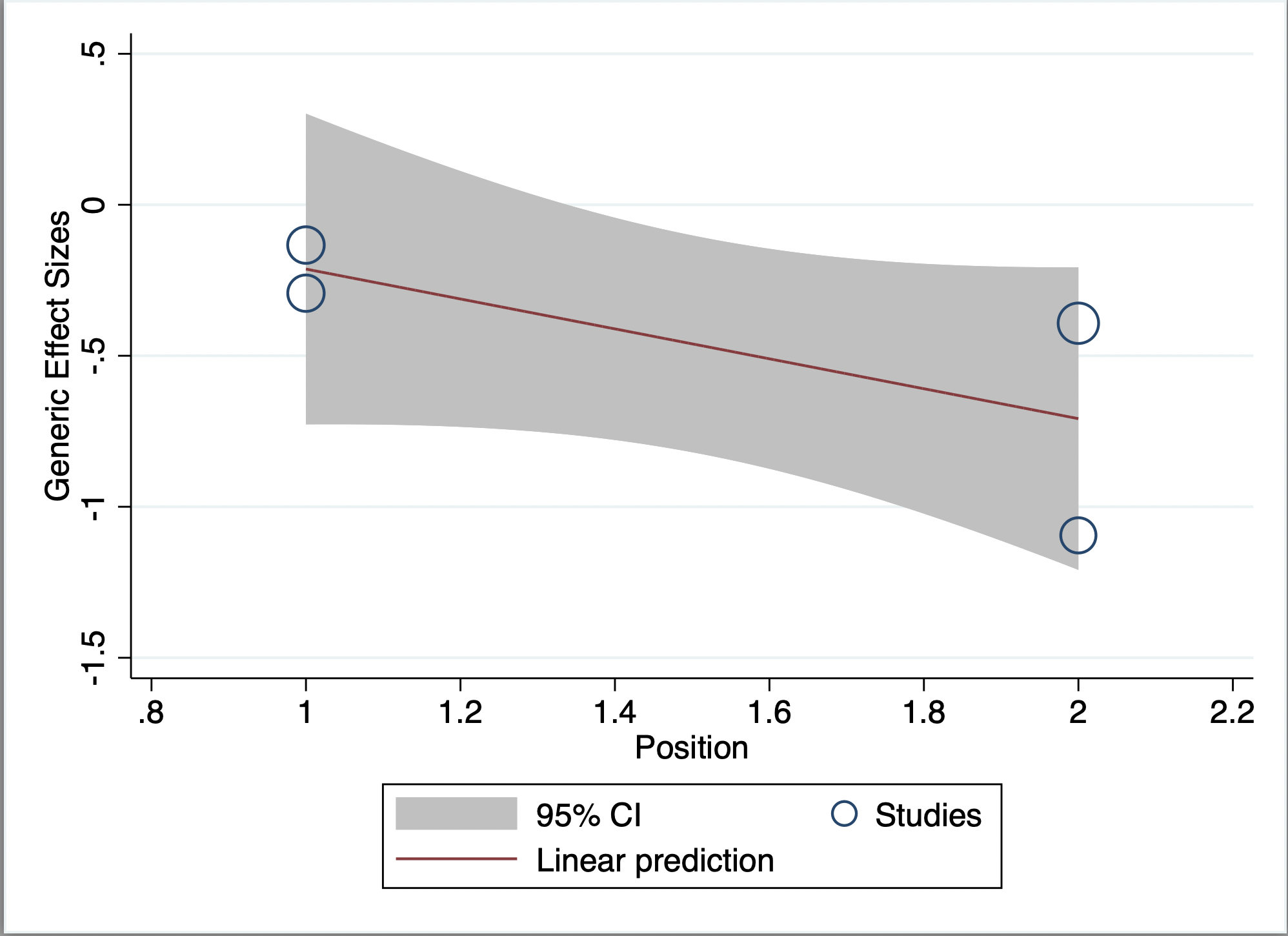
Figure S48. Relationship between PaO_2_ and body position in RCTs comparing MPEEP vs HPEEP.**

Data are presented as effect sizes (Cohen’s d) and 95% confidence intervals. Each bubble represents one randomized controlled trial. The circle size represents the sample size of the trial. 1- Trendelenburg, 2-Reverse Trendelenburg

**Abbreviations**: CI: confidence interval; PaO2: arterial partial pressure of oxygen; MPEEP – moderate positive end-expiratory pressure; HPEEP – high positive end-expiratory pressure.

**Arterial partial pressure of oxygen to inspiratory oxygen fraction (PaO_2_/FiO_2_)**

Meta-regression for the influence of patient position during surgery on PaO_2_/FiO_2_ did not find significant relationship between patient position and PaO_2_/FiO_2_  in LPEEP vs MPEEP RCTs (the regression coefficient 3.99, R^2^ 22.41%, p=0.173), but found significant variation of the true effect (Qres = 142.94, p=<0.0001)(**Figure S49**).

**
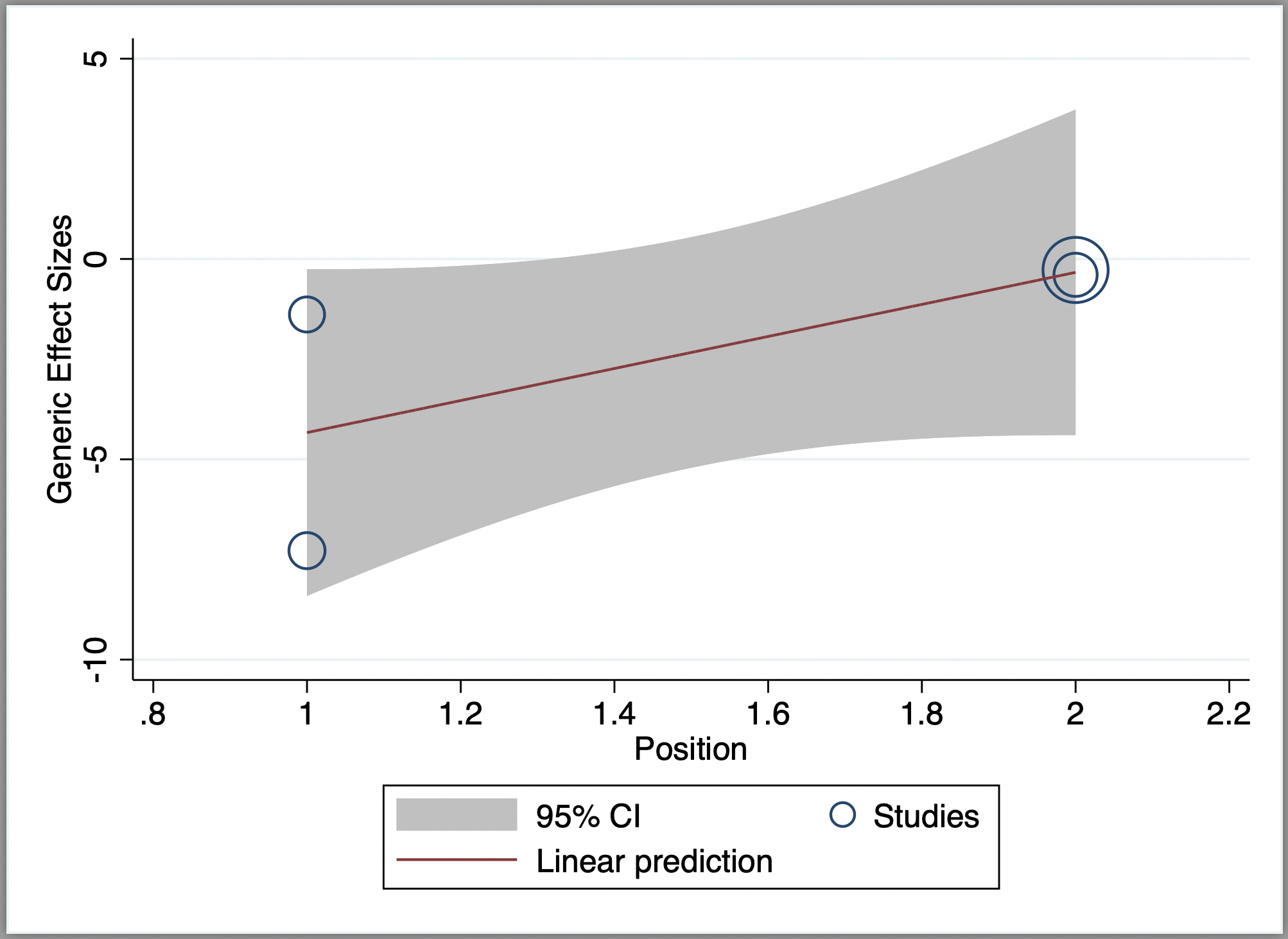
Figure S49. Relationship between PaO_2_/FiO_2_ and body position in RCTs comparing LPEEP vs MPEEP.**

Data are presented as effect sizes (Cohen’s d) and 95% confidence intervals. Each bubble represents one randomized controlled trial. The circle size represents the sample size of the trial. 1- Trendelenburg, 2-Reverse Trendelenburg

**Abbreviations**: CI: confidence interval; PaO2: arterial partial pressure of oxygen; LPEEP – low positive end-expiratory pressure; MPEEP – moderate positive end-expiratory pressure.

Meta-regression for the influence of patient position during surgery on PaO_2_/FiO_2_ did not find significant relationship between patient position and PaO_2_/FiO_2_  in LPEEP vs HPEEP RCTs (the regression coefficient 0.59, R^2^ 0.00%, p=0.887), null hypothesis of no residual heterogeneity was not rejected (Qres =1.54, p=0.215(**Figure S50**).

**
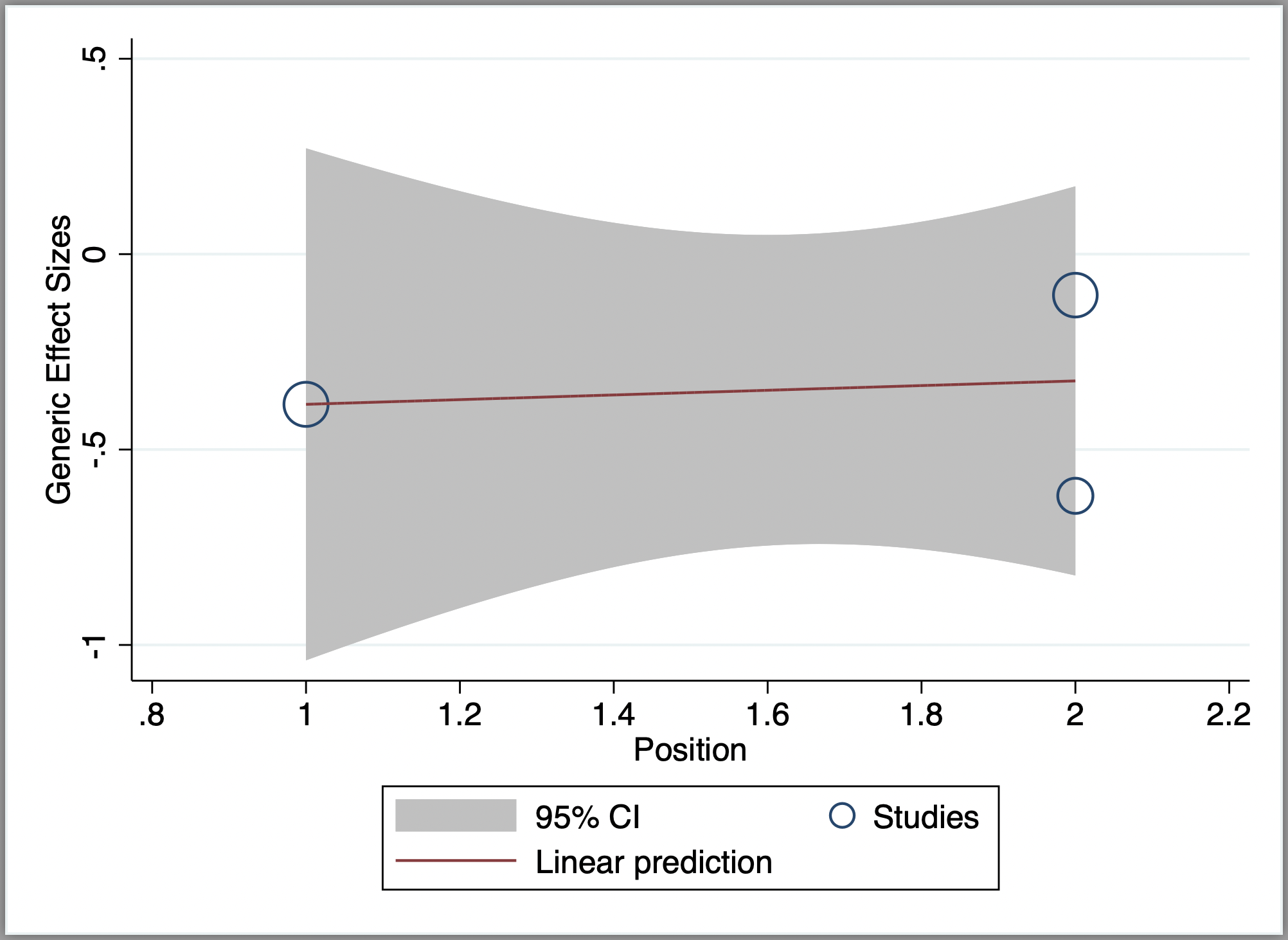
Figure S50. Relationship between PaO_2_/FiO_2_ and body position in RCTs comparing LPEEP vs HPEEP.**

Data are presented as effect sizes (Cohen’s d) and 95% confidence intervals. Each bubble represents one randomized controlled trial. The circle size represents the sample size of the trial. 1- Trendelenburg, 2-Reverse Trendelenburg

**Abbreviations**: CI: confidence interval; PaO2: arterial partial pressure of oxygen; LPEEP – low positive end-expiratory pressure; HPEEP – high positive end-expiratory pressure.

**Dynamic compliance (Cdyn)**

Meta-regression for the influence of body position during surgery on Cdyn did not find significant relationship between tidal volume and Cdyn in LPEEP vs MPEEP RCTs (the regression coefficient -0.36, R^2^ 0.00%, p=0.696), but found significant variation of the true effect (Qres = 65.44, p<0.0001)(**Figure S51**).

**
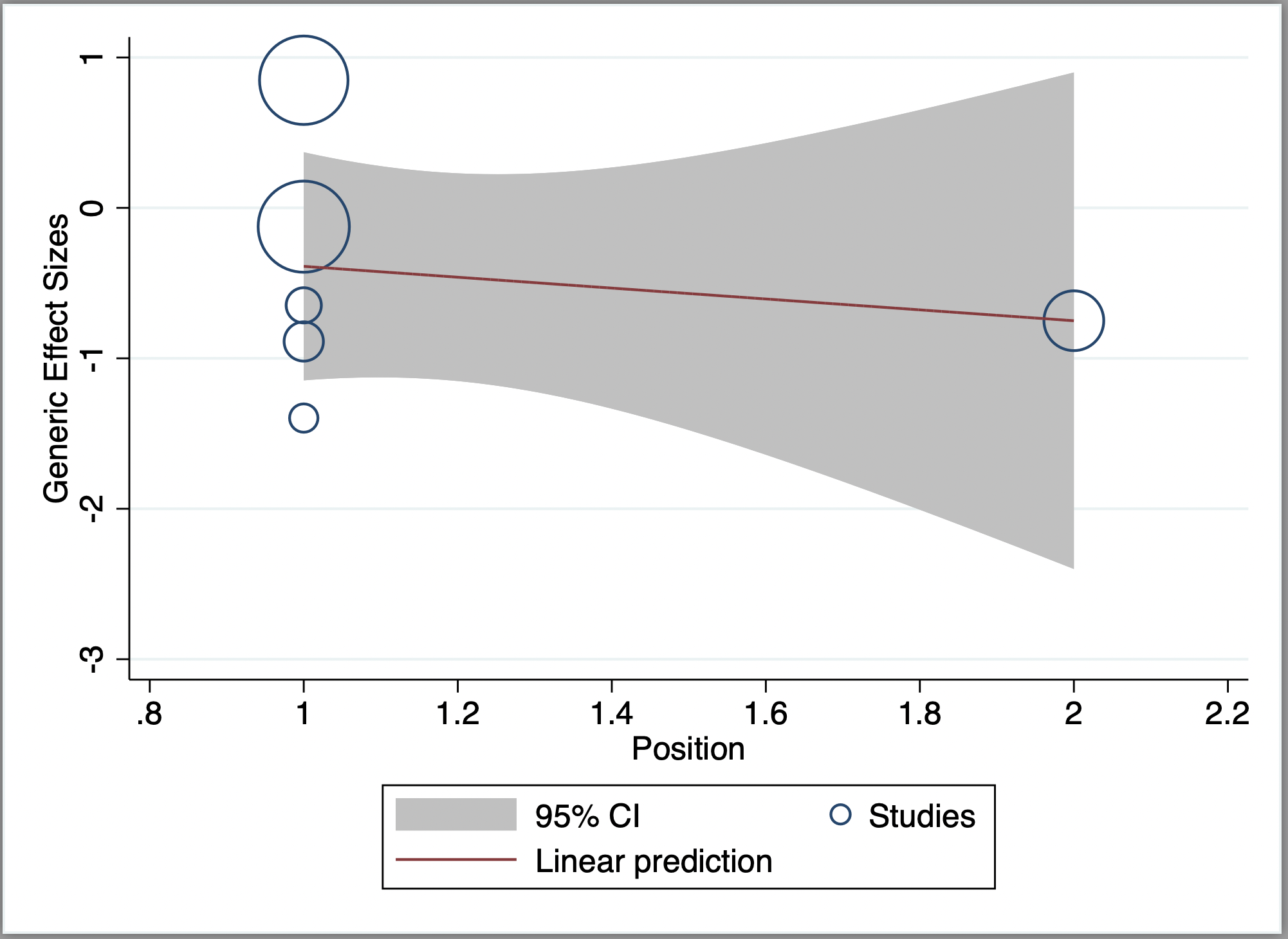
Figure S51. Relationship between Cdyn and body position during surgery in RCTs comparing LPEEP vs MPEEP.**

Data are presented as effect sizes (Cohen’s d) and 95% confidence intervals. Each bubble represents one randomized controlled trial. The circle size represents the sample size of the trial. 1- Trendelenburg, 2-Reverse Trendelenburg

**Abbreviations**: CI: confidence interval; Cdyn: dynamic compliance of respiratory system; LPEEP – low positive end-expiratory pressure; MPEEP – moderate positive end-expiratory pressure.

Meta-regression for the influence of body position during surgery on Cdyn found significant decrease in Cdyn in patients in Trendelenburg position in LPEEP vs HPEEP RCTs (the regression coefficient 1.34, R^2^100.00%, p<0.0001), null hypothesis of no residual heterogeneity was not rejected (Qres = 2.00, p=0.367 (**Figure S52**).

**
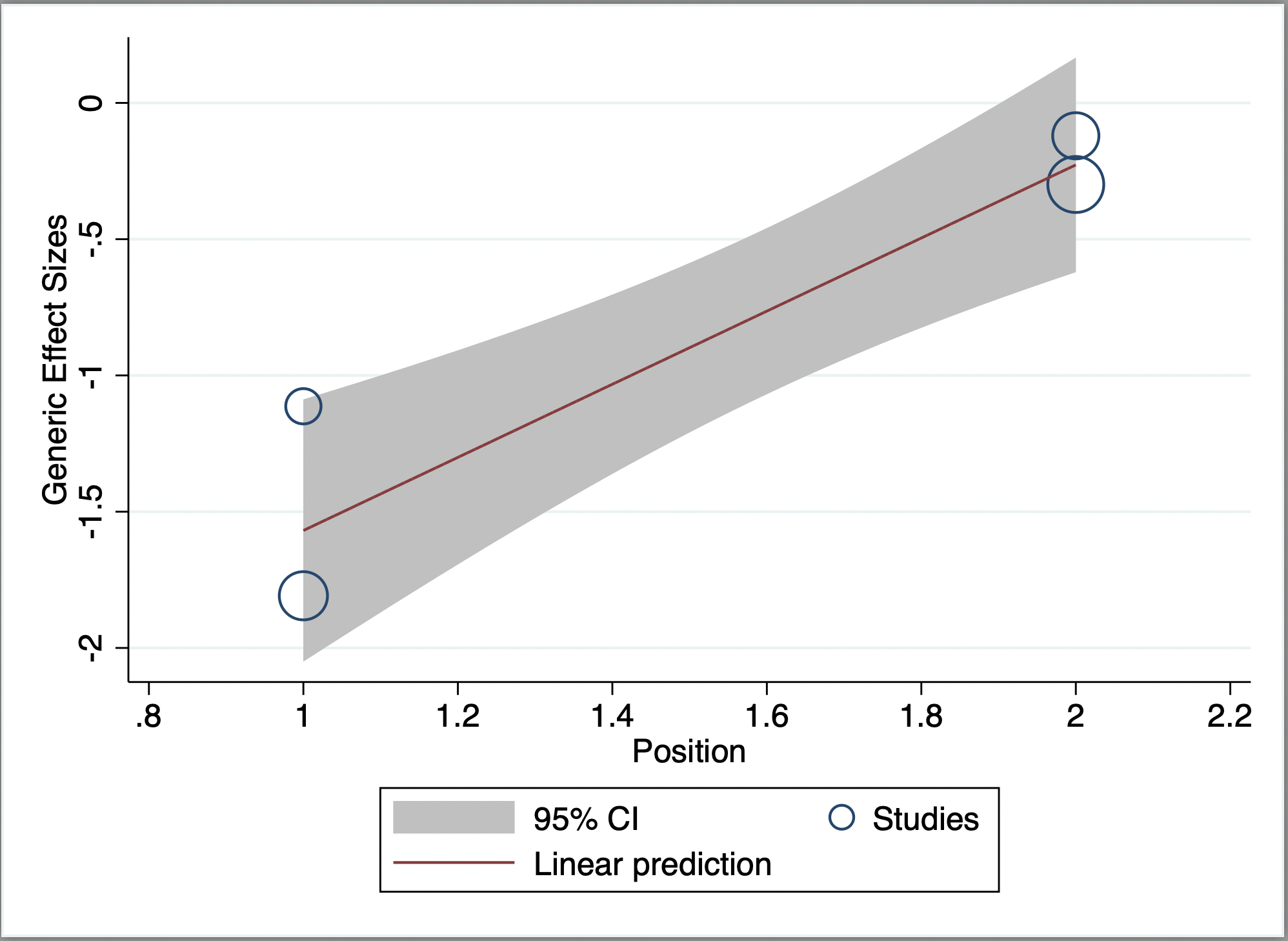
Figure S52. Relationship between Cdyn and body position during surgery in RCTs comparing LPEEP vs HPEEP.**

Data are presented as effect sizes (Cohen’s d) and 95% confidence intervals. Each bubble represents one randomized controlled trial. The circle size represents the sample size of the trial. 1- Trendelenburg, 2-Reverse Trendelenburg

**Abbreviations**: CI: confidence interval; Cdyn: dynamic compliance of respiratory system; LPEEP – low positive end-expiratory pressure; HPEEP – high positive end-expiratory pressure

**Mean arterial pressure (MAP) and the heart rate (HR)**

Meta-regression for the influence of body position during surgery on MAP did not find significant changes in MAP in LPEEP vs MPEEP RCTs (the regression coefficient 0.25, R^2^ 0.00%, p=0.539), but found significant variation of the true effect (Qres = 10.48, p=0.062)(**Figure S53**).

**
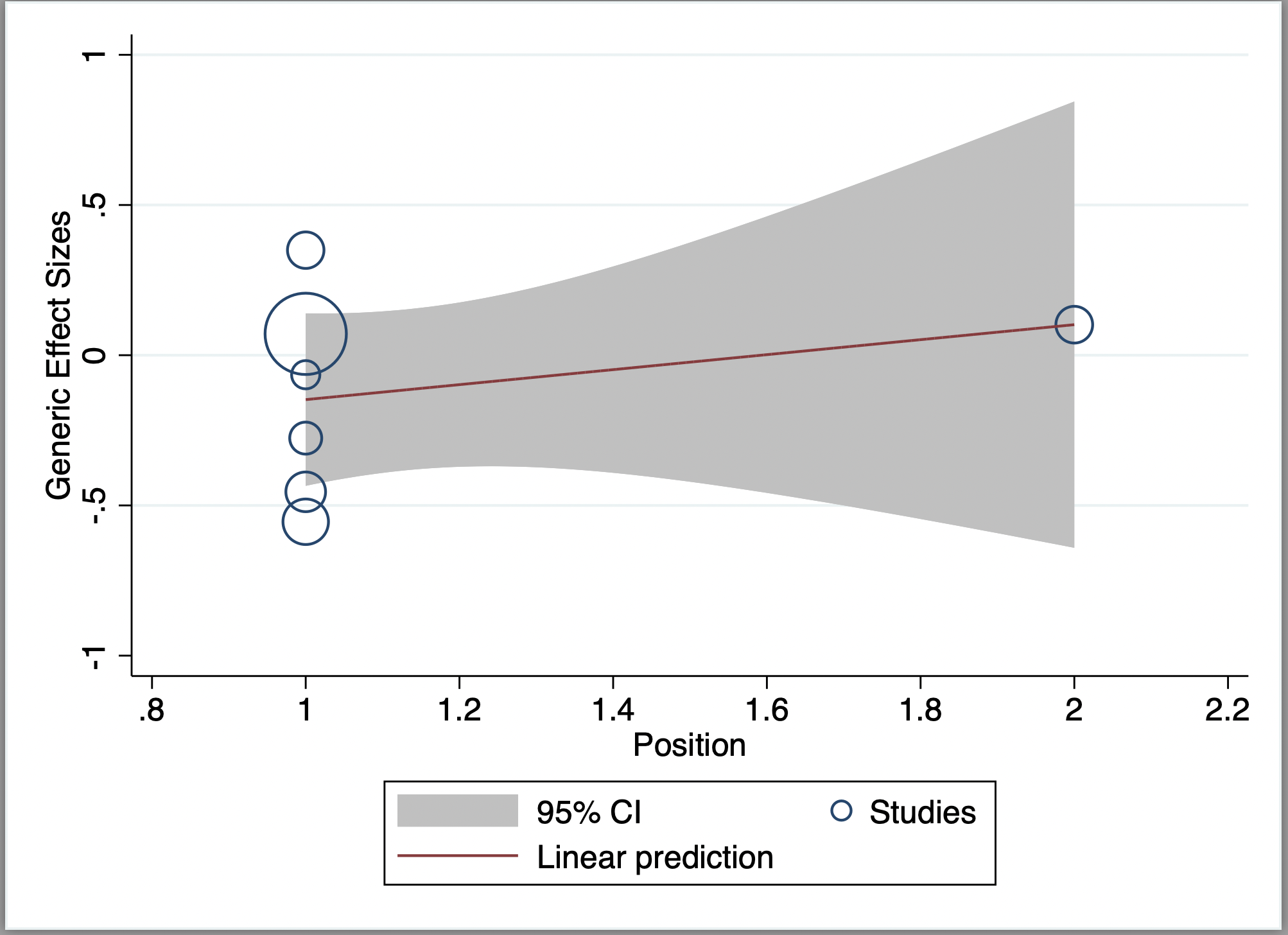
Figure S53. Relationship between MAP and body position during surgery in RCTs comparing LPEEP vs MPEEP.**

Data are presented as effect sizes (Cohen’s d) and 95% confidence intervals. Each bubble represents one randomized controlled trial. The circle size represents the sample size of the trial. 1- Trendelenburg, 2-Reverse Trendelenburg

**Abbreviations**: CI: confidence interval; MAP: mean arterial pressure; LPEEP – low positive end-expiratory pressure; MPEEP – moderate positive end-expiratory pressure.

Meta-regression for the influence of body position during surgery on MAP did not find significant changes in MAP in MPEEP vs HPEEP RCTs (the regression coefficient 0.29, R^2^ 0.00%, p=0.960), but found significant variation of the true effect (Qres = 6.01, p=0.049)(**Figure S54**).

**
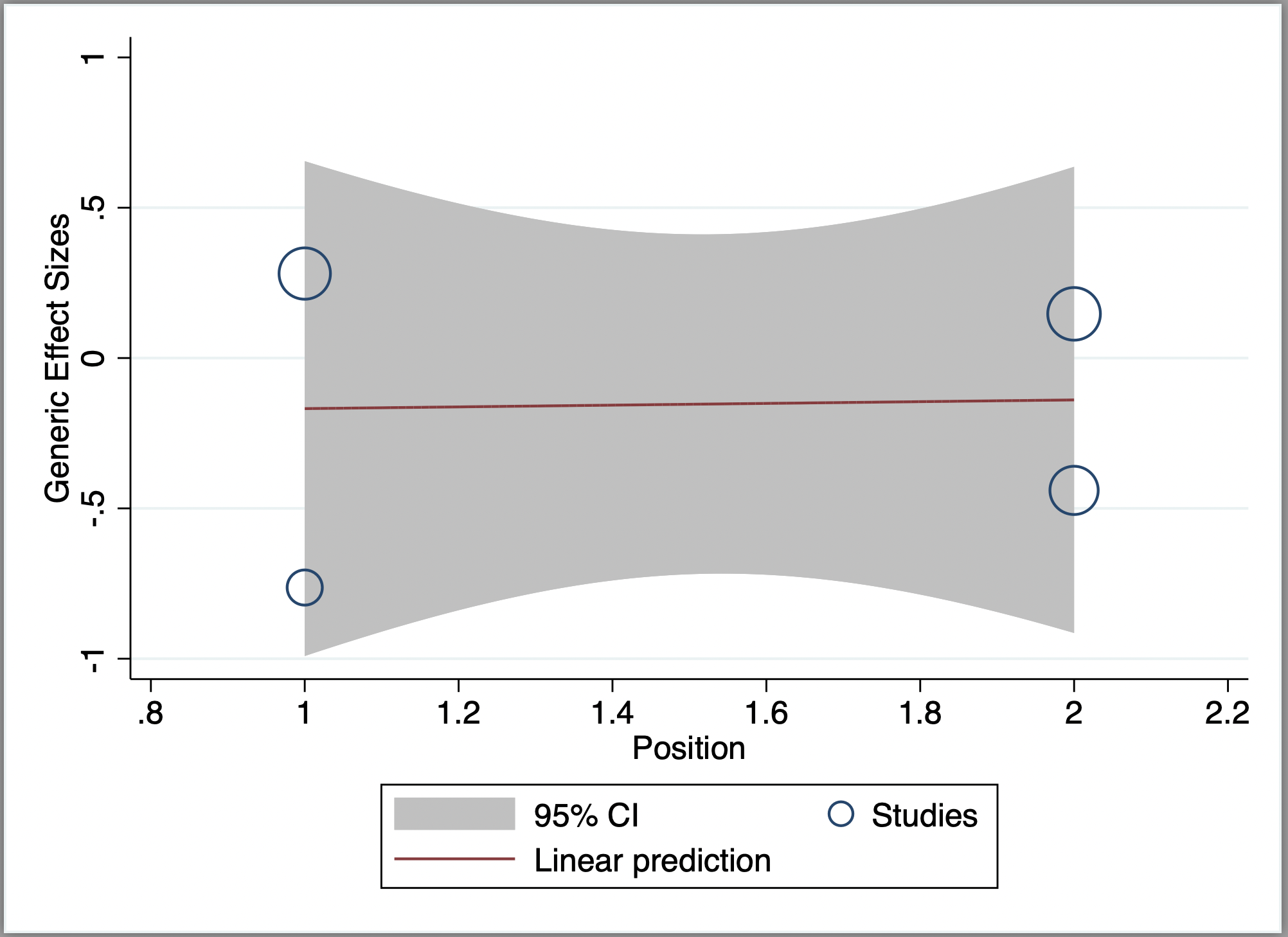
Figure S54. Relationship between MAP and body position during surgery in RCTs comparing MPEEP vs HPEEP.**

Data are presented as effect sizes (Cohen’s d) and 95% confidence intervals. Each bubble represents one randomized controlled trial. The circle size represents the sample size of the trial. 1- Trendelenburg, 2-Reverse Trendelenburg

**Abbreviations**: CI: confidence interval; MAP: mean arterial pressure; MPEEP – moderate positive end-expiratory pressure; HPEEP – high positive end-expiratory pressure.

Meta-regression for the influence of body position during surgery on HR did not find significant changes in HR in LPEEP vs MPEEP RCTs (the regression coefficient 0.53, R^2^ 53.59%, p=0.857), null hypothesis of no residual heterogeneity was not rejected (Qres = 3.26, p=0.661 (**Figure S55**).

**
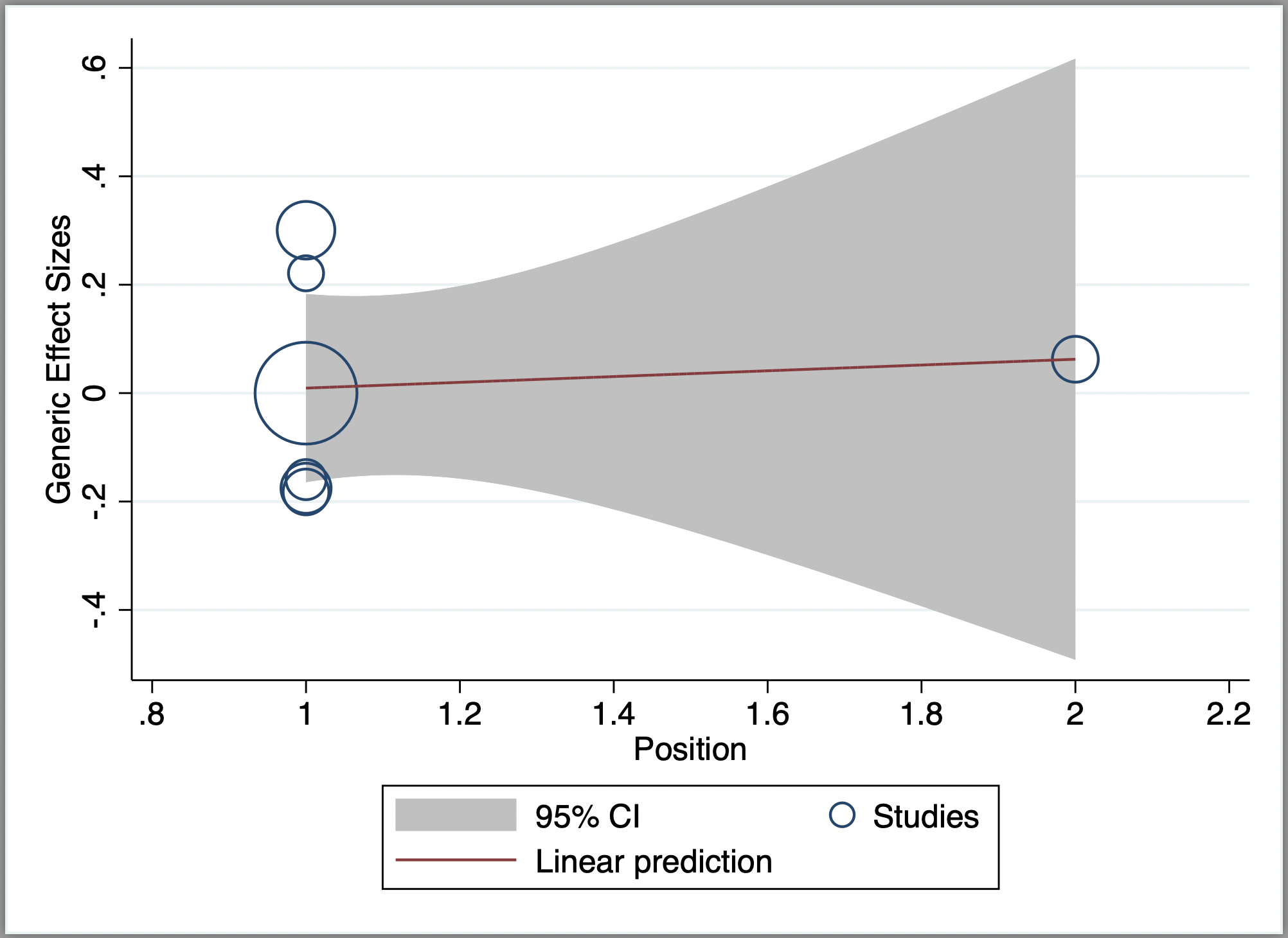
**

**Figure S55. Relationship between HR and body position during surgery in RCTs comparing LPEEP vs MPEEP.**

Data are presented as effect sizes (Cohen’s d) and 95% confidence intervals. Each bubble represents one randomized controlled trial. The circle size represents the sample size of the trial. 1- Trendelenburg, 2-Reverse Trendelenburg

**Abbreviations**: CI: confidence interval; HR: heart rate; LPEEP – low positive end-expiratory pressure; MPEEP – moderate positive end-expiratory pressure.
