# Supplementary material for: Impact of a positive end-expiratory pressure strategy on oxygenation, respiratory compliance, and hemodynamics during laparoscopic surgery in non-obese patients: a systematic review and meta-analysis of randomized controlled trials
Source: BMC Anesthesiol. 2023 Nov 11;23:371. doi: 10.1186/s12871-023-02337-0 (PMC10638810; doi:10.1186/s12871-023-02337-0)
Supplement: Supplementary file 2 — Supplementary Material 2 [file 12871_2023_2337_MOESM2_ESM.docx]

**Online Data Supplement** **2**

**Table S1. The risk of bias judgments in the included studies assessed by the Risk of Bias 2.0 (RoB 2) tool.**

| ID1 | **Study ID** | Kwak HJ et al (2012) | **Assessor** | ZK |
| --- | --- | --- | --- | --- |
| Kwak HJ et al (2012) | **Aim** | assignment to intervention (the 'intention-to-treat' effect) |  |  |
| Intervention | **Comparator** | Control | **Source** | Journal article(s) |
| Effectiveness | **Results** |  | **Weight** | 1 |
| **Signalling question** | | | **Response** | **Comments** |
| 1.1 Was the allocation sequence random? | | | Y |  |
| 1.2 Was the allocation sequence concealed until participants were enrolled and assigned to interventions? | | | Y |  |
| 1.3 Did baseline differences between intervention groups suggest a problem with the randomization process? | | | N |  |
| **Risk of bias judgement** | | | **Low** |  |
| 2.1.Were participants aware of their assigned intervention during the trial? | | | N |  |
| 2.2.Were carers and people delivering the interventions aware of participants' assigned intervention during the trial? | | | PY |  |
| 2.3. If Y/PY/NI to 2.1 or 2.2: Were there deviations from the intended intervention that arose because of the experimental context? | | | N |  |
| 2.4 If Y/PY to 2.3: Were these deviations likely to have affected the outcome? | | | NA |  |
| 2.5. If Y/PY/NI to 2.4: Were these deviations from intended intervention balanced between groups? | | | NA |  |
| 2.6 Was an appropriate analysis used to estimate the effect of assignment to intervention? | | | Y |  |
| 2.7 If N/PN/NI to 2.6: Was there potential for a substantial impact (on the result) of the failure to analyse participants in the group to which they were randomized? | | | NA |  |
| **Risk of bias judgement** | | | **Low** |  |
| 3.1 Were data for this outcome available for all, or nearly all, participants randomized? | | | Y |  |
| 3.2 If N/PN/NI to 3.1: Is there evidence that result was not biased by missing outcome data? | | | NA |  |
| 3.3 If N/PN to 3.2: Could missingness in the outcome depend on its true value? | | | NA |  |
| 3.4 If Y/PY/NI to 3.3: Is it likely that missingness in the outcome depended on its true value? | | | NA |  |
| **Risk of bias judgement** | | | **Low** |  |
| 4.1 Was the method of measuring the outcome inappropriate? | | | N |  |
| 4.2 Could measurement or ascertainment of the outcome have differed between intervention groups? | | | Y |  |
| 4.3 Were outcome assessors aware of the intervention received by study participants? | | | NA |  |
| 4.4 If Y/PY/NI to 4.3: Could assessment of the outcome have been influenced by knowledge of intervention received? | | | NA |  |
| 4.5 If Y/PY/NI to 4.4: Is it likely that assessment of the outcome was influenced by knowledge of intervention received? | | | NA |  |
| **Risk of bias judgement** | | | **High** |  |
| 5.1 Were the data that produced this result analysed in accordance with a pre-specified analysis plan that was finalized before unblinded outcome data were available for analysis? | | | N |  |
| 5.2 ... multiple eligible outcome measurements (e.g. scales, definitions, time points) within the outcome domain? | | | Y |  |
| 5.3 ... multiple eligible analyses of the data? | | | Y |  |
| **Risk of bias judgement** | | | **High** |  |
| **Risk of bias judgement** | | | **Some concerns** |  |
|  |  |  |  |  |
|  |  |  |  |  |
| ID2 | **Study ID** | Russo A et al (2013) | **Assessor** | ZK |
| Russo A et al (2013) | **Aim** | assignment to intervention (the 'intention-to-treat' effect) |  |  |
| Intervention | **Comparator** | control | **Source** | Journal article(s) |
| Effectiveness | **Results** |  | **Weight** | 1 |
| **Signalling question** | | | **Response** | **Comments** |
| 1.1 Was the allocation sequence random? | | | Y |  |
| 1.2 Was the allocation sequence concealed until participants were enrolled and assigned to interventions? | | | Y |  |
| 1.3 Did baseline differences between intervention groups suggest a problem with the randomization process? | | | N |  |
| **Risk of bias judgement** | | | **Low** |  |
| 2.1.Were participants aware of their assigned intervention during the trial? | | | N |  |
| 2.2.Were carers and people delivering the interventions aware of participants' assigned intervention during the trial? | | | PN |  |
| 2.3. If Y/PY/NI to 2.1 or 2.2: Were there deviations from the intended intervention that arose because of the experimental context? | | | NA |  |
| 2.4 If Y/PY to 2.3: Were these deviations likely to have affected the outcome? | | | NA |  |
| 2.5. If Y/PY/NI to 2.4: Were these deviations from intended intervention balanced between groups? | | | NA |  |
| 2.6 Was an appropriate analysis used to estimate the effect of assignment to intervention? | | | Y |  |
| 2.7 If N/PN/NI to 2.6: Was there potential for a substantial impact (on the result) of the failure to analyse participants in the group to which they were randomized? | | | NA |  |
| **Risk of bias judgement** | | | **Low** |  |
| 3.1 Were data for this outcome available for all, or nearly all, participants randomized? | | | Y |  |
| 3.2 If N/PN/NI to 3.1: Is there evidence that result was not biased by missing outcome data? | | | NA |  |
| 3.3 If N/PN to 3.2: Could missingness in the outcome depend on its true value? | | | NA |  |
| 3.4 If Y/PY/NI to 3.3: Is it likely that missingness in the outcome depended on its true value? | | | NA |  |
| **Risk of bias judgement** | | | **Low** |  |
| 4.1 Was the method of measuring the outcome inappropriate? | | | N |  |
| 4.2 Could measurement or ascertainment of the outcome have differed between intervention groups? | | | N |  |
| 4.3 Were outcome assessors aware of the intervention received by study participants? | | | PY |  |
| 4.4 If Y/PY/NI to 4.3: Could assessment of the outcome have been influenced by knowledge of intervention received? | | | N |  |
| 4.5 If Y/PY/NI to 4.4: Is it likely that assessment of the outcome was influenced by knowledge of intervention received? | | | NA |  |
| **Risk of bias judgement** | | | **Low** |  |
| 5.1 Were the data that produced this result analysed in accordance with a pre-specified analysis plan that was finalized before unblinded outcome data were available for analysis? | | | PN |  |
| 5.2 ... multiple eligible outcome measurements (e.g. scales, definitions, time points) within the outcome domain? | | | PN |  |
| 5.3 ... multiple eligible analyses of the data? | | | NI |  |
| **Risk of bias judgement** | | | **Some concerns** |  |
| **Risk of bias judgement** | | | **Low** |  |
|  |  |  |  |  |
|  |  |  |  |  |
| ID3 | **Study ID** | Baki ED et al (2014) | **Assessor** | ZK |
| Baki ED et al (2014) | **Aim** | assignment to intervention (the 'intention-to-treat' effect) |  |  |
| Intervention | **Comparator** | control | **Source** | Journal article(s) |
| Effectiveness | **Results** |  | **Weight** | 1 |
| **Signalling question** | | | **Response** | **Comments** |
| 1.1 Was the allocation sequence random? | | | Y |  |
| 1.2 Was the allocation sequence concealed until participants were enrolled and assigned to interventions? | | | Y |  |
| 1.3 Did baseline differences between intervention groups suggest a problem with the randomization process? | | | N |  |
| **Risk of bias judgement** | | | **Low** |  |
| 2.1.Were participants aware of their assigned intervention during the trial? | | | Y |  |
| 2.2.Were carers and people delivering the interventions aware of participants' assigned intervention during the trial? | | | PY |  |
| 2.3. If Y/PY/NI to 2.1 or 2.2: Were there deviations from the intended intervention that arose because of the experimental context? | | | N |  |
| 2.4 If Y/PY to 2.3: Were these deviations likely to have affected the outcome? | | | NA |  |
| 2.5. If Y/PY/NI to 2.4: Were these deviations from intended intervention balanced between groups? | | | NA |  |
| 2.6 Was an appropriate analysis used to estimate the effect of assignment to intervention? | | | Y |  |
| 2.7 If N/PN/NI to 2.6: Was there potential for a substantial impact (on the result) of the failure to analyse participants in the group to which they were randomized? | | | NA |  |
| **Risk of bias judgement** | | | **Low** |  |
| 3.1 Were data for this outcome available for all, or nearly all, participants randomized? | | | Y |  |
| 3.2 If N/PN/NI to 3.1: Is there evidence that result was not biased by missing outcome data? | | | NA |  |
| 3.3 If N/PN to 3.2: Could missingness in the outcome depend on its true value? | | | NA |  |
| 3.4 If Y/PY/NI to 3.3: Is it likely that missingness in the outcome depended on its true value? | | | NA |  |
| **Risk of bias judgement** | | | **Low** |  |
| 4.1 Was the method of measuring the outcome inappropriate? | | | N |  |
| 4.2 Could measurement or ascertainment of the outcome have differed between intervention groups? | | | N |  |
| 4.3 Were outcome assessors aware of the intervention received by study participants? | | | PY |  |
| 4.4 If Y/PY/NI to 4.3: Could assessment of the outcome have been influenced by knowledge of intervention received? | | | N |  |
| 4.5 If Y/PY/NI to 4.4: Is it likely that assessment of the outcome was influenced by knowledge of intervention received? | | | NA |  |
| **Risk of bias judgement** | | | **Low** |  |
| 5.1 Were the data that produced this result analysed in accordance with a pre-specified analysis plan that was finalized before unblinded outcome data were available for analysis? | | | Y |  |
| 5.2 ... multiple eligible outcome measurements (e.g. scales, definitions, time points) within the outcome domain? | | | NI |  |
| 5.3 ... multiple eligible analyses of the data? | | | NI |  |
| **Risk of bias judgement** | | | **Some concerns** |  |
| **Risk of bias judgement** | | | **Low** |  |
|  |  |  |  |  |
|  |  |  |  |  |
| ID4 | **Study ID** | Ela Y et al (2014) | **Assessor** | ZK |
| Ela Y et al (2014) | **Aim** | assignment to intervention (the 'intention-to-treat' effect) |  |  |
| Intervention | **Comparator** | control | **Source** | Journal article(s) |
| Effectiveness | **Results** |  | **Weight** | 1 |
| **Signalling question** | | | **Response** | **Comments** |
| 1.1 Was the allocation sequence random? | | | Y |  |
| 1.2 Was the allocation sequence concealed until participants were enrolled and assigned to interventions? | | | Y |  |
| 1.3 Did baseline differences between intervention groups suggest a problem with the randomization process? | | | N |  |
| **Risk of bias judgement** | | | **Low** |  |
| 2.1.Were participants aware of their assigned intervention during the trial? | | | N |  |
| 2.2.Were carers and people delivering the interventions aware of participants' assigned intervention during the trial? | | | N |  |
| 2.3. If Y/PY/NI to 2.1 or 2.2: Were there deviations from the intended intervention that arose because of the experimental context? | | | NA |  |
| 2.4 If Y/PY to 2.3: Were these deviations likely to have affected the outcome? | | | NA |  |
| 2.5. If Y/PY/NI to 2.4: Were these deviations from intended intervention balanced between groups? | | | NA |  |
| 2.6 Was an appropriate analysis used to estimate the effect of assignment to intervention? | | | Y |  |
| 2.7 If N/PN/NI to 2.6: Was there potential for a substantial impact (on the result) of the failure to analyse participants in the group to which they were randomized? | | | NA |  |
| **Risk of bias judgement** | | | **Low** |  |
| 3.1 Were data for this outcome available for all, or nearly all, participants randomized? | | | Y |  |
| 3.2 If N/PN/NI to 3.1: Is there evidence that result was not biased by missing outcome data? | | | NA |  |
| 3.3 If N/PN to 3.2: Could missingness in the outcome depend on its true value? | | | NA |  |
| 3.4 If Y/PY/NI to 3.3: Is it likely that missingness in the outcome depended on its true value? | | | NA |  |
| **Risk of bias judgement** | | | **Low** |  |
| 4.1 Was the method of measuring the outcome inappropriate? | | | N |  |
| 4.2 Could measurement or ascertainment of the outcome have differed between intervention groups? | | | N |  |
| 4.3 Were outcome assessors aware of the intervention received by study participants? | | | N |  |
| 4.4 If Y/PY/NI to 4.3: Could assessment of the outcome have been influenced by knowledge of intervention received? | | | NA |  |
| 4.5 If Y/PY/NI to 4.4: Is it likely that assessment of the outcome was influenced by knowledge of intervention received? | | | NA |  |
| **Risk of bias judgement** | | | **Low** |  |
| 5.1 Were the data that produced this result analysed in accordance with a pre-specified analysis plan that was finalized before unblinded outcome data were available for analysis? | | | Y |  |
| 5.2 ... multiple eligible outcome measurements (e.g. scales, definitions, time points) within the outcome domain? | | | N |  |
| 5.3 ... multiple eligible analyses of the data? | | | N |  |
| **Risk of bias judgement** | | | **Low** |  |
| **Risk of bias judgement** | | | **Low** |  |
|  |  |  |  |  |
|  |  |  |  |  |
| ID5 | **Study ID** | Kundra P et al (2014) | **Assessor** | ZK |
| Kundra P et al (2014) | **Aim** | assignment to intervention (the 'intention-to-treat' effect) |  |  |
| Intervention | **Comparator** | Control | **Source** | Journal article(s) |
| Effectiveness | **Results** |  | **Weight** | 1 |
| **Signalling question** | | | **Response** | **Comments** |
| 1.1 Was the allocation sequence random? | | | Y |  |
| 1.2 Was the allocation sequence concealed until participants were enrolled and assigned to interventions? | | | Y |  |
| 1.3 Did baseline differences between intervention groups suggest a problem with the randomization process? | | | N |  |
| **Risk of bias judgement** | | | **Low** |  |
| 2.1.Were participants aware of their assigned intervention during the trial? | | | PY |  |
| 2.2.Were carers and people delivering the interventions aware of participants' assigned intervention during the trial? | | | PY |  |
| 2.3. If Y/PY/NI to 2.1 or 2.2: Were there deviations from the intended intervention that arose because of the experimental context? | | | N |  |
| 2.4 If Y/PY to 2.3: Were these deviations likely to have affected the outcome? | | | NA |  |
| 2.5. If Y/PY/NI to 2.4: Were these deviations from intended intervention balanced between groups? | | | NA |  |
| 2.6 Was an appropriate analysis used to estimate the effect of assignment to intervention? | | | Y |  |
| 2.7 If N/PN/NI to 2.6: Was there potential for a substantial impact (on the result) of the failure to analyse participants in the group to which they were randomized? | | | NA |  |
| **Risk of bias judgement** | | | **Low** |  |
| 3.1 Were data for this outcome available for all, or nearly all, participants randomized? | | | PY |  |
| 3.2 If N/PN/NI to 3.1: Is there evidence that result was not biased by missing outcome data? | | | NA |  |
| 3.3 If N/PN to 3.2: Could missingness in the outcome depend on its true value? | | | NA |  |
| 3.4 If Y/PY/NI to 3.3: Is it likely that missingness in the outcome depended on its true value? | | | NA |  |
| **Risk of bias judgement** | | | **Low** |  |
| 4.1 Was the method of measuring the outcome inappropriate? | | | N |  |
| 4.2 Could measurement or ascertainment of the outcome have differed between intervention groups? | | | Y |  |
| 4.3 Were outcome assessors aware of the intervention received by study participants? | | | NA |  |
| 4.4 If Y/PY/NI to 4.3: Could assessment of the outcome have been influenced by knowledge of intervention received? | | | NA |  |
| 4.5 If Y/PY/NI to 4.4: Is it likely that assessment of the outcome was influenced by knowledge of intervention received? | | | NA |  |
| **Risk of bias judgement** | | | **High** |  |
| 5.1 Were the data that produced this result analysed in accordance with a pre-specified analysis plan that was finalized before unblinded outcome data were available for analysis? | | | NI |  |
| 5.2 ... multiple eligible outcome measurements (e.g. scales, definitions, time points) within the outcome domain? | | | N |  |
| 5.3 ... multiple eligible analyses of the data? | | | N |  |
| **Risk of bias judgement** | | | **Some concerns** |  |
| **Risk of bias judgement** | | | **High** |  |
|  |  |  |  |  |
|  |  |  |  |  |
| ID6 | **Study ID** | Karabayirli S et al (2016) | **Assessor** | ZK |
| Karabayirli S et al (2016) | **Aim** | assignment to intervention (the 'intention-to-treat' effect) |  |  |
| Intervention | **Comparator** | Control | **Source** | Journal article(s) |
| Effectiveness | **Results** |  | **Weight** | 1 |
| **Signalling question** | | | **Response** | **Comments** |
| 1.1 Was the allocation sequence random? | | | Y |  |
| 1.2 Was the allocation sequence concealed until participants were enrolled and assigned to interventions? | | | Y |  |
| 1.3 Did baseline differences between intervention groups suggest a problem with the randomization process? | | | N |  |
| **Risk of bias judgement** | | | **Low** |  |
| 2.1.Were participants aware of their assigned intervention during the trial? | | | PN |  |
| 2.2.Were carers and people delivering the interventions aware of participants' assigned intervention during the trial? | | | PN |  |
| 2.3. If Y/PY/NI to 2.1 or 2.2: Were there deviations from the intended intervention that arose because of the experimental context? | | | NA |  |
| 2.4 If Y/PY to 2.3: Were these deviations likely to have affected the outcome? | | | NA |  |
| 2.5. If Y/PY/NI to 2.4: Were these deviations from intended intervention balanced between groups? | | | NA |  |
| 2.6 Was an appropriate analysis used to estimate the effect of assignment to intervention? | | | Y |  |
| 2.7 If N/PN/NI to 2.6: Was there potential for a substantial impact (on the result) of the failure to analyse participants in the group to which they were randomized? | | | NA |  |
| **Risk of bias judgement** | | | **Low** |  |
| 3.1 Were data for this outcome available for all, or nearly all, participants randomized? | | | PN |  |
| 3.2 If N/PN/NI to 3.1: Is there evidence that result was not biased by missing outcome data? | | | PY |  |
| 3.3 If N/PN to 3.2: Could missingness in the outcome depend on its true value? | | | NA |  |
| 3.4 If Y/PY/NI to 3.3: Is it likely that missingness in the outcome depended on its true value? | | | NA |  |
| **Risk of bias judgement** | | | **Low** |  |
| 4.1 Was the method of measuring the outcome inappropriate? | | | NI |  |
| 4.2 Could measurement or ascertainment of the outcome have differed between intervention groups? | | | N |  |
| 4.3 Were outcome assessors aware of the intervention received by study participants? | | | NI |  |
| 4.4 If Y/PY/NI to 4.3: Could assessment of the outcome have been influenced by knowledge of intervention received? | | | N |  |
| 4.5 If Y/PY/NI to 4.4: Is it likely that assessment of the outcome was influenced by knowledge of intervention received? | | | NA |  |
| **Risk of bias judgement** | | | **Low** |  |
| 5.1 Were the data that produced this result analysed in accordance with a pre-specified analysis plan that was finalized before unblinded outcome data were available for analysis? | | | NI |  |
| 5.2 ... multiple eligible outcome measurements (e.g. scales, definitions, time points) within the outcome domain? | | | N |  |
| 5.3 ... multiple eligible analyses of the data? | | | Y |  |
| **Risk of bias judgement** | | | **High** |  |
| **Risk of bias judgement** | | | **Low** |  |
|  |  |  |  |  |
|  |  |  |  |  |
| ID7 | **Study ID** | He X et al (2016) | **Assessor** | ZK |
| He X et al (2016) | **Aim** | assignment to intervention (the 'intention-to-treat' effect) |  |  |
| Intervention | **Comparator** | control | **Source** | Journal article(s) |
| Effectiveness | **Results** |  | **Weight** | 1 |
| **Signalling question** | | | **Response** | **Comments** |
| 1.1 Was the allocation sequence random? | | | Y |  |
| 1.2 Was the allocation sequence concealed until participants were enrolled and assigned to interventions? | | | PY |  |
| 1.3 Did baseline differences between intervention groups suggest a problem with the randomization process? | | | N |  |
| **Risk of bias judgement** | | | **Low** |  |
| 2.1.Were participants aware of their assigned intervention during the trial? | | | NI |  |
| 2.2.Were carers and people delivering the interventions aware of participants' assigned intervention during the trial? | | | PY |  |
| 2.3. If Y/PY/NI to 2.1 or 2.2: Were there deviations from the intended intervention that arose because of the experimental context? | | | NI |  |
| 2.4 If Y/PY to 2.3: Were these deviations likely to have affected the outcome? | | | NA |  |
| 2.5. If Y/PY/NI to 2.4: Were these deviations from intended intervention balanced between groups? | | | NA |  |
| 2.6 Was an appropriate analysis used to estimate the effect of assignment to intervention? | | | Y |  |
| 2.7 If N/PN/NI to 2.6: Was there potential for a substantial impact (on the result) of the failure to analyse participants in the group to which they were randomized? | | | NA |  |
| **Risk of bias judgement** | | | **Some concerns** |  |
| 3.1 Were data for this outcome available for all, or nearly all, participants randomized? | | | Y |  |
| 3.2 If N/PN/NI to 3.1: Is there evidence that result was not biased by missing outcome data? | | | NA |  |
| 3.3 If N/PN to 3.2: Could missingness in the outcome depend on its true value? | | | NA |  |
| 3.4 If Y/PY/NI to 3.3: Is it likely that missingness in the outcome depended on its true value? | | | NA |  |
| **Risk of bias judgement** | | | **Low** |  |
| 4.1 Was the method of measuring the outcome inappropriate? | | | N |  |
| 4.2 Could measurement or ascertainment of the outcome have differed between intervention groups? | | | NI |  |
| 4.3 Were outcome assessors aware of the intervention received by study participants? | | | N |  |
| 4.4 If Y/PY/NI to 4.3: Could assessment of the outcome have been influenced by knowledge of intervention received? | | | NA |  |
| 4.5 If Y/PY/NI to 4.4: Is it likely that assessment of the outcome was influenced by knowledge of intervention received? | | | NA |  |
| **Risk of bias judgement** | | | **Some concerns** |  |
| 5.1 Were the data that produced this result analysed in accordance with a pre-specified analysis plan that was finalized before unblinded outcome data were available for analysis? | | | Y |  |
| 5.2 ... multiple eligible outcome measurements (e.g. scales, definitions, time points) within the outcome domain? | | | N |  |
| 5.3 ... multiple eligible analyses of the data? | | | N |  |
| **Risk of bias judgement** | | | **Low** |  |
| **Risk of bias judgement** | | | **Some concerns** |  |
|  |  |  |  |  |
|  |  |  |  |  |
| ID8 | **Study ID** | Sen O et al (2017) | **Assessor** | ZK |
| Sen O et al (2017) | **Aim** | assignment to intervention (the 'intention-to-treat' effect) |  |  |
| Intervention | **Comparator** | control | **Source** | Journal article(s) |
| Effectiveness | **Results** |  | **Weight** | 1 |
| **Signalling question** | | | **Response** | **Comments** |
| 1.1 Was the allocation sequence random? | | | Y |  |
| 1.2 Was the allocation sequence concealed until participants were enrolled and assigned to interventions? | | | Y |  |
| 1.3 Did baseline differences between intervention groups suggest a problem with the randomization process? | | | N |  |
| **Risk of bias judgement** | | | **Low** |  |
| 2.1.Were participants aware of their assigned intervention during the trial? | | | PY |  |
| 2.2.Were carers and people delivering the interventions aware of participants' assigned intervention during the trial? | | | NI |  |
| 2.3. If Y/PY/NI to 2.1 or 2.2: Were there deviations from the intended intervention that arose because of the experimental context? | | | N |  |
| 2.4 If Y/PY to 2.3: Were these deviations likely to have affected the outcome? | | | NA |  |
| 2.5. If Y/PY/NI to 2.4: Were these deviations from intended intervention balanced between groups? | | | NA |  |
| 2.6 Was an appropriate analysis used to estimate the effect of assignment to intervention? | | | Y |  |
| 2.7 If N/PN/NI to 2.6: Was there potential for a substantial impact (on the result) of the failure to analyse participants in the group to which they were randomized? | | | NA |  |
| **Risk of bias judgement** | | | **Low** |  |
| 3.1 Were data for this outcome available for all, or nearly all, participants randomized? | | | N |  |
| 3.2 If N/PN/NI to 3.1: Is there evidence that result was not biased by missing outcome data? | | | PN |  |
| 3.3 If N/PN to 3.2: Could missingness in the outcome depend on its true value? | | | N |  |
| 3.4 If Y/PY/NI to 3.3: Is it likely that missingness in the outcome depended on its true value? | | | NA |  |
| **Risk of bias judgement** | | | **Low** |  |
| 4.1 Was the method of measuring the outcome inappropriate? | | | N |  |
| 4.2 Could measurement or ascertainment of the outcome have differed between intervention groups? | | | N |  |
| 4.3 Were outcome assessors aware of the intervention received by study participants? | | | N |  |
| 4.4 If Y/PY/NI to 4.3: Could assessment of the outcome have been influenced by knowledge of intervention received? | | | NA |  |
| 4.5 If Y/PY/NI to 4.4: Is it likely that assessment of the outcome was influenced by knowledge of intervention received? | | | NA |  |
| **Risk of bias judgement** | | | **Low** |  |
| 5.1 Were the data that produced this result analysed in accordance with a pre-specified analysis plan that was finalized before unblinded outcome data were available for analysis? | | | Y |  |
| 5.2 ... multiple eligible outcome measurements (e.g. scales, definitions, time points) within the outcome domain? | | | Y |  |
| 5.3 ... multiple eligible analyses of the data? | | | Y |  |
| **Risk of bias judgement** | | | **High** |  |
| **Risk of bias judgement** | | | **High** |  |
|  |  |  |  |  |
|  |  |  |  |  |
| ID9 | **Study ID** | Chin JH et al (2019) | **Assessor** | ZK |
| Chin JH et al (2017) | **Aim** | assignment to intervention (the 'intention-to-treat' effect) |  |  |
| Intervention | **Comparator** | control | **Source** | Journal article(s) |
| Effectiveness | **Results** |  | **Weight** | 1 |
| **Signalling question** | | | **Response** | **Comments** |
| 1.1 Was the allocation sequence random? | | | Y |  |
| 1.2 Was the allocation sequence concealed until participants were enrolled and assigned to interventions? | | | Y |  |
| 1.3 Did baseline differences between intervention groups suggest a problem with the randomization process? | | | N |  |
| **Risk of bias judgement** | | | **Low** |  |
| 2.1.Were participants aware of their assigned intervention during the trial? | | | PY |  |
| 2.2.Were carers and people delivering the interventions aware of participants' assigned intervention during the trial? | | | Y |  |
| 2.3. If Y/PY/NI to 2.1 or 2.2: Were there deviations from the intended intervention that arose because of the experimental context? | | | N |  |
| 2.4 If Y/PY to 2.3: Were these deviations likely to have affected the outcome? | | | NA |  |
| 2.5. If Y/PY/NI to 2.4: Were these deviations from intended intervention balanced between groups? | | | NA |  |
| 2.6 Was an appropriate analysis used to estimate the effect of assignment to intervention? | | | Y |  |
| 2.7 If N/PN/NI to 2.6: Was there potential for a substantial impact (on the result) of the failure to analyse participants in the group to which they were randomized? | | | NA |  |
| **Risk of bias judgement** | | | **Low** |  |
| 3.1 Were data for this outcome available for all, or nearly all, participants randomized? | | | N |  |
| 3.2 If N/PN/NI to 3.1: Is there evidence that result was not biased by missing outcome data? | | | PN |  |
| 3.3 If N/PN to 3.2: Could missingness in the outcome depend on its true value? | | | N |  |
| 3.4 If Y/PY/NI to 3.3: Is it likely that missingness in the outcome depended on its true value? | | | NA |  |
| **Risk of bias judgement** | | | **Low** |  |
| 4.1 Was the method of measuring the outcome inappropriate? | | | N |  |
| 4.2 Could measurement or ascertainment of the outcome have differed between intervention groups? | | | N |  |
| 4.3 Were outcome assessors aware of the intervention received by study participants? | | | N |  |
| 4.4 If Y/PY/NI to 4.3: Could assessment of the outcome have been influenced by knowledge of intervention received? | | | NA |  |
| 4.5 If Y/PY/NI to 4.4: Is it likely that assessment of the outcome was influenced by knowledge of intervention received? | | | NA |  |
| **Risk of bias judgement** | | | **Low** |  |
| 5.1 Were the data that produced this result analysed in accordance with a pre-specified analysis plan that was finalized before unblinded outcome data were available for analysis? | | | Y |  |
| 5.2 ... multiple eligible outcome measurements (e.g. scales, definitions, time points) within the outcome domain? | | | N |  |
| 5.3 ... multiple eligible analyses of the data? | | | N |  |
| **Risk of bias judgement** | | | **Low** |  |
| **Risk of bias judgement** | | | **Low** |  |
|  |  |  |  |  |
|  |  |  |  |  |
| ID10 | **Study ID** | Wang Y et al (2019) | **Assessor** | ZK |
| Wang Y et al (2019) | **Aim** | assignment to intervention (the 'intention-to-treat' effect) |  |  |
| Intervention | **Comparator** | control | **Source** | Journal article(s) |
| Effectiveness | **Results** |  | **Weight** | 1 |
| **Signalling question** | | | **Response** | **Comments** |
| 1.1 Was the allocation sequence random? | | | Y |  |
| 1.2 Was the allocation sequence concealed until participants were enrolled and assigned to interventions? | | | Y |  |
| 1.3 Did baseline differences between intervention groups suggest a problem with the randomization process? | | | N |  |
| **Risk of bias judgement** | | | **Low** |  |
| 2.1.Were participants aware of their assigned intervention during the trial? | | | PY |  |
| 2.2.Were carers and people delivering the interventions aware of participants' assigned intervention during the trial? | | | Y |  |
| 2.3. If Y/PY/NI to 2.1 or 2.2: Were there deviations from the intended intervention that arose because of the experimental context? | | | N |  |
| 2.4 If Y/PY to 2.3: Were these deviations likely to have affected the outcome? | | | NA |  |
| 2.5. If Y/PY/NI to 2.4: Were these deviations from intended intervention balanced between groups? | | | NA |  |
| 2.6 Was an appropriate analysis used to estimate the effect of assignment to intervention? | | | Y |  |
| 2.7 If N/PN/NI to 2.6: Was there potential for a substantial impact (on the result) of the failure to analyse participants in the group to which they were randomized? | | | NA |  |
| **Risk of bias judgement** | | | **Low** |  |
| 3.1 Were data for this outcome available for all, or nearly all, participants randomized? | | | N |  |
| 3.2 If N/PN/NI to 3.1: Is there evidence that result was not biased by missing outcome data? | | | PN |  |
| 3.3 If N/PN to 3.2: Could missingness in the outcome depend on its true value? | | | N |  |
| 3.4 If Y/PY/NI to 3.3: Is it likely that missingness in the outcome depended on its true value? | | | NA |  |
| **Risk of bias judgement** | | | **Low** |  |
| 4.1 Was the method of measuring the outcome inappropriate? | | | N |  |
| 4.2 Could measurement or ascertainment of the outcome have differed between intervention groups? | | | N |  |
| 4.3 Were outcome assessors aware of the intervention received by study participants? | | | N |  |
| 4.4 If Y/PY/NI to 4.3: Could assessment of the outcome have been influenced by knowledge of intervention received? | | | NA |  |
| 4.5 If Y/PY/NI to 4.4: Is it likely that assessment of the outcome was influenced by knowledge of intervention received? | | | NA |  |
| **Risk of bias judgement** | | | **Low** |  |
| 5.1 Were the data that produced this result analysed in accordance with a pre-specified analysis plan that was finalized before unblinded outcome data were available for analysis? | | | Y |  |
| 5.2 ... multiple eligible outcome measurements (e.g. scales, definitions, time points) within the outcome domain? | | | N |  |
| 5.3 ... multiple eligible analyses of the data? | | | N |  |
| **Risk of bias judgement** | | | **Low** |  |
| **Risk of bias judgement** | | | **Low** |  |
|  |  |  |  |  |
|  |  |  |  |  |
| ID12 | **Study ID** | Liu J et al (2019) | **Assessor** | ZK |
| Liu J et al (2019) | **Aim** | assignment to intervention (the 'intention-to-treat' effect) |  |  |
| Intervention | **Comparator** | control | **Source** | Journal article(s) |
| Effectiveness | **Results** |  | **Weight** | 1 |
| **Signalling question** | | | **Response** | **Comments** |
| 1.1 Was the allocation sequence random? | | | Y |  |
| 1.2 Was the allocation sequence concealed until participants were enrolled and assigned to interventions? | | | Y |  |
| 1.3 Did baseline differences between intervention groups suggest a problem with the randomization process? | | | N |  |
| **Risk of bias judgement** | | | **Low** |  |
| 2.1.Were participants aware of their assigned intervention during the trial? | | | PY |  |
| 2.2.Were carers and people delivering the interventions aware of participants' assigned intervention during the trial? | | | Y |  |
| 2.3. If Y/PY/NI to 2.1 or 2.2: Were there deviations from the intended intervention that arose because of the experimental context? | | | N |  |
| 2.4 If Y/PY to 2.3: Were these deviations likely to have affected the outcome? | | | NA |  |
| 2.5. If Y/PY/NI to 2.4: Were these deviations from intended intervention balanced between groups? | | | NA |  |
| 2.6 Was an appropriate analysis used to estimate the effect of assignment to intervention? | | | Y |  |
| 2.7 If N/PN/NI to 2.6: Was there potential for a substantial impact (on the result) of the failure to analyse participants in the group to which they were randomized? | | | NA |  |
| **Risk of bias judgement** | | | **Low** |  |
| 3.1 Were data for this outcome available for all, or nearly all, participants randomized? | | | N |  |
| 3.2 If N/PN/NI to 3.1: Is there evidence that result was not biased by missing outcome data? | | | PN |  |
| 3.3 If N/PN to 3.2: Could missingness in the outcome depend on its true value? | | | N |  |
| 3.4 If Y/PY/NI to 3.3: Is it likely that missingness in the outcome depended on its true value? | | | NA |  |
| **Risk of bias judgement** | | | **Low** |  |
| 4.1 Was the method of measuring the outcome inappropriate? | | | N |  |
| 4.2 Could measurement or ascertainment of the outcome have differed between intervention groups? | | | N |  |
| 4.3 Were outcome assessors aware of the intervention received by study participants? | | | N |  |
| 4.4 If Y/PY/NI to 4.3: Could assessment of the outcome have been influenced by knowledge of intervention received? | | | NA |  |
| 4.5 If Y/PY/NI to 4.4: Is it likely that assessment of the outcome was influenced by knowledge of intervention received? | | | NA |  |
| **Risk of bias judgement** | | | **Low** |  |
| 5.1 Were the data that produced this result analysed in accordance with a pre-specified analysis plan that was finalized before unblinded outcome data were available for analysis? | | | Y |  |
| 5.2 ... multiple eligible outcome measurements (e.g. scales, definitions, time points) within the outcome domain? | | | N |  |
| 5.3 ... multiple eligible analyses of the data? | | | N |  |
| **Risk of bias judgement** | | | **Low** |  |
| **Risk of bias judgement** | | | **Low** |  |
|  |  |  |  |  |
|  |  |  |  |  |
| ID13 | **Study ID** | Chun EH et al (2019) | **Assessor** | ZK |
| Chun EH et al (2019) | **Aim** | assignment to intervention (the 'intention-to-treat' effect) |  |  |
| Intervention | **Comparator** | control | **Source** | Journal article(s) |
| Effectiveness | **Results** |  | **Weight** | 1 |
| **Signalling question** | | | **Response** | **Comments** |
| 1.1 Was the allocation sequence random? | | | Y |  |
| 1.2 Was the allocation sequence concealed until participants were enrolled and assigned to interventions? | | | Y |  |
| 1.3 Did baseline differences between intervention groups suggest a problem with the randomization process? | | | N |  |
| **Risk of bias judgement** | | | **Low** |  |
| 2.1.Were participants aware of their assigned intervention during the trial? | | | Y |  |
| 2.2.Were carers and people delivering the interventions aware of participants' assigned intervention during the trial? | | | Y |  |
| 2.3. If Y/PY/NI to 2.1 or 2.2: Were there deviations from the intended intervention that arose because of the experimental context? | | | PY |  |
| 2.4 If Y/PY to 2.3: Were these deviations likely to have affected the outcome? | | | PN |  |
| 2.5. If Y/PY/NI to 2.4: Were these deviations from intended intervention balanced between groups? | | | NA |  |
| 2.6 Was an appropriate analysis used to estimate the effect of assignment to intervention? | | | Y |  |
| 2.7 If N/PN/NI to 2.6: Was there potential for a substantial impact (on the result) of the failure to analyse participants in the group to which they were randomized? | | | NA |  |
| **Risk of bias judgement** | | | **Some concerns** |  |
| 3.1 Were data for this outcome available for all, or nearly all, participants randomized? | | | Y |  |
| 3.2 If N/PN/NI to 3.1: Is there evidence that result was not biased by missing outcome data? | | | NA |  |
| 3.3 If N/PN to 3.2: Could missingness in the outcome depend on its true value? | | | NA |  |
| 3.4 If Y/PY/NI to 3.3: Is it likely that missingness in the outcome depended on its true value? | | | NA |  |
| **Risk of bias judgement** | | | **Low** |  |
| 4.1 Was the method of measuring the outcome inappropriate? | | | N |  |
| 4.2 Could measurement or ascertainment of the outcome have differed between intervention groups? | | | PN |  |
| 4.3 Were outcome assessors aware of the intervention received by study participants? | | | Y |  |
| 4.4 If Y/PY/NI to 4.3: Could assessment of the outcome have been influenced by knowledge of intervention received? | | | PY |  |
| 4.5 If Y/PY/NI to 4.4: Is it likely that assessment of the outcome was influenced by knowledge of intervention received? | | | PN |  |
| **Risk of bias judgement** | | | **Some concerns** |  |
| 5.1 Were the data that produced this result analysed in accordance with a pre-specified analysis plan that was finalized before unblinded outcome data were available for analysis? | | | Y |  |
| 5.2 ... multiple eligible outcome measurements (e.g. scales, definitions, time points) within the outcome domain? | | | N |  |
| 5.3 ... multiple eligible analyses of the data? | | | N |  |
| **Risk of bias judgement** | | | **Low** |  |
| **Risk of bias judgement** | | | **Some concerns** |  |
|  |  |  |  |  |
|  |  |  |  |  |
| ID14 | **Study ID** | You AH et al (2019) | **Assessor** | ZK |
| You AH et al (2019) | **Aim** | assignment to intervention (the 'intention-to-treat' effect) |  |  |
| Intervention | **Comparator** | control | **Source** | Journal article(s) |
| Effectiveness | **Results** |  | **Weight** | 1 |
| **Signalling question** | | | **Response** | **Comments** |
| 1.1 Was the allocation sequence random? | | | Y |  |
| 1.2 Was the allocation sequence concealed until participants were enrolled and assigned to interventions? | | | Y |  |
| 1.3 Did baseline differences between intervention groups suggest a problem with the randomization process? | | | N |  |
| **Risk of bias judgement** | | | **Low** |  |
| 2.1.Were participants aware of their assigned intervention during the trial? | | | PY |  |
| 2.2.Were carers and people delivering the interventions aware of participants' assigned intervention during the trial? | | | Y |  |
| 2.3. If Y/PY/NI to 2.1 or 2.2: Were there deviations from the intended intervention that arose because of the experimental context? | | | N |  |
| 2.4 If Y/PY to 2.3: Were these deviations likely to have affected the outcome? | | | NA |  |
| 2.5. If Y/PY/NI to 2.4: Were these deviations from intended intervention balanced between groups? | | | NA |  |
| 2.6 Was an appropriate analysis used to estimate the effect of assignment to intervention? | | | Y |  |
| 2.7 If N/PN/NI to 2.6: Was there potential for a substantial impact (on the result) of the failure to analyse participants in the group to which they were randomized? | | | NA |  |
| **Risk of bias judgement** | | | **Low** |  |
| 3.1 Were data for this outcome available for all, or nearly all, participants randomized? | | | N |  |
| 3.2 If N/PN/NI to 3.1: Is there evidence that result was not biased by missing outcome data? | | | PN |  |
| 3.3 If N/PN to 3.2: Could missingness in the outcome depend on its true value? | | | N |  |
| 3.4 If Y/PY/NI to 3.3: Is it likely that missingness in the outcome depended on its true value? | | | NA |  |
| **Risk of bias judgement** | | | **Low** |  |
| 4.1 Was the method of measuring the outcome inappropriate? | | | N |  |
| 4.2 Could measurement or ascertainment of the outcome have differed between intervention groups? | | | N |  |
| 4.3 Were outcome assessors aware of the intervention received by study participants? | | | N |  |
| 4.4 If Y/PY/NI to 4.3: Could assessment of the outcome have been influenced by knowledge of intervention received? | | | NA |  |
| 4.5 If Y/PY/NI to 4.4: Is it likely that assessment of the outcome was influenced by knowledge of intervention received? | | | NA |  |
| **Risk of bias judgement** | | | **Low** |  |
| 5.1 Were the data that produced this result analysed in accordance with a pre-specified analysis plan that was finalized before unblinded outcome data were available for analysis? | | | Y |  |
| 5.2 ... multiple eligible outcome measurements (e.g. scales, definitions, time points) within the outcome domain? | | | N |  |
| 5.3 ... multiple eligible analyses of the data? | | | N |  |
| **Risk of bias judgement** | | | **Low** |  |
| **Risk of bias judgement** | | | **Low** |  |
|  |  |  |  |  |
|  |  |  |  |  |
| ID15 | **Study ID** | Atashkhoei S et al (2020) | **Assessor** | ZK |
| Atashkhoei S et al (2020) | **Aim** | assignment to intervention (the 'intention-to-treat' effect) |  |  |
| Intervention | **Comparator** | control | **Source** | Journal article(s) |
| Effectiveness | **Results** |  | **Weight** | 1 |
| **Signalling question** | | | **Response** | **Comments** |
| 1.1 Was the allocation sequence random? | | | Y |  |
| 1.2 Was the allocation sequence concealed until participants were enrolled and assigned to interventions? | | | Y |  |
| 1.3 Did baseline differences between intervention groups suggest a problem with the randomization process? | | | N |  |
| **Risk of bias judgement** | | | **Low** |  |
| 2.1.Were participants aware of their assigned intervention during the trial? | | | Y |  |
| 2.2.Were carers and people delivering the interventions aware of participants' assigned intervention during the trial? | | | Y |  |
| 2.3. If Y/PY/NI to 2.1 or 2.2: Were there deviations from the intended intervention that arose because of the experimental context? | | | N |  |
| 2.4 If Y/PY to 2.3: Were these deviations likely to have affected the outcome? | | | NA |  |
| 2.5. If Y/PY/NI to 2.4: Were these deviations from intended intervention balanced between groups? | | | NA |  |
| 2.6 Was an appropriate analysis used to estimate the effect of assignment to intervention? | | | Y |  |
| 2.7 If N/PN/NI to 2.6: Was there potential for a substantial impact (on the result) of the failure to analyse participants in the group to which they were randomized? | | | NA |  |
| **Risk of bias judgement** | | | **Low** |  |
| 3.1 Were data for this outcome available for all, or nearly all, participants randomized? | | | NI |  |
| 3.2 If N/PN/NI to 3.1: Is there evidence that result was not biased by missing outcome data? | | | Y |  |
| 3.3 If N/PN to 3.2: Could missingness in the outcome depend on its true value? | | | NA |  |
| 3.4 If Y/PY/NI to 3.3: Is it likely that missingness in the outcome depended on its true value? | | | NA |  |
| **Risk of bias judgement** | | | **Low** |  |
| 4.1 Was the method of measuring the outcome inappropriate? | | | N |  |
| 4.2 Could measurement or ascertainment of the outcome have differed between intervention groups? | | | N |  |
| 4.3 Were outcome assessors aware of the intervention received by study participants? | | | NI |  |
| 4.4 If Y/PY/NI to 4.3: Could assessment of the outcome have been influenced by knowledge of intervention received? | | | N |  |
| 4.5 If Y/PY/NI to 4.4: Is it likely that assessment of the outcome was influenced by knowledge of intervention received? | | | NA |  |
| **Risk of bias judgement** | | | **Low** |  |
| 5.1 Were the data that produced this result analysed in accordance with a pre-specified analysis plan that was finalized before unblinded outcome data were available for analysis? | | | Y |  |
| 5.2 ... multiple eligible outcome measurements (e.g. scales, definitions, time points) within the outcome domain? | | | N |  |
| 5.3 ... multiple eligible analyses of the data? | | | N |  |
| **Risk of bias judgement** | | | **Low** |  |
| **Risk of bias judgement** | | | **Low** |  |
|  |  |  |  |  |
|  |  |  |  |  |
| ID16 | **Study ID** | Shono A et al (2020) | **Assessor** | ZK |
| Shono A et al (2020) | **Aim** | assignment to intervention (the 'intention-to-treat' effect) |  |  |
| Intervention | **Comparator** | control | **Source** | Journal article(s) |
| Effectiveness | **Results** |  | **Weight** | 1 |
| **Signalling question** | | | **Response** | **Comments** |
| 1.1 Was the allocation sequence random? | | | Y |  |
| 1.2 Was the allocation sequence concealed until participants were enrolled and assigned to interventions? | | | Y |  |
| 1.3 Did baseline differences between intervention groups suggest a problem with the randomization process? | | | N |  |
| **Risk of bias judgement** | | | **Low** |  |
| 2.1.Were participants aware of their assigned intervention during the trial? | | | PY |  |
| 2.2.Were carers and people delivering the interventions aware of participants' assigned intervention during the trial? | | | PY |  |
| 2.3. If Y/PY/NI to 2.1 or 2.2: Were there deviations from the intended intervention that arose because of the experimental context? | | | PY |  |
| 2.4 If Y/PY to 2.3: Were these deviations likely to have affected the outcome? | | | N |  |
| 2.5. If Y/PY/NI to 2.4: Were these deviations from intended intervention balanced between groups? | | | NA |  |
| 2.6 Was an appropriate analysis used to estimate the effect of assignment to intervention? | | | Y |  |
| 2.7 If N/PN/NI to 2.6: Was there potential for a substantial impact (on the result) of the failure to analyse participants in the group to which they were randomized? | | | NA |  |
| **Risk of bias judgement** | | | **Some concerns** |  |
| 3.1 Were data for this outcome available for all, or nearly all, participants randomized? | | | Y |  |
| 3.2 If N/PN/NI to 3.1: Is there evidence that result was not biased by missing outcome data? | | | NA |  |
| 3.3 If N/PN to 3.2: Could missingness in the outcome depend on its true value? | | | NA |  |
| 3.4 If Y/PY/NI to 3.3: Is it likely that missingness in the outcome depended on its true value? | | | NA |  |
| **Risk of bias judgement** | | | **Low** |  |
| 4.1 Was the method of measuring the outcome inappropriate? | | | N |  |
| 4.2 Could measurement or ascertainment of the outcome have differed between intervention groups? | | | N |  |
| 4.3 Were outcome assessors aware of the intervention received by study participants? | | | Y |  |
| 4.4 If Y/PY/NI to 4.3: Could assessment of the outcome have been influenced by knowledge of intervention received? | | | PY |  |
| 4.5 If Y/PY/NI to 4.4: Is it likely that assessment of the outcome was influenced by knowledge of intervention received? | | | N |  |
| **Risk of bias judgement** | | | **Some concerns** |  |
| 5.1 Were the data that produced this result analysed in accordance with a pre-specified analysis plan that was finalized before unblinded outcome data were available for analysis? | | | Y |  |
| 5.2 ... multiple eligible outcome measurements (e.g. scales, definitions, time points) within the outcome domain? | | | N |  |
| 5.3 ... multiple eligible analyses of the data? | | | N |  |
| **Risk of bias judgement** | | | **Low** |  |
| **Risk of bias judgement** | | | **Low** |  |
|  |  |  |  |  |
|  |  |  |  |  |
| ID17 | **Study ID** | Piriyapatsom A et al (2020) | **Assessor** | ZK |
| Piriyapatsom A et al (2020) | **Aim** | assignment to intervention (the 'intention-to-treat' effect) |  |  |
| Intervention | **Comparator** | control | **Source** | Journal article(s) |
| Effectiveness | **Results** |  | **Weight** | 1 |
| **Signalling question** | | | **Response** | **Comments** |
| 1.1 Was the allocation sequence random? | | | Y |  |
| 1.2 Was the allocation sequence concealed until participants were enrolled and assigned to interventions? | | | Y |  |
| 1.3 Did baseline differences between intervention groups suggest a problem with the randomization process? | | | N |  |
| **Risk of bias judgement** | | | **Low** |  |
| 2.1.Were participants aware of their assigned intervention during the trial? | | | PN |  |
| 2.2.Were carers and people delivering the interventions aware of participants' assigned intervention during the trial? | | | Y |  |
| 2.3. If Y/PY/NI to 2.1 or 2.2: Were there deviations from the intended intervention that arose because of the experimental context? | | | PY |  |
| 2.4 If Y/PY to 2.3: Were these deviations likely to have affected the outcome? | | | PY |  |
| 2.5. If Y/PY/NI to 2.4: Were these deviations from intended intervention balanced between groups? | | | NI |  |
| 2.6 Was an appropriate analysis used to estimate the effect of assignment to intervention? | | | Y |  |
| 2.7 If N/PN/NI to 2.6: Was there potential for a substantial impact (on the result) of the failure to analyse participants in the group to which they were randomized? | | | NA |  |
| **Risk of bias judgement** | | | **High** |  |
| 3.1 Were data for this outcome available for all, or nearly all, participants randomized? | | | Y |  |
| 3.2 If N/PN/NI to 3.1: Is there evidence that result was not biased by missing outcome data? | | | NA |  |
| 3.3 If N/PN to 3.2: Could missingness in the outcome depend on its true value? | | | NA |  |
| 3.4 If Y/PY/NI to 3.3: Is it likely that missingness in the outcome depended on its true value? | | | NA |  |
| **Risk of bias judgement** | | | **Low** |  |
| 4.1 Was the method of measuring the outcome inappropriate? | | | N |  |
| 4.2 Could measurement or ascertainment of the outcome have differed between intervention groups? | | | PN |  |
| 4.3 Were outcome assessors aware of the intervention received by study participants? | | | Y |  |
| 4.4 If Y/PY/NI to 4.3: Could assessment of the outcome have been influenced by knowledge of intervention received? | | | NI |  |
| 4.5 If Y/PY/NI to 4.4: Is it likely that assessment of the outcome was influenced by knowledge of intervention received? | | | NI |  |
| **Risk of bias judgement** | | | **High** |  |
| 5.1 Were the data that produced this result analysed in accordance with a pre-specified analysis plan that was finalized before unblinded outcome data were available for analysis? | | | Y |  |
| 5.2 ... multiple eligible outcome measurements (e.g. scales, definitions, time points) within the outcome domain? | | | N |  |
| 5.3 ... multiple eligible analyses of the data? | | | N |  |
| **Risk of bias judgement** | | | **Low** |  |
| **Risk of bias judgement** | | | **High** |  |
|  |  |  |  |  |
|  |  |  |  |  |
| ID18 | **Study ID** | Cammarota G et al (2020) | **Assessor** | ZK |
| Cammarota G et al (2020) | **Aim** | assignment to intervention (the 'intention-to-treat' effect) |  |  |
| Intervention | **Comparator** | control | **Source** | Journal article(s) |
| Effectiveness | **Results** |  | **Weight** | 1 |
| **Signalling question** | | | **Response** | **Comments** |
| 1.1 Was the allocation sequence random? | | | Y |  |
| 1.2 Was the allocation sequence concealed until participants were enrolled and assigned to interventions? | | | Y |  |
| 1.3 Did baseline differences between intervention groups suggest a problem with the randomization process? | | | N |  |
| **Risk of bias judgement** | | | **Low** |  |
| 2.1.Were participants aware of their assigned intervention during the trial? | | | N |  |
| 2.2.Were carers and people delivering the interventions aware of participants' assigned intervention during the trial? | | | N |  |
| 2.3. If Y/PY/NI to 2.1 or 2.2: Were there deviations from the intended intervention that arose because of the experimental context? | | | NA |  |
| 2.4 If Y/PY to 2.3: Were these deviations likely to have affected the outcome? | | | NA |  |
| 2.5. If Y/PY/NI to 2.4: Were these deviations from intended intervention balanced between groups? | | | NA |  |
| 2.6 Was an appropriate analysis used to estimate the effect of assignment to intervention? | | | Y |  |
| 2.7 If N/PN/NI to 2.6: Was there potential for a substantial impact (on the result) of the failure to analyse participants in the group to which they were randomized? | | | NA |  |
| **Risk of bias judgement** | | | **Low** |  |
| 3.1 Were data for this outcome available for all, or nearly all, participants randomized? | | | Y |  |
| 3.2 If N/PN/NI to 3.1: Is there evidence that result was not biased by missing outcome data? | | | NA |  |
| 3.3 If N/PN to 3.2: Could missingness in the outcome depend on its true value? | | | NA |  |
| 3.4 If Y/PY/NI to 3.3: Is it likely that missingness in the outcome depended on its true value? | | | NA |  |
| **Risk of bias judgement** | | | **Low** |  |
| 4.1 Was the method of measuring the outcome inappropriate? | | | N |  |
| 4.2 Could measurement or ascertainment of the outcome have differed between intervention groups? | | | N |  |
| 4.3 Were outcome assessors aware of the intervention received by study participants? | | | N |  |
| 4.4 If Y/PY/NI to 4.3: Could assessment of the outcome have been influenced by knowledge of intervention received? | | | NA |  |
| 4.5 If Y/PY/NI to 4.4: Is it likely that assessment of the outcome was influenced by knowledge of intervention received? | | | NA |  |
| **Risk of bias judgement** | | | **Low** |  |
| 5.1 Were the data that produced this result analysed in accordance with a pre-specified analysis plan that was finalized before unblinded outcome data were available for analysis? | | | Y |  |
| 5.2 ... multiple eligible outcome measurements (e.g. scales, definitions, time points) within the outcome domain? | | | N |  |
| 5.3 ... multiple eligible analyses of the data? | | | N |  |
| **Risk of bias judgement** | | | **Low** |  |
| **Risk of bias judgement** | | | **Low** |  |
|  |  |  |  |  |
|  |  |  |  |  |
| ID19 | **Study ID** | Girrbach F et al (2020) | **Assessor** | ZK |
| Girrbach F et al (2020) | **Aim** | assignment to intervention (the 'intention-to-treat' effect) |  |  |
| Intervention | **Comparator** | control | **Source** | Journal article(s) |
| Effectiveness | **Results** |  | **Weight** | 1 |
| **Signalling question** | | | **Response** | **Comments** |
| 1.1 Was the allocation sequence random? | | | Y |  |
| 1.2 Was the allocation sequence concealed until participants were enrolled and assigned to interventions? | | | Y |  |
| 1.3 Did baseline differences between intervention groups suggest a problem with the randomization process? | | | N |  |
| **Risk of bias judgement** | | | **Low** |  |
| 2.1.Were participants aware of their assigned intervention during the trial? | | | N |  |
| 2.2.Were carers and people delivering the interventions aware of participants' assigned intervention during the trial? | | | N |  |
| 2.3. If Y/PY/NI to 2.1 or 2.2: Were there deviations from the intended intervention that arose because of the experimental context? | | | NA |  |
| 2.4 If Y/PY to 2.3: Were these deviations likely to have affected the outcome? | | | NA |  |
| 2.5. If Y/PY/NI to 2.4: Were these deviations from intended intervention balanced between groups? | | | NA |  |
| 2.6 Was an appropriate analysis used to estimate the effect of assignment to intervention? | | | Y |  |
| 2.7 If N/PN/NI to 2.6: Was there potential for a substantial impact (on the result) of the failure to analyse participants in the group to which they were randomized? | | | NA |  |
| **Risk of bias judgement** | | | **Low** |  |
| 3.1 Were data for this outcome available for all, or nearly all, participants randomized? | | | Y |  |
| 3.2 If N/PN/NI to 3.1: Is there evidence that result was not biased by missing outcome data? | | | NA |  |
| 3.3 If N/PN to 3.2: Could missingness in the outcome depend on its true value? | | | NA |  |
| 3.4 If Y/PY/NI to 3.3: Is it likely that missingness in the outcome depended on its true value? | | | NA |  |
| **Risk of bias judgement** | | | **Low** |  |
| 4.1 Was the method of measuring the outcome inappropriate? | | | N |  |
| 4.2 Could measurement or ascertainment of the outcome have differed between intervention groups? | | | N |  |
| 4.3 Were outcome assessors aware of the intervention received by study participants? | | | N |  |
| 4.4 If Y/PY/NI to 4.3: Could assessment of the outcome have been influenced by knowledge of intervention received? | | | NA |  |
| 4.5 If Y/PY/NI to 4.4: Is it likely that assessment of the outcome was influenced by knowledge of intervention received? | | | NA |  |
| **Risk of bias judgement** | | | **Low** |  |
| 5.1 Were the data that produced this result analysed in accordance with a pre-specified analysis plan that was finalized before unblinded outcome data were available for analysis? | | | Y |  |
| 5.2 ... multiple eligible outcome measurements (e.g. scales, definitions, time points) within the outcome domain? | | | N |  |
| 5.3 ... multiple eligible analyses of the data? | | | N |  |
| **Risk of bias judgement** | | | **Low** |  |
| **Risk of bias judgement** | | | **Low** |  |
|  |  |  |  |  |
|  |  |  |  |  |
| ID20 | **Study ID** | Li H et al (2021) | **Assessor** | ZK |
| Li H et al (2021) | **Aim** | assignment to intervention (the 'intention-to-treat' effect) |  |  |
| Intevention | **Comparator** | control | **Source** | Journal article(s) |
| Effectiveness | **Results** |  | **Weight** | 1 |
| **Signalling question** | | | **Response** | **Comments** |
| 1.1 Was the allocation sequence random? | | | Y |  |
| 1.2 Was the allocation sequence concealed until participants were enrolled and assigned to interventions? | | | Y |  |
| 1.3 Did baseline differences between intervention groups suggest a problem with the randomization process? | | | N |  |
| **Risk of bias judgement** | | | **Low** |  |
| 2.1.Were participants aware of their assigned intervention during the trial? | | | PY |  |
| 2.2.Were carers and people delivering the interventions aware of participants' assigned intervention during the trial? | | | N |  |
| 2.3. If Y/PY/NI to 2.1 or 2.2: Were there deviations from the intended intervention that arose because of the experimental context? | | | N |  |
| 2.4 If Y/PY to 2.3: Were these deviations likely to have affected the outcome? | | | NA |  |
| 2.5. If Y/PY/NI to 2.4: Were these deviations from intended intervention balanced between groups? | | | NA |  |
| 2.6 Was an appropriate analysis used to estimate the effect of assignment to intervention? | | | Y |  |
| 2.7 If N/PN/NI to 2.6: Was there potential for a substantial impact (on the result) of the failure to analyse participants in the group to which they were randomized? | | | NA |  |
| **Risk of bias judgement** | | | **Low** |  |
| 3.1 Were data for this outcome available for all, or nearly all, participants randomized? | | | Y |  |
| 3.2 If N/PN/NI to 3.1: Is there evidence that result was not biased by missing outcome data? | | | NA |  |
| 3.3 If N/PN to 3.2: Could missingness in the outcome depend on its true value? | | | NA |  |
| 3.4 If Y/PY/NI to 3.3: Is it likely that missingness in the outcome depended on its true value? | | | NA |  |
| **Risk of bias judgement** | | | **Low** |  |
| 4.1 Was the method of measuring the outcome inappropriate? | | | N |  |
| 4.2 Could measurement or ascertainment of the outcome have differed between intervention groups? | | | N |  |
| 4.3 Were outcome assessors aware of the intervention received by study participants? | | | N |  |
| 4.4 If Y/PY/NI to 4.3: Could assessment of the outcome have been influenced by knowledge of intervention received? | | | NA |  |
| 4.5 If Y/PY/NI to 4.4: Is it likely that assessment of the outcome was influenced by knowledge of intervention received? | | | NA |  |
| **Risk of bias judgement** | | | **Low** |  |
| 5.1 Were the data that produced this result analysed in accordance with a pre-specified analysis plan that was finalized before unblinded outcome data were available for analysis? | | | Y |  |
| 5.2 ... multiple eligible outcome measurements (e.g. scales, definitions, time points) within the outcome domain? | | | N |  |
| 5.3 ... multiple eligible analyses of the data? | | | N |  |
| **Risk of bias judgement** | | | **Low** |  |
| **Risk of bias judgement** | | | **Low** |  |
|  |  |  |  |  |
|  |  |  |  |  |
| ID21 | **Study ID** | Nguyen TK et al (2021) | **Assessor** | ZK |
| Nguyen TK et al (2021) | **Aim** | assignment to intervention (the 'intention-to-treat' effect) |  |  |
| Intervention | **Comparator** | control | **Source** | Journal article(s) |
| Effectiveness | **Results** |  | **Weight** | 1 |
| **Signalling question** | | | **Response** | **Comments** |
| 1.1 Was the allocation sequence random? | | | Y |  |
| 1.2 Was the allocation sequence concealed until participants were enrolled and assigned to interventions? | | | Y |  |
| 1.3 Did baseline differences between intervention groups suggest a problem with the randomization process? | | | N |  |
| **Risk of bias judgement** | | | **Low** |  |
| 2.1.Were participants aware of their assigned intervention during the trial? | | | N |  |
| 2.2.Were carers and people delivering the interventions aware of participants' assigned intervention during the trial? | | | N |  |
| 2.3. If Y/PY/NI to 2.1 or 2.2: Were there deviations from the intended intervention that arose because of the experimental context? | | | NA |  |
| 2.4 If Y/PY to 2.3: Were these deviations likely to have affected the outcome? | | | NA |  |
| 2.5. If Y/PY/NI to 2.4: Were these deviations from intended intervention balanced between groups? | | | NA |  |
| 2.6 Was an appropriate analysis used to estimate the effect of assignment to intervention? | | | Y |  |
| 2.7 If N/PN/NI to 2.6: Was there potential for a substantial impact (on the result) of the failure to analyse participants in the group to which they were randomized? | | | NA |  |
| **Risk of bias judgement** | | | **Low** |  |
| 3.1 Were data for this outcome available for all, or nearly all, participants randomized? | | | Y |  |
| 3.2 If N/PN/NI to 3.1: Is there evidence that result was not biased by missing outcome data? | | | NA |  |
| 3.3 If N/PN to 3.2: Could missingness in the outcome depend on its true value? | | | NA |  |
| 3.4 If Y/PY/NI to 3.3: Is it likely that missingness in the outcome depended on its true value? | | | NA |  |
| **Risk of bias judgement** | | | **Low** |  |
| 4.1 Was the method of measuring the outcome inappropriate? | | | N |  |
| 4.2 Could measurement or ascertainment of the outcome have differed between intervention groups? | | | NI |  |
| 4.3 Were outcome assessors aware of the intervention received by study participants? | | | N |  |
| 4.4 If Y/PY/NI to 4.3: Could assessment of the outcome have been influenced by knowledge of intervention received? | | | NA |  |
| 4.5 If Y/PY/NI to 4.4: Is it likely that assessment of the outcome was influenced by knowledge of intervention received? | | | NA |  |
| **Risk of bias judgement** | | | **Some concerns** |  |
| 5.1 Were the data that produced this result analysed in accordance with a pre-specified analysis plan that was finalized before unblinded outcome data were available for analysis? | | | Y |  |
| 5.2 ... multiple eligible outcome measurements (e.g. scales, definitions, time points) within the outcome domain? | | | NI |  |
| 5.3 ... multiple eligible analyses of the data? | | | N |  |
| **Risk of bias judgement** | | | **Some concerns** |  |
| **Risk of bias judgement** | | | **Some concerns** |  |
|  |  |  |  |  |
|  |  |  |  |  |
| ID22 | **Study ID** | Huang D et al (2021) | **Assessor** | ZK |
| Huang D et al (2021) | **Aim** | assignment to intervention (the 'intention-to-treat' effect) |  |  |
| Intervention | **Comparator** | control | **Source** | Journal article(s) |
| Effectiveness | **Results** |  | **Weight** | 1 |
| **Signalling question** | | | **Response** | **Comments** |
| 1.1 Was the allocation sequence random? | | | Y |  |
| 1.2 Was the allocation sequence concealed until participants were enrolled and assigned to interventions? | | | Y |  |
| 1.3 Did baseline differences between intervention groups suggest a problem with the randomization process? | | | N |  |
| **Risk of bias judgement** | | | **Low** |  |
| 2.1.Were participants aware of their assigned intervention during the trial? | | | PN |  |
| 2.2.Were carers and people delivering the interventions aware of participants' assigned intervention during the trial? | | | PN |  |
| 2.3. If Y/PY/NI to 2.1 or 2.2: Were there deviations from the intended intervention that arose because of the experimental context? | | | NA |  |
| 2.4 If Y/PY to 2.3: Were these deviations likely to have affected the outcome? | | | NA |  |
| 2.5. If Y/PY/NI to 2.4: Were these deviations from intended intervention balanced between groups? | | | NA |  |
| 2.6 Was an appropriate analysis used to estimate the effect of assignment to intervention? | | | Y |  |
| 2.7 If N/PN/NI to 2.6: Was there potential for a substantial impact (on the result) of the failure to analyse participants in the group to which they were randomized? | | | NA |  |
| **Risk of bias judgement** | | | **Low** |  |
| 3.1 Were data for this outcome available for all, or nearly all, participants randomized? | | | Y |  |
| 3.2 If N/PN/NI to 3.1: Is there evidence that result was not biased by missing outcome data? | | | NA |  |
| 3.3 If N/PN to 3.2: Could missingness in the outcome depend on its true value? | | | NA |  |
| 3.4 If Y/PY/NI to 3.3: Is it likely that missingness in the outcome depended on its true value? | | | NA |  |
| **Risk of bias judgement** | | | **Low** |  |
| 4.1 Was the method of measuring the outcome inappropriate? | | | PN |  |
| 4.2 Could measurement or ascertainment of the outcome have differed between intervention groups? | | | N |  |
| 4.3 Were outcome assessors aware of the intervention received by study participants? | | | N |  |
| 4.4 If Y/PY/NI to 4.3: Could assessment of the outcome have been influenced by knowledge of intervention received? | | | NA |  |
| 4.5 If Y/PY/NI to 4.4: Is it likely that assessment of the outcome was influenced by knowledge of intervention received? | | | NA |  |
| **Risk of bias judgement** | | | **Low** |  |
| 5.1 Were the data that produced this result analysed in accordance with a pre-specified analysis plan that was finalized before unblinded outcome data were available for analysis? | | | Y |  |
| 5.2 ... multiple eligible outcome measurements (e.g. scales, definitions, time points) within the outcome domain? | | | N |  |
| 5.3 ... multiple eligible analyses of the data? | | | N |  |
| **Risk of bias judgement** | | | **Low** |  |
| **Risk of bias judgement** | | | **Low** |  |

**Table 1. Risk of bias judgment for each study.**

Table presented the quality of the included studies in five domains: randomization process, deviation from intended interventions, missing outcome data, outcome measurement, and selection of reported results. Each domain contains “Signaling questions” to facilitate risk of bias judgments and was rated as high risk, low risk, or some concern using the Cochrane Criteria for assessing the risk of bias.

**Abbreviations:** Y – yes, N – no, PY – probably yes, PN – probably no, NI – no information, NA – not applicable**. .**
